# Confinement of Luminescent Guests in Metal–Organic Frameworks: Understanding Pathways from Synthesis and Multimodal Characterization to Potential Applications of LG@MOF Systems

**DOI:** 10.1021/acs.chemrev.1c00980

**Published:** 2022-04-15

**Authors:** Mario Gutiérrez, Yang Zhang, Jin-Chong Tan

**Affiliations:** † Multifunctional Materials & Composites (MMC) Laboratory, Department of Engineering Science, University of Oxford, Parks Road, Oxford OX1 3PJ, United Kingdom; ‡ Departamento de Química Física, Facultad de Ciencias Ambientales y Bioquímica, INAMOL, Universidad de Castilla-La Mancha, Avenida Carlos III, S/N, 45071 Toledo, Spain

## Abstract

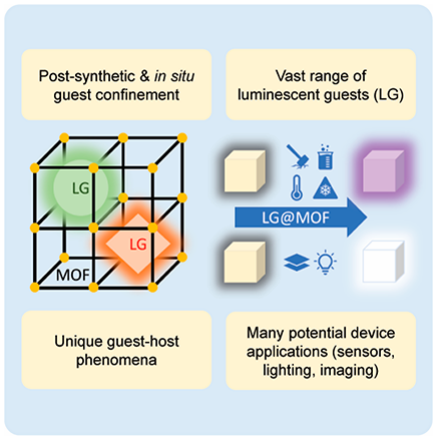

This review gives an authoritative, critical, and accessible overview of an emergent class of fluorescent materials termed “LG@MOF”, engineered from the nanoscale confinement of luminescent guests (LG) in a metal–organic framework (MOF) host, realizing a myriad of unconventional materials with fascinating photophysical and photochemical properties. We begin by summarizing the synthetic methodologies and design guidelines for representative LG@MOF systems, where the major types of fluorescent guest encompass organic dyes, metal ions, metal complexes, metal nanoclusters, quantum dots, and hybrid perovskites. Subsequently, we discuss the methods for characterizing the resultant guest–host structures, guest loading, photophysical properties, and review local-scale techniques recently employed to elucidate guest positions. A special emphasis is paid to the pros and cons of the various methods in the context of LG@MOF. In the following section, we provide a brief tutorial on the basic guest–host phenomena, focusing on the excited state events and nanoscale confinement effects underpinning the exceptional behavior of LG@MOF systems. The review finally culminates in the most striking applications of LG@MOF materials, particularly the “turn-on” type fluorochromic chemo- and mechano-sensors, noninvasive thermometry and optical pH sensors, electroluminescence, and innovative security devices. This review offers a comprehensive coverage of general interest to the multidisciplinary materials community to stimulate frontier research in the vibrant sector of light-emitting MOF composite systems.

## 1 Introduction

The pace of progress witnessed in the field of metal–organic frameworks or porous coordination polymers (MOFs or PCPs) is accelerating, transforming it into a central subject of interdisciplinary research worldwide. While it first began in the realm of synthetic chemistry, in the past 25 years^1^ the research on MOF materials has propagated to materials science, engineering, physics, biomedicine, nanotechnology, and other cognate disciplines. MOFs are nanoporous hybrid materials constructed from the self-assembly of organic and inorganic basic building blocks. In essence, MOFs are crystalline compounds with long-range periodic order, whose extended 3-D structures are comprising metal nodes (ions or clusters) connected by multitopic organic linkers. This bottom-up synthetic process generates a cornucopia of network topologies and framework architectures^2^ held together by strong directional bonds,^3^ yielding porous frameworks with a well-defined cavity size (typically ca. 0.2–3 nm). Starting from a variety of organic and inorganic building units, in principle, one could design, engineer, and tune a plethora of physical and chemical properties which cannot be obtained from purely organic and inorganic systems alone.^4^

Many MOFs are appreciably more porous than commercial nanoporous adsorbents, such as zeolites, silica gels, and activated carbons.^5^ Naturally, the first practical uses identified for MOFs have included gas storage and separation, CO_2_ capture, and catalysis, which may be perceived as traditional applications for nanoporous materials. However, the potential of MOFs is not just limited to the foregoing applications, and during the past decade there has been a growing interest in the development of a variety of guest-encapsulated MOF systems (Guest@MOF)^6^ and luminescent MOF materials (LMOFs).^7,8^ Indeed, LMOFs and composites have been demonstrated as promising candidates for optoelectronic and photonic applications (e.g., chemical and temperature sensors, solid-state lighting, biomarkers, anticounterfeiting) hence driving a host of research activities aimed at this direction.^9−15^

One unique way to design and fabricate LMOFs is to harness the porosity of the MOF host to afford the encapsulation of a variety of luminescent guests (LG), resulting in the “LG@MOF” composite system (i.e., a special case of Guest@MOF). Guest encapsulation into MOFs offers certain advantages over traditional syntheses of luminescent materials, such as the facile and cost effectiveness of this methodology, the possibility of tuning the emission properties by a rational selection of off-the-shelf fluorophores or luminescent dyes, and the prevention of aggregation-caused quenching (ACQ) phenomena via partitioning of the luminescent guests into the pores of the crystalline MOF host. Although the number of reviews on the subject of luminescent MOFs is growing steadily, the majority of them are focused on LMOF materials with intrinsic luminescence.^16−19^ In this review, therefore, we shall concentrate our efforts on recent exemplars pertaining to the LG@MOF systems, which have substantially pushed the boundaries of LMOFs and offer exciting new opportunities as a platform to engineer bespoke materials for real-world applications.

The review will be organized as follows. Section 2 will introduce the readers to the primary synthetic protocols for the fabrication of LG@MOF materials, where the confinement of luminescent guests through the *in situ* and postsynthetic pathways will be discussed and contrasted. In Section 3, the topical luminescent guests that have been encapsulated within the MOF hosts are summarized and cogently explained. Section 4 is dedicated to the materials characterization methods most applicable for studying LG@MOFs, in which special attention is paid to elaborating their strengths and limitations, plus opportunities where relevant. The key limitations will be addressed, for instance in confirming pore encapsulation of the guests (rather than surface adhesion), and stressing the need to invoke complementary characterization techniques to confirm success of a chosen guest confinement/encapsulation strategy. In Section 5, we present a concise tutorial on the fundamental guest–host phenomena and excited state events; importantly, our intention here is to give the general reader a better understanding and thus appreciation of the possible mechanisms underscoring the fascinating behavior observed in LG@MOF systems. Finally in Section 6, we highlight some of the most exciting emergent applications arising from the concept of LG@MOFs, including the construction of electroluminescent MOF-LED devices. We shall elucidate how the mechanochromic, solvatochromic, and thermochromic properties of LG@MOFs can be harnessed for sensing physical and chemical stimuli, putting a special emphasis on pioneering optical sensors enabled by the “turn-on” and ratiometric mechanisms.

## 2 Synthetic Protocols and Design Guidelines

**Figure 1:**
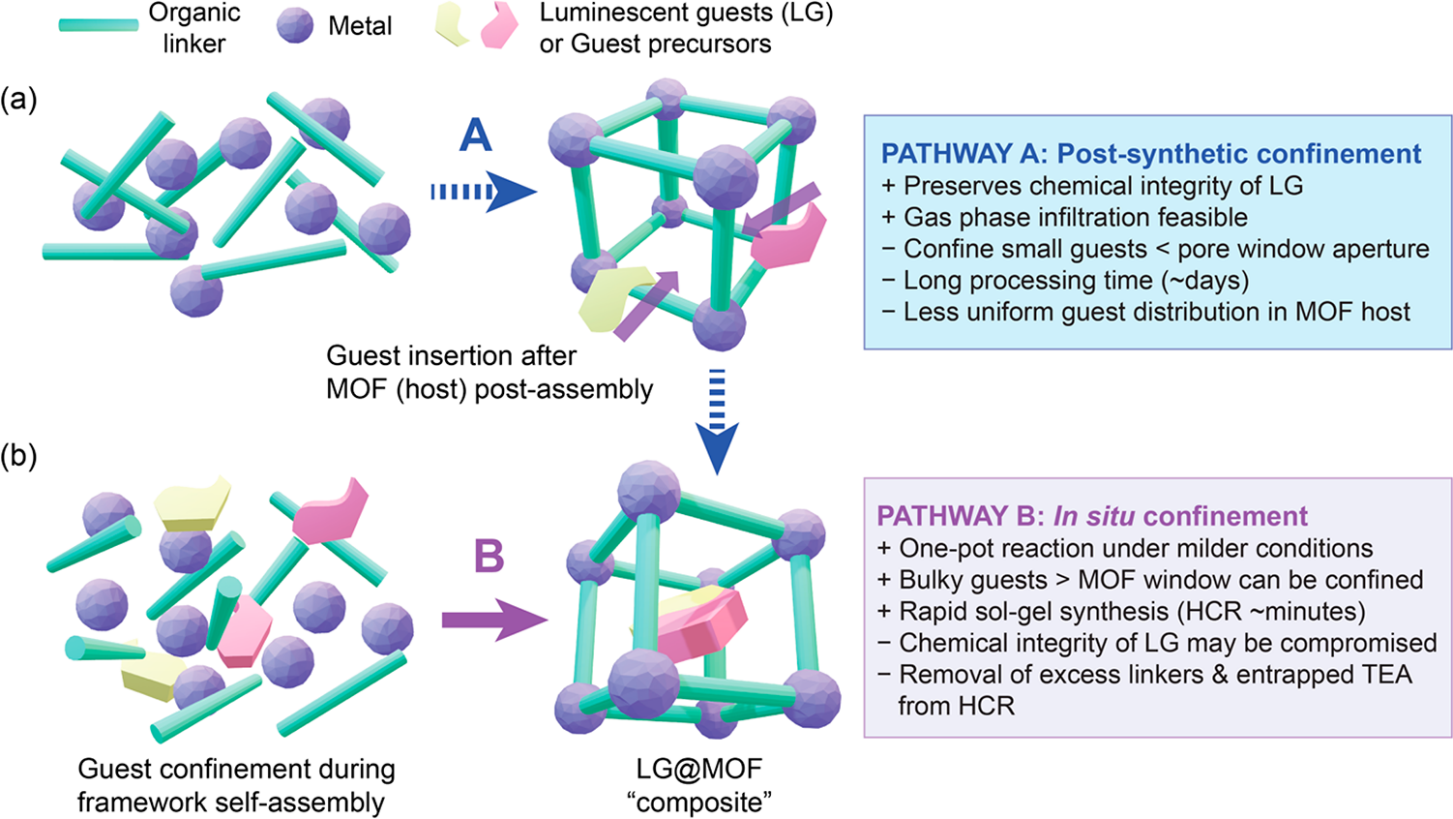
Schematics illustrating the synthetic pathways of the LG@MOF composite system. (a) Postsynthetic confinement, and (b) *in situ* confinement methodologies to achieve the incarceration of luminescent guests (LG) or guest precursors within the porous MOF host structure. The right panels summarize some of the pros and cons of these guest–host confinement strategies. (TEA = triethylamine, HCR = high-concentration reaction).

The synthetic methodologies employed have an important impact on the resulting structures and performance of the LG@MOF systems. With regards to the synthesis of pristine MOF materials, the available methods have been fully described and summarized by many comprehensive reviews.^20−24^ In the light of this, for this section we will focus on explaining and discussing the synthetic routes to yield the LG@MOF “composite” systems only. Currently, the synthesis of this family of luminescent guest-encapsulated systems can be broadly divided into two major categories. The first approach is termed the “post-synthesis” method as depicted in Figure 1a, while the second strategy illustrated in Figure 1b is designated as the “*in situ* synthesis” method. In the sections that follow, we shall evaluate the basic concepts and general applicability, discussing the advantages and disadvantages of the fabrication pathways by means of representative examples to enable the readers to develop a better understanding of the processes involved for fabricating LG@MOF materials.

### 2.1 Postsynthetic LG Confinement Methods

The postsynthesis methodology begins with the standard synthesis to yield the MOF structure first, followed by sample washing and evacuation or activation steps. Subsequently, the preassembled MOF is subjected to a separate process where the insertion of the luminescent guest monomer or precursors into the pores takes place, see pathway A in Figure 1. If it is a monomer, then the size of the guest is required to be relatively smaller than the pore window aperture of the MOF host; if it is a precursor, this will require additional synthesis steps to obtain the desired guest.^25,26^ In general, a significant advantage of the postsynthesis method is that it helps to preserve the chemical integrity of the guest species because many luminescent guests may not be stable if introduced directly during MOF synthesis stage. This means that postsynthetic confinement helps to avoid guest exposure to the MOF synthesis environment, where harsh conditions such as acidic or basic media, or high temperature and pressure could degrade the luminescent guest molecules. Nevertheless, the postsynthesis method presents the following shortcomings: time-consuming (up to several days), more complicated synthesis steps, and uneven guest distribution due to differential diffusion rates on the internal and external surfaces of MOF crystals.^27^

At present, the commonly used methods to attain postsynthetic LG@MOF systems are liquid impregnation and gas-phase infiltration. The operation of liquid impregnation is straightforward. In principle, the prepared MOF is immersed in a concentrated solution containing the dye guest molecules intended for encapsulation by the MOF host. Through processes, such as ion exchange and diffusion, the luminescent guest will be absorbed or adsorbed by the MOF to form a LG@MOF system. After guest uptake is completed, centrifugation, washing, drying, and other operations are usually performed to remove residual dyes and to collect the resultant LG@MOF crystals. For example, Hu et al.^28^ directly immersed an anionic Zn MOF with 1-D channels [(CH_3_)_2_NH_2_]^+^[Zn_4_(μ_4_-O)(NTB)_2_(NO_2_-bdc)_0.5_]·3DMA; NTB = 4,4′,4″-nitrilotrisbenzoic acid, NO_2_-bdc = 2-nitro-4-benzenedicarboxylic acid] into the cationic rhodamine B (RhB) in a dimethylacetamide (DMA) solvent for 1 h, to yield the RhB@MOF system. Fu et al.^29^ also used this liquid impregnation method, by immersing another Zn MOF [Zn(TIPA)(NO_3_^–^)_2_(H_2_O)]·5H_2_O; TIPA = tri(4-imidazolylphenyl)amine] in an aqueous solution of an anionic dye HPTS (8-hydroxy-1,3,6-pyrenetrisulfonic acid trisodium) for 2 weeks, to attain the dual-emissive HPTS@MOF system.

Compared with liquid impregnation, the operation of gas-phase loading is relatively more complicated as this usually requires specially designed vessels, high temperature, and a low-pressure environment. However, using this method means it is solvent-free, which can avoid the unwanted competition between solvent absorption and guest absorption by the MOF host. For example, Muller et al.^30^ combined the luminescent DXP [*N*,*N*-bis(2,6-dimethylphenyl)-3,4:9,10-perylene tetracarboxylic diimide] guest molecules and a number of different MOFs (MOF-5, MOF-177 and UMCM-1) in a specially designed glass tube under inert gas conditions (Ar). Subsequently, evacuation, sealing, and heating operations (300 °C) were applied and retained under static conditions for several days to yield the DXP@MOF materials. Tu et al.^31^ demonstrated the postsynthetic sublimation approach to encapsulate anthracene (ANT) in gas phase into the pores of ZIF-8. The preactivated ZIF-8 powder and ANT were placed together in a Schlenk tube and evacuated (0.1 mbar), then heated at 120 °C for 3 days. The resultant ANT@ZIF-8 system shows a promising reversible yellow-to-purple photoswitching of its fluorescence.

### 2.2 *In Situ* LG Confinement Methods

LG@MOF systems can also be fabricated by employing the *in situ* nanoconfinement method. This encapsulation approach can simply be described as the guest molecules being combined directly with the MOF’s basic building blocks (i.e., the metal source and organic linkers) in the same vessel prior to formation of MOF structure, see pathway B in Figure 1. Facilitated by host–guest interactions during coordination directed assembly, bulky guest molecules,^32^ especially those with a size larger than the MOF window aperture, could be successfully encapsulated within the MOF pores, or immobilized in the MOF crystal structure to give a “core-shell” type composite. Compared with the postsynthesis confinement method (Section 2.1), the *in situ* approach is relatively simpler with shorter reaction times, and could yield a better result in terms of guest distribution. However, one important factor that must be considered is the stability of the guest species during *in situ* encapsulation process, so that their chemical integrity is not compromised under the synthesis conditions of the MOF host; to this end, typically milder synthetic conditions are used.

**Figure 2:**
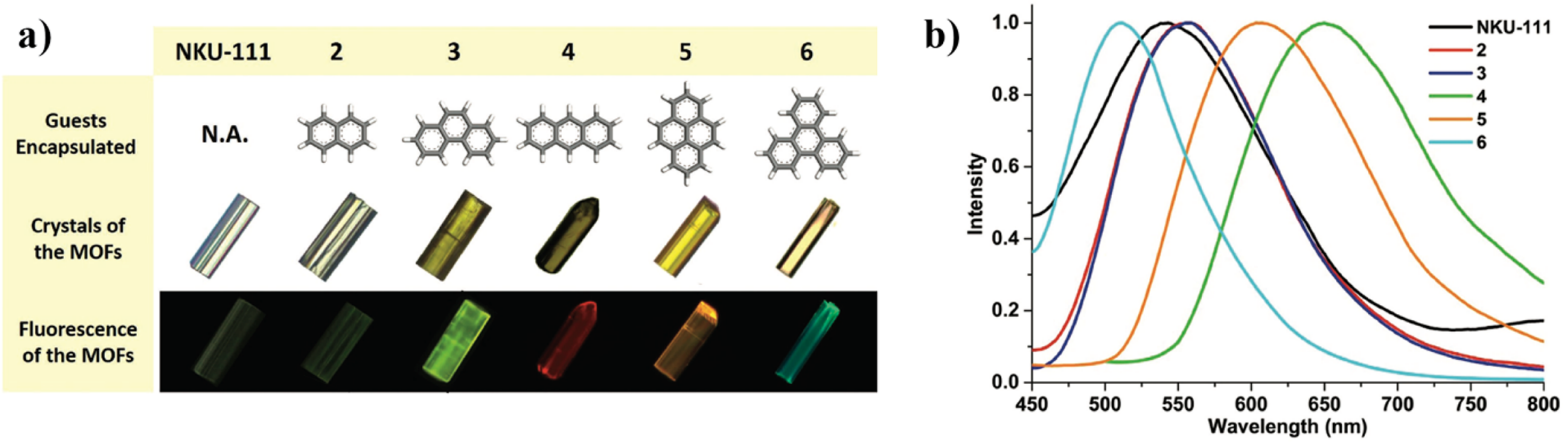
(a) Luminescent guest@NKU-111 series showing the guest-dependent optical and luminescent images of the crystals, where the confined fluorophores comprise naphthalene (2), phenanthrene (3), anthracene (4), pyrene (5), and 9,10-benzophenanthrene (6). (b) Normalized emission spectra of the LG@NKU-111 series determined under an excitation wavelength of 420 nm. Adapted with permission from ref (33). Copyright 2018 John Wiley and Sons.

The simplest and most common way to achieve *in situ* synthesis of LG@MOF is via one-pot reaction. The process directly combines in a reaction mixture the dye guest molecules, metal salts, and organic linkers of the intended MOF, and then allows the coordination directed self-assembly to take its own course to completion. For example, Zhang et al.^33^ used the one-pot method to successfully prepare a series of LG@NKU-111 system with tunable luminescent properties, see Figure 2. It was reported that the host–guest assembly (heated for 1 day at 100 °C) was facilitated by the donor–acceptor interaction forming between the electron-rich fluorescent guests and the electron-deficient host framework. Asadi et al.^34^ also demonstrated a one-pot method performed under room temperature for the encapsulation of polyethylene glycol-capped ZnS quantum dots (PEG-ZnS QD) within the ZIF-67 MOF. To this end, a solution containing the organic linker (2-methylimidazole) and the PEG-ZnS QD were mixed with another solution containing the metal salt (Co(NO_3_)_2_·6H_2_O) and the reaction was stirred for 45 min, yielding the luminescent hybrid material PEG-ZnS QD@ZIF-67.

**Figure 3:**
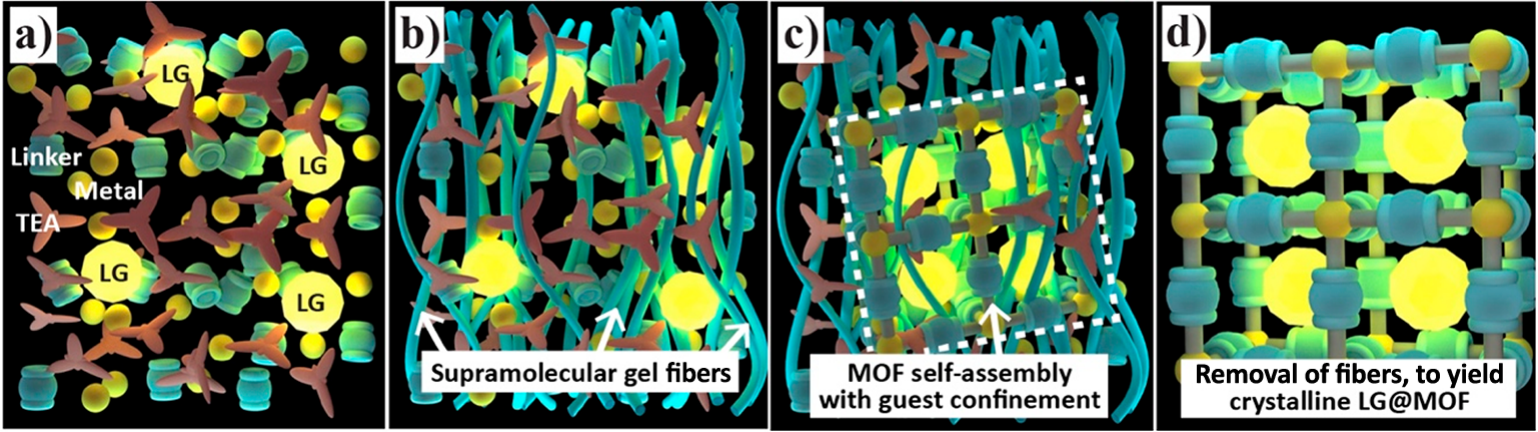
Schematic illustrating the sequential stages of the *in situ* encapsulation process by HCR method to yield LG@MOF. (a) High-concentration reactants are combined with luminescent guests (LG presented as yellow sphere). (b) Formation of a supramolecular gel fiber network facilitating the self-assembly of (c) MOF host confining the luminescent guest molecules. (d) LG@MOF crystals are harvested by disintegrating the scaffolding fibers in a solvent. Adapted from ref (35). Copyright 2017 John Wiley and Sons.

**Figure 4:**
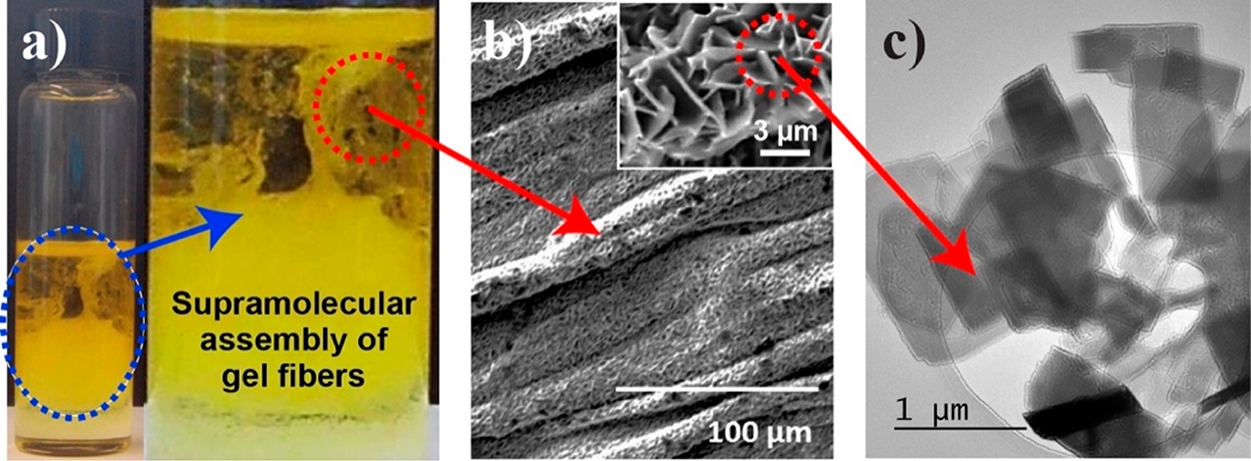
(a) *In situ* synthesis of a LG@MOF material employing the high-concentration reaction (HCR) method, where the fluorescent guest is ZnQ, resulting in the formation of (b) supramolecular gel fibers coexisting with nanosheets of ZnQ@OX-1 (SEM images and inset). (c) TEM micrograph of the nanosheets obtained through downsizing of the 3D framework of the OX-1 MOF. Adapted from ref (35). Copyright 2017 John Wiley and Sons.

More recently, an *in situ* guest encapsulation strategy termed the “high-concentration reaction” (HCR) method has been developed for synthesizing a variety of LG@MOF systems (Figure 3).^35,36^ Briefly, the synthesis by HCR involves the following key steps: (A) first, prepare a metal salt solution containing the metal centers of the intended MOF host; (B) second, prepare another solution of organic linkers combined with fluorescent guests dispersed in a suitable solvent; (C) finally, solutions A+B are combined to initiate the reaction, where a gel-like material coexisting with LG@MOF crystals forms immediately (Figure 4a,b). Resultant LG@MOF crystals can be isolated from the supramolecular gel by washing in an organic solvent. Notably, HCR method produces a faster reaction rate and higher sample yield than the conventional one-pot approach described above. This is generally caused by the use of deprotonation agents, such as triethylamine (TEA), which is typically added to the linker solution to accelerate the deprotonation rate of ligands. For instance, the ZnQ@OX-1^35^ [ZnQ = zinc(II) bis(8-hydroxyquinoline)], Gaq_3_@ZIF-8^37^ [Gaq_3_ = gallium(III) tris(8-hydroxyquinoline)], RhB@ZIF-71^36^ [RhB = rhodamine B] and perylene@ZIF-8^38^ with fluorochromic and optoelectronic properties (Sections 6.1 to 6.4) have been prepared by leveraging the HCR methodology. Because of the effect of downsizing, typically the crystals obtained from this method will be relatively small, giving 2-D morphologies such as nanoplates and nanosheets (Figure 4c). While the characterization of the nanosized crystals is more challenging as they require high-resolution SEM, TEM, or AFM techniques (Section 4), they do offer better processability and improved photoluminescent properties such as a high quantum yield (Φ > 90%) via electrospun nanofibers.^36^

### 2.3 Importance of Sample Washing Step and Role of Guest Concentration

No matter which method is used, it is critical that the obtained LG@MOF samples are washed thoroughly in an appropriate solvent to remove residual guests adhered to the MOF surface because during the synthesis process, some guest molecules will inevitably be adsorbed on the outer surface of the MOF crystals, where these external guests are not protected by the MOF host. Properties of any unprotected fluorophores will be susceptible to degradation by the external environment,^39,40^ which may have an adverse effect on the luminescent properties and photostability of the LG@MOF system as a whole. In general, the obtained LG@MOF material should be washed multiple times (with repeated sonication and centrifuging steps) until the mother liquor becomes clear and nonemissive. For example, with sufficient washing cycles, the ZnQ@OX-1 material^35^ can exhibit interesting sensing properties for acetone vapor, but it will only emit a constant blue light without any sensing behavior if being washed once or twice. However, too much washing could risk removal of weakly confined guests from the pore, leading to a deterioration of fluorescent properties, especially when employing channel-type MOFs as a host.^28,41^ Hitherto, the cases of sample failure or poor batch-to-batch sample reproducibility that may be linked to either too little or too excessive washing of resultant guest-encapsulated samples are rarely reported in the literature; this synthetic issue clearly deserves more attention to help the community improve the overall reproducibility of reported LG@MOF systems.

Another critical aspect that will have a direct impact on the luminescent properties of the LG@MOF material is the guest concentration within the MOF host. Two key factors must be considered when synthesizing a LG@MOF system, to maximize the luminescence quantum yield of the material and to possibly tune the color emission. On the one hand, if the concentration of the guest is too high, most probably the dyes will aggregate, and as a consequence, the fluorescence quantum yield (Φ) will generally decrease by ACQ (although in some cases Φ may increase because the dyes experience aggregation-induced emission, AIE), and then the emission will generally be red-shifted due to the formation of J-aggregates (see Section 5.4). On the other hand, if the concentration is too low, the dyes will be incorporated mainly as isolated monomers within the pores of the MOF. However, even if the luminescence quantum yield of the composite material may be high (as the luminescence quantum yield is a ratio between the emitted and absorbed photons), the total fluorescence intensity of the material could be very low, as the amount of fluorescent guest present is relatively low per unit mass (see Section 4.3). Hence, it is vital to be able to precisely adjust the concentration of the LG within the MOF, in a way that the concentration is high enough to yield a strong luminescent material, but not excessively concentrated to prevent the formation of a large population of unwanted aggregates.

## 3 Types of Luminescent Guest for Confinement in MOF

**Figure 5:**
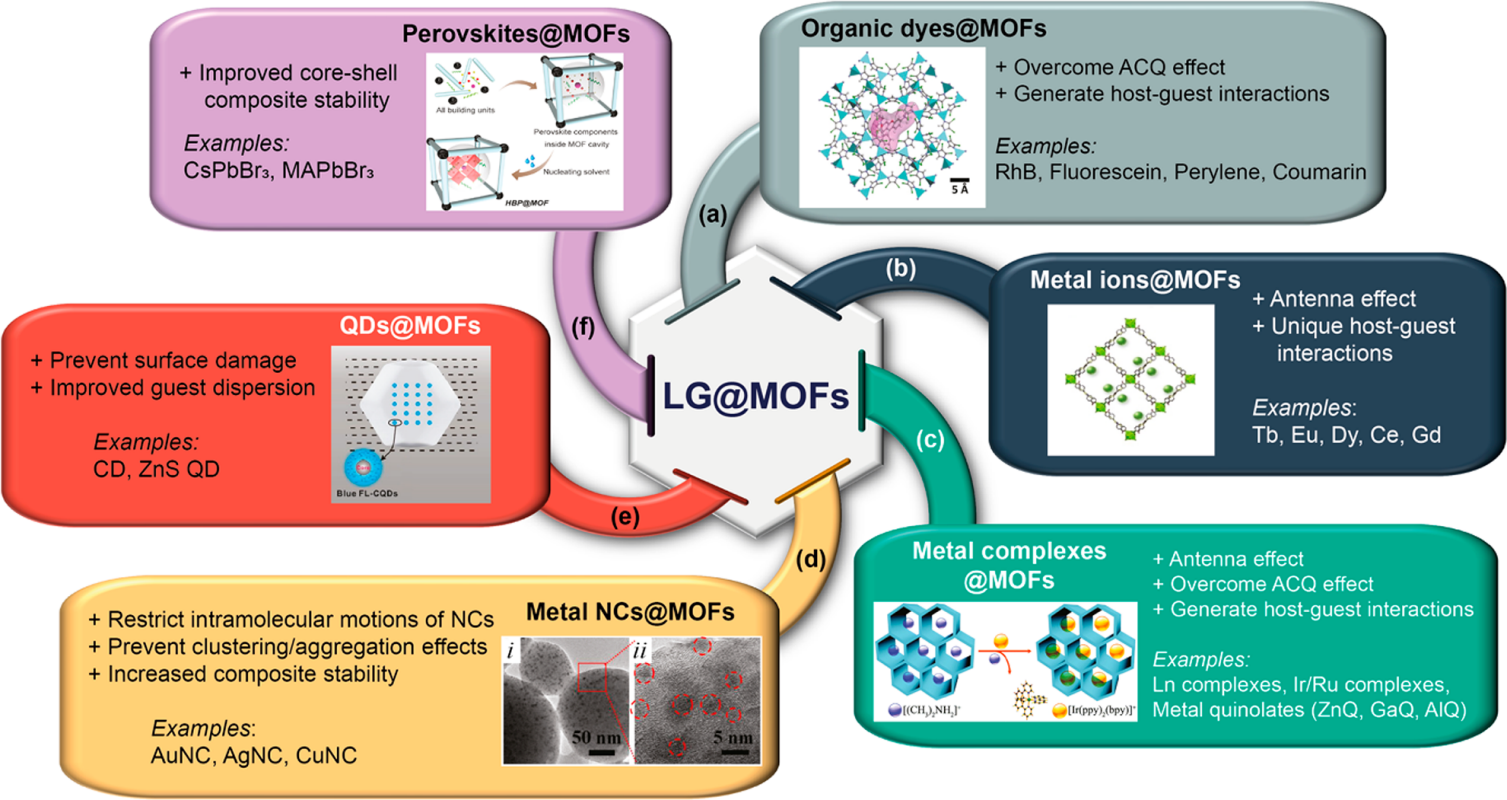
Major types of luminescent guest (LG) that have been encapsulated inside the MOF host to yield the LG@MOF composite systems with specific advantages summarized. (a) Organic dyes, figure inset adapted from ref (43). Copyright 2020 American Chemical Society. (b) Metal ions, figure inset adapted with permission from ref (52). Copyright 2019 Elsevier. (c) Metal complexes, figure inset adapted from ref (54). Copyright 2017 Royal Society of Chemistry. (d) Metal nanoclusters (NCs), figure inset adapted from ref (63). Copyright 2018 American Chemical Society. (e) Quantum dots (QDs), figure inset adapted from ref (68). Copyright 2014 American Chemical Society. (f) Hybrid perovskites, figure inset adapted from ref (26). Copyright 2019 American Chemical Society. Panels d and e are representative examples of core–shell type composites.

Because of the high tunability of MOF structures, it is unsurprising that the types of luminescent guests are also equally diverse. In general, for most guest types, MOFs can help to improve and enhance the luminescent performance of the encapsulated guests, mainly by: (1) reducing ACQ effect and allowing many guests that can only fluoresce in solution to achieve luminescence in the solid-state, (2) imposing a caging effect on the guests, thereby reducing the nonradiative decay of the guests and increasing their lifetime (*τ*) and quantum yield (Φ), and (3) there are many MOFs with good thermal stability and water stability such as ZIF-8, UiO-66, and ZIF-71. Using these stable MOF hosts as a “shield” can protect and improve the stability of the confined guests and broaden their scope of application (Section 6). At present, the main types of luminescent guest commonly studied in the context of LG@MOF are summarized in Figure 5, which encompass the organic dyes, metal ions/complexes/nanoclusters, quantum dots, and inorganic–organic (hybrid) perovskites. The following sections will present some topical examples of luminescent guests being confined in MOFs.

### 3.1 Organic Dyes@MOF

Organic luminescent dyes are a widely used luminescent materials, the luminescence of which stems from the π–π conjugation of their chromophoric groups. This kind of material has a relatively broad emission spectrum, may have a high quantum yield, and some of which have luminescence tunability, but most face the limitation of ACQ effect and can only emit in solution. Importantly, the organic dyes@MOF systems can effectively overcome the ACQ effect and achieve a solid-state emission. For example, the RhB@bio-MOF-1 reported by Chen et al.^42^ has helped the confined RhB guests to overcome the ACQ effect and thus yield luminescence in the solid state.

Apart from addressing the ACQ effect, the organic dye@MOF systems may also enhance or widen the luminescence tunability of the composite, which will significantly advance its sensing, display, and light emission applications (Section 6). For instance, the extensively studied RhB@MOF systems broaden the solvatochromism of RhB itself by allowing the RhB@ZIF-71 system to achieve different luminescent response even in nonpolar solvents (e.g., toluene, hexane, cyclohexane), whereas the pure RhB molecule has poor solubility or is insoluble, hence, precluding its luminescence by ACQ.^43^ Moreover, the encapsulation of the organic dye guests in the MOF pores can give rise to novel mechanochromic materials due to changes in the interchromophoric distances with an applied pressure, which may enhance, quench, or shift the emission behavior under stress. Exemplars of mechanochromic LG@MOF systems are presented in Section 6.3. For a comprehensive list of commonly used small-molecule fluorescent dyes (e.g., fluoresceins, rhodamines, coumarins, naphthalimides, cyanines, pyrene), with descriptions of their structures and photophysical properties, the reader may consult a number of specific reviews dedicated to this subject matter.^44,45^

### 3.2 Metal Ions@MOF

The metal ions used in the LG@MOF systems are mainly lanthanide (Ln) ions.^46^ The Ln ions display sharp characteristic emission peaks, they are generally stable in the ambient environment and their luminescence is governed by the forbidden f–f transitions.^47^ Using the periodic structure of MOF to confine Ln ions means this will enable a larger number of photons to be emitted per unit volume.^48^ Moreover, the organic linker of MOF is highly tunable, which facilities MOF interaction with the Ln ions, promoting energy transfer or establish the antenna effect for enhancing the luminescence efficiency of Ln ions. Together, these factors may help to raise the quantum yield of the confined systems to about 14–30%.^49−51^ Since the energy transfer between the MOF and Ln ions is sensitive to the external environmental stimuli, the metal ions@MOF system can attain an optical sensing response. For example, the Tb@Zn-MOF system reported by Ji et al.^52^ exhibits a noticeable energy transfer/antenna effect between the linker and the terbium (Tb) ions. This kind of host–guest interaction was exploited to prepare a turn-on type sensor that responds to aspartic acid, see further exemplars in Section 6.1.

### 3.3 Metal Complexes@MOF

Metal complexes themselves are also diverse, but different metal complexes face different challenges to reach practical applications. Confinement of metal complexes in a MOF host can further tailor their luminescent properties, expand applications, and even give several metal complexes new sensing capabilities.

Rare-earth Ln complexes are extensively studied in the context of luminescent MOFs.^11,12,53^ The advantages and disadvantages of the Ln complex are akin to those faced by the Ln ions (Section 3.2), employing MOF for the confinement of Ln complex may help to overcome some outstanding problems. However, most of the rare earth elements are toxic, costly and scarce. Other metal complexes with good luminescent performance have become more attractive, such as iridium (Ir)- and ruthenium (Ru)-based complexes. Ir and Ru complexes possess high luminescence efficiency and tunable emission, but they are also affected by the ACQ effect to a certain extent. Some researchers have successfully applied Ir and Ru complexes to construct the metal complex@MOF systems, which suppress the ACQ effect and produce a white light emission where the quantum yield was found to be Φ ≈ 15–20%.^54,55^

Another promising family of electroluminescent nonrare-earth metal complexes being investigated for confinement in the LG@MOF system is the metal quinolate series, denoted as Mq_n_ (e.g., Alq_3_, Gaq_3_, Inq_3_, Scq_3_, Znq_2_; q = 8-hydroxyquinoline).^56^ The metal quinolates have excellent luminescent performance with good stability, and are relatively low cost. The construction of Mq_n_@MOF systems can make full use of the advantages offered by this kind of complexes, as demonstrated by a recent example where Gaq_3_@ZIF-8 is incorporated in an electroluminescent device (LED) as an electroactive layer (further details in Section 6.7).^37^ Interestingly, the host–guest interaction between the Mq_n_ and MOF can generate unique sensing properties. For example, ZnQ@OX-1 system incorporating a zinc(II) bis(8-hydroxyquinoline) complex (ZnQ or Znq_2_) shows a fast and reversible fluorescence sensing response toward acetone vapor.^35^

### 3.4 Metal Nanoclusters@MOF

Luminescent metal nanoclusters (NCs: e.g., gold, silver, copper) are composed of several to tens of metal atoms, translating to a nominal size of ca. 1–2 nm.^57^ Because their size is comparable to the Fermi wavelength of electrons, metal NCs receive a strong quantum confinement effect, resulting in unique luminescent properties. In general, they have a relatively broad emission, and because of their nontoxicity, metal NCs are attractive for use in the biomedical field (Section 6.5). Nevertheless, compared with other luminescent materials, such as organic dyes, the quantum yield of metal NCs is low,^58−61^ which dramatically limits their further development.

Some studies^59,60^ have suggested that restricting intramolecular motions of metal NCs can improve the quantum yield. That can be realized by confining metal NCs within the MOF scaffold to yield a core–shell composite. Importantly, the immobilization of NCs in the MOF host prevents clustering and aggregation effects that will degrade luminescent performance.^62^ For example, Gao et al.^63^ used the system of AuNCs@ZIF-8 to boost the quantum yield of Au NCs by approximately 4.5 times, where Φ was reported to rise from 7.6% to 33.6% after their immobilization in the ZIF-8 host. Furthermore, MOF crystals can also play a protective role for some unstable metal NCs, such as Cu,^64^ which is susceptible to oxidation (Section 4.6). It has been reported that when confined within the scaffolding of the MOF host, the stability of the Cu nanoclusters can be substantially prolonged from 3 days to 3 months. Given its promise, there is an increasing number of research focusing on the development of metal NCs@MOF systems. On this note, while silver NCs@zeolite systems show many intriguing luminescent properties,^62^ there is still very limited work exploring the use of MOFs to afford nanoconfinement of silver NCs.^65^

### 3.5 Quantum Dots@MOF

Quantum dots (QDs) are defined as semiconductor nanocrystals with a diameter of about 2–10 nm. Like the metal nanoclusters mentioned above, QDs are also subject to a strong quantum confinement effect due to their small size, leading to a good optical performance, including a broad excitation range, high quantum yield, high extinction coefficients, and tunable luminescence.^66^ Generally, although some materials are not semiconductors, they are often discussed as QDs, especially carbon dots (CDs), also known as carbon quantum dots. In addition to having excellent luminescent properties, CDs also possess chemical inertness and better biocompatibility, which can be deployed in biomedicine. However, QDs face certain challenges in terms of their application. One limitation is that the luminescent properties of QDs, particularly the quantum yield, is greatly affected by surface defects. Thus, some additional protection is needed to prevent surface damage. Another drawback is that it is difficult to achieve a good dispersion of QDs during use, which may negatively impact their luminescence.

MOFs are proposed to be an effective solution to these problems faced by QDs. For example, Wang et al.^67^ developed a CD@ZIF-8 luminescent system and show that the use of MOF solves the problem of CD quenching by preventing the formation of aggregates. Furthermore, such QDs@MOF systems can also bestow novel sensing properties. For instance, BPEI-CDs [branched poly(ethylenimine)-capped carbon quantum dots]@ZIF-8 system reported by Lin et al.^68^ exhibits Cu^2+^ sensing properties, in which the role of the nanoporous MOF host was also to help accumulate the target analytes, thereby enhancing its detection sensitivity toward Cu^2+^. Further examples will be discussed in Section 6.4 focusing on cations and anions sensing applications.

### 3.6 Perovskites@MOF

Perovskites have attracted more and more attention because of their low cost, high quantum yield, and tunable optical properties. However, this class of framework material has a well-known problem: poor stability. Different mechanisms, ascribed to a range of intrinsic factors (e.g., chemical structure, defects, phase and thermodynamic stability) and extrinsic factors (e.g., moisture, thermomechanical, light, oxidation induced) could cause the degradation of hybrid perovskites and impact their long-term performance.^69^

Some relatively stable MOFs, such as the ZIF and UiO series, have been employed in combination with perovskites in an attempt to develop stable perovskite@MOF systems with improved chemical stability and photoluminescence. For example, Zhang et al.^70^ successfully improved the stability of halide perovskites through encapsulation in UiO-67, resulting in a core–shell type composite. The luminescent properties of the prepared CsPbBr_3_@UiO-67 was reported to remain unchanged for 30 days under ambient atmospheric conditions. Likewise, Mollick et al.^26^ have reported a series of MAPbBr_3_@ZIF-8 with tunable photoluminescent properties, where the protection offered by MOF host has increased chemical stability of the resultant LG@MOF composites. In terms of the potential technological applications of perovskite@MOF systems, the reader may refer to Section 6.2 on temperature sensing and Section 6.6 on luminescent anticounterfeiting inks.

Section 3 gives an overview of the different types of luminescent guests (LG) suitable for confinement within a MOF host, where each category clearly has its advantages and constraints. The representative examples of the luminescent guests summarized in this section will be further elucidated in the context of their applications as LG@MOF systems in Section 6. Next, in Section 4, we will expose the readers to state-of-the-art characterization tools for probing guest inclusion within MOFs, with the intention to demonstrate how these tools are best employed in a complementary fashion while emphasizing the pros and cons of each technique.

## 4 Characterization Methods for LG@MOF, Understanding Their Capabilities and Limitations

Regardless of the methodology followed for the fabrication of the LG@MOF composites, it is highly plausible that the guests can be adsorbed onto the surface instead of, or, in addition to being encapsulated into the pores or entrapped in the crystals (core–shell type). While different techniques provide information about the structure, chemical composition, and physicochemical properties; proving the encapsulation of the guest in the MOF host is certainly not a trivial task and this usually requires the use of complementary techniques. In this section, we will discuss and critically assess the advantages and limitations of some of the most employed techniques for the characterization of LG@MOF systems.

### 4.1 X-ray Diffraction Techniques

**Figure 6:**
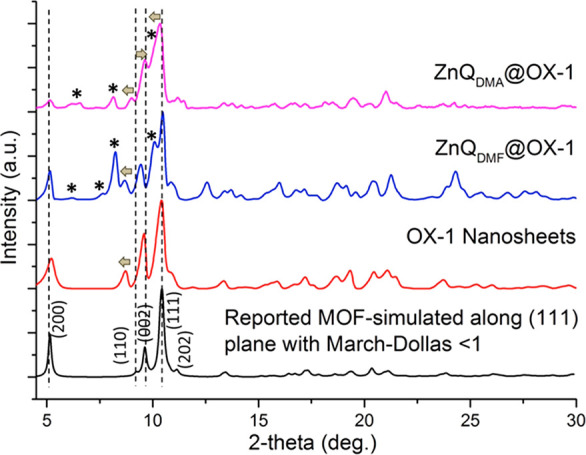
PXRD patterns of the OX-1 nanosheets, with and without confinement of the ZnQ guest molecules, compared with the simulated diffraction pattern of the pristine OX-1 MOF. Asterisks mark the peak positions of the ZnQ luminescent guest. Adapted from ref (35). Copyright 2017 John Wiley and Sons.

Powder X-ray diffraction (PXRD) is a rapid and nondestructive technique used for the structural characterization of powders and microcrystalline materials. For this reason, it has become a routine technique for the determination of the crystalline structure of MOFs and especially of LG@MOF composites, where the inclusion of guests can induce a distortion in the long-range periodicity of the MOF crystals. Generally, the crystalline structure of MOFs remains unaltered after the encapsulation of the photoactive compounds,^32,37,71−83^ mainly because a series of factors such as the MOF rigidity, the guest size, the low loading percentage, or the adsorption of the guest onto the MOF surface instead of being encapsulated. However, there are a few interesting examples in which guest encapsulation produces structural modifications in the network.^35,84^ For instance, the flexible [Zn(ndc)(o-phen)]·DMF_n_ MOF exhibits notable changes in its PXRD pattern upon desolvation of DMF solvent molecules.^84^ The changes in the MOF structure were determined through *in situ* PXRD measurements coupled with CO_2_ adsorption, where it was demonstrated that an increase in the cell volume with CO_2_ uptake (from 2143 to 2963 Å), reflecting an expansion of the framework. This observation could be extrapolated to the inclusion of four different aromatic amine compounds, which gave rise to PXRD patterns quite distinct from the one of the pristine material alone, indicating a structural reorganization after guest encapsulation.^84^ Similar phenomenon was observed for the Zn-based OX-1 ((H_2_NEt_2_)_2_[Zn_3_bdc_4_]·3DEF) MOF, whose Bragg diffraction peaks were shifted after the encapsulation of the luminescent ZnQ metal complex, corresponding to an expansion of the unit cell parameters (Figure 6).^35^

**Figure 7:**
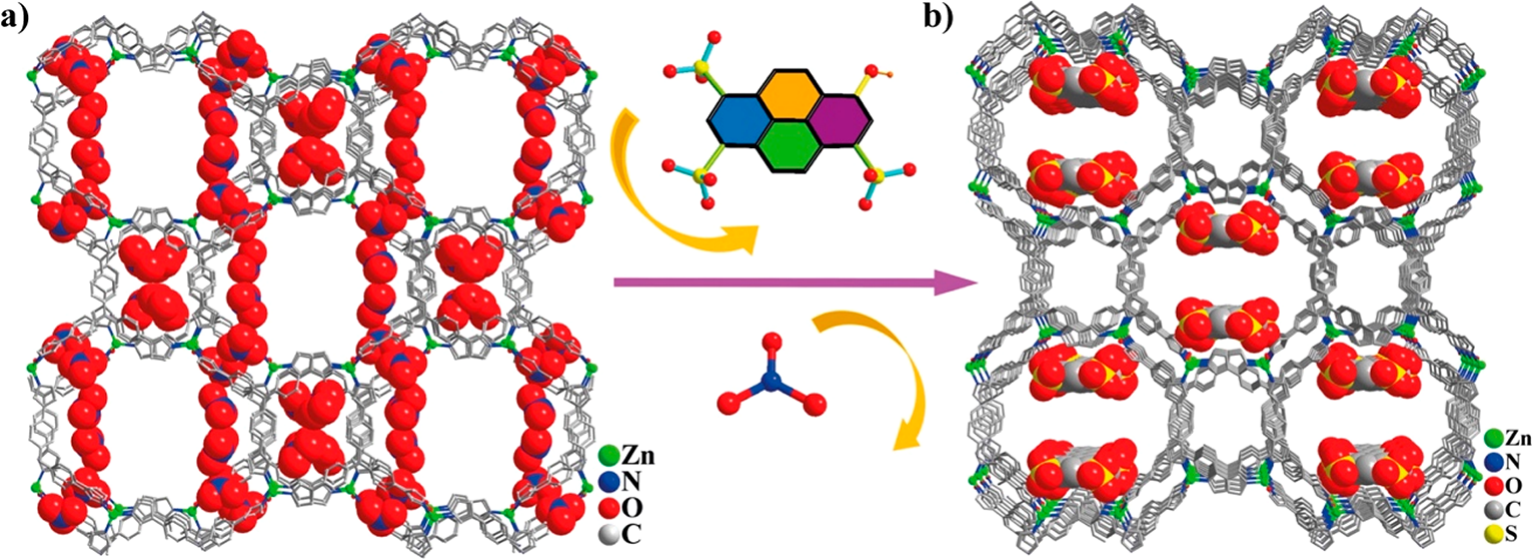
Representation of the crystalline structure of (a) the 3D framework of FIR-53 MOF and (b) the structure of the MOF upon the encapsulation of the luminescent guest (SG7) into the channels, to yield SG7@FIR-53. Adapted with permission from ref (90). Copyright 2018 American Chemical Society.

Although PXRD is a convenient tool for structural characterization, it is not the most suitable technique for the detection of confined guests. There is usually a lack of signal associated with the active guests owing to two main reasons: (i) to detect Bragg diffraction peaks, the guests should have long-range periodicity, however, they are more likely to be randomly distributed within the MOF host; and (ii) the weight % of confined guests in comparison to the MOF host is typically low,^85−88^ and it is well-known that PXRD is not a very sensitive technique (typical detection limit is of the order of 5–10 wt %).^89^ On the contrary, single-crystal (SC) XRD enables a more precise identification of the presence and position of confined guests in the MOF structure. For example, the encapsulation of the ionic form of the luminescent solvent green 7 (SG7^3–^) as a counterion in the large channels of FIR-53 (Zn_2_(TIPA)_2_(OH^–^)](NO_3_^–^)(SG7)_2/3_·5H_2_O) MOF was confirmed by SCXRD (Figure 7), being the first example of the inclusion of a π-conjugated organic small molecule via a single-crystal to single-crystal transformation.^90^ However, SCXRD presents an inherent limitation related to the sample preparation, which has to be a single crystal of a considerable size and as defect-free as possible. On the one hand, this is of course, not a minor task, and on the other hand, it will hinder the applicability of those materials, whose synthesis must be easily scaled up for practical implementations (Section 6).

### 4.2 Steady-State UV–Visible and Fluorescence Spectroscopy

Steady-state UV–visible absorption/reflectance and fluorescence spectroscopic techniques are essential for characterizing the optical properties of the ground and electronically excited states of LG@MOF systems. They will also allow the quantification of the interaction of the fluorescent guests with the MOF hosts. For instance, the loading percentage of guests interacting with a specific MOF can be estimated by measuring the optical density at the maximum intensity of the guest solution before and after its interaction with the MOF. Furthermore, it is also possible to calculate the mass of the loaded dye by knowing the molar extinction coefficient of the guest, which can be estimated through UV–visible absorption by applying the Beer–Lambert formula.^91^ The percentage of guest loading can be calculated through the following equation:
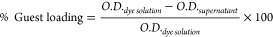
where *O.D.* stands for the optical density, the *dye solution* corresponds to the initial solution before its interaction with the MOF, and the *supernatant* is the solution after being loaded in the MOF. For a reliable characterization of the % guest loading, the key facts below must be considered: (i) obviously, the *O.D.* of the supernatant must be always lower than that of the initial dye solution, as a certain amount of dyes will be interacting with the MOF. (ii) A key factor is that the volume of the dye solution and the supernatant must be exactly the same as the *O.D.* is directly proportional to the concentration. If, for example, the solvent of any of these solutions is evaporated during the process, the *O.D.* values will be higher because the concentration of this solution will also rise. (iii) Finally, as described in Section 2.3, it is important to wash the sample properly and one may consider that in the second or even in the third supernatant the amount of dye might not be negligible. In those cases, it is important to determine the *O.D.* of these solutions and add the values to the total *O.D.* of the supernatant. If all these points are carefully met, UV–visible absorption spectroscopy, an affordable, rapid and routine technique becomes a powerful instrument to estimate the amount of not only luminescent but many different types of guest entrapped in different host materials.^92^ Indeed, many reports have assessed the loading percentage of fluorescent guests in MOFs by employing this methodology.^73,85,93,94^

**Figure 8:**
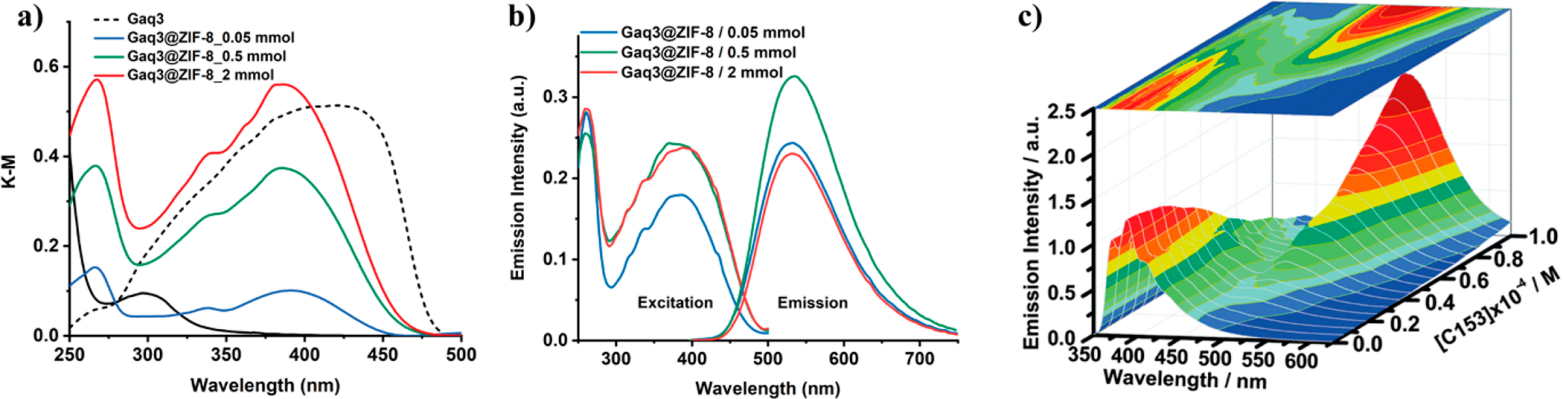
(a) Diffuse reflectance spectra transformed to Kubelka–Munk (K–M) functions, (b) excitation and emission spectra of three samples of Gaq_3_@ZIF-8 with an increasing amount of guest confined in the ZIF-8 host. Adapted from ref (37). Copyright 2020 John Wiley and Sons. (c) Fluorescence spectra of C153@Zr-NDC in a diethyl ether suspension. Increasing concentrations of C153 dye produces an increase in intensity of the 525 nm band (emission of C153) but a decrease of the 400 nm band (emission of Zr-NDC MOF). Adapted with permission from ref (85). Copyright 2015 Royal Society of Chemistry.

UV–visible absorption and fluorescence spectroscopies are also routinely employed for the qualitative characterization of the fluorescent guests in MOFs. As the fluorescent guests usually exhibit characteristic absorption and emission spectra, the detection of those signals in the LG@MOF system is frequently used as a proof for the presence of the luminescent guest.^35,54,75,78,85,88^ For instance, the confinement of Gaq_3_ in the pores of ZIF-8 MOF was confirmed by the absorption, excitation and emission spectra, corresponding to the characteristic bands of the fluorescent guest (Figure 8a,b).^37^ Additionally, increasing the amounts of loaded guest will induce an increase in the absorption intensity, and generally accompanied by a rise in the emission intensity of the LG@MOF composite as well (however, it can sometimes produce a decrease of the emission intensity owing to undesired physical effects, such as inner filtering), and these changes in the signal intensity have been correlated with the amount of fluorescent guest in the MOF host (Figure 8c).^35,54,75,78,85,88^

Another important parameter is the luminescence quantum yield (Φ) of the LG@MOF composites, which is defined as the ratio of the number of photons emitted to the number of photons absorbed. The Φ value can be measured by using an integrating sphere coupled to a fluorometer and it will be of great importance not only to have an approximate idea of the ability to emit light of the LG@MOF system, but also it is an important correction factor for determination of other parameters, such as the Förster energy transfer efficiency (see Section 5.1) and fluorescence quenching.^95^ Both techniques, and especially the fluorescence spectroscopy, are therefore indispensable for the characterization of such excited-states processes and for the further integration of these materials in advanced technologies such as solid-state lighting or optical sensors (see Section 6). However, despite the great advantages evidenced above, these techniques will provide information on the presence of guests, but generally not a confirmation of their encapsulation within the MOF. The limitation lies in the fact that the signal will be equally detected no matter if the guests are on the surface, encapsulated in the pores, entrapped in the crystals, or a combination of some, or all, of the above locations. It is worth emphasizing that the fluorescence measurements can be complimented with time-resolved emission spectroscopy like time-correlated single photon counting (TCSPC) or fs-up-conversion techniques (see Section 5.4). Indeed, these time-resolved techniques are powerful tools to further unravel excited-state phenomena such as energy-, charge- or proton-transfer mechanisms as well as host–guest interactions.

### 4.3 Nuclear Magnetic Resonance (NMR) Spectroscopy

**Figure 9:**
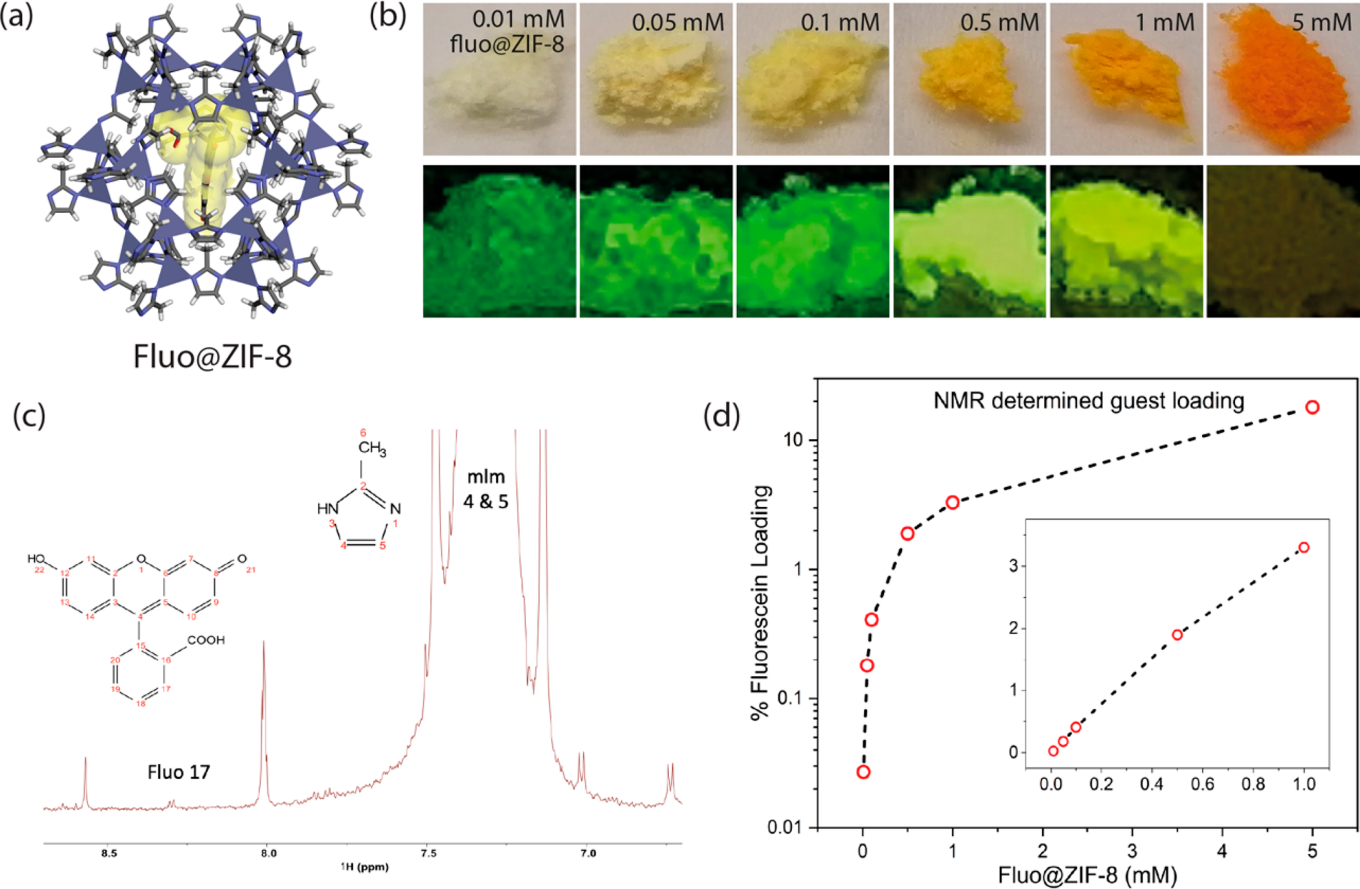
(a) Schematic representation of a fluorescein guest molecule encapsulated in the pore of a ZIF-8 sodalite cage to yield the fluorescein@ZIF-8 composite system. The purple tetrahedra are ZnN_4_. (b) Fluorescein@ZIF-8 samples with fluorescein guest concentrations of 0.01 mM to 5 mM viewed under natural light (top row) and their luminescence excited under a 365 nm UV lamp (bottom row). (c) Solution ^1^H NMR spectrum of the 0.01 mM fluorescein@ZIF-8 sample, where the guest/host peaks used for integration are indicated as Fluo and mIm, respectively. From this spectrum, the guest loading calculated is 1 fluorescein for every ∼3704 cages. (d) Fluorescein loading determined by NMR spectroscopy. The inset shows the loading amount plotted on a linear scale. Dashed lines are guide for the eye. Adapted from ref (83). Copyright 2021 American Chemical Society.

Another approach to establish the amount of luminescent guest confined in the MOFs is by digesting the LG@MOF composite (using acids or other solvents) and recording the solution ^1^H NMR spectra.^31,77,84,96−98^ The molar ratio between the organic linker and the luminescent dye can be attained through the integral area of the ^1^H NMR peaks. However, it is surprisingly frequent to find that in some reports there is a lack of information on how the weight percentage or the number of guest molecules were calculated. To cite a couple of examples, it was described that in the systems: BI@Pb_2_(TCPP)·4DMF (BI = benzylidene imidazolinone) and C460@Eu_*x*_Tb_*y*_BPT (C460 = Coumarin 460) there was one BI guest molecule per two TCPP^4–^ units and 1.02 wt % of C460 encapsulated in each MOF, without any additional information given on how the values were precisely determined.^77,96^ On the other hand, a more detailed study clearly explains the number of anthracene (ANT) molecules encapsulated in the cages of the ZIF-8. First, the authors calculated the molar ratio between the organic linker (mIm) and the guest based on the integral area which was ANT/mIm = 1/3.37. Then, knowing that 1 unit cell of ZIF-8 contains 2 cages with 24 mIm ligands, the loading amount was obtained by multiplying the ANT/mIm ratio by 12. In this example, the loading amount was 3.6 anthracene molecules per cage.^31^ In a very recent example, Tao et al. estimated the amount of fluorescein (Fluo) dye encapsulated in the pores of the ZIF-8 MOF (fluorescein@ZIF-8 system, see Figure 9a-b) by adopting the solution ^1^H NMR methodology elucidated above, and it was found that the increasing amount of fluorescein introduced during the synthesis process does correlate with the guest occupancy of the pores (from 1/3703 to 1/6 fluorescein/cages), see Figure 9c and d.^83^

However, and similarly to UV–vis spectroscopy, this methodology has some limitations. First, even though the NMR technique is nondestructive by itself, the sample preparation step involving the digestion of the material turns it into a destructive methodology. Second and most important, this approach could determine the amount of guest but usually not revealing its location in the MOF, hence the use of complementary techniques will be required. Indeed, in the above case,^31^ a combination of N_2_ adsorption, ^1^H NMR and thermogravimetric analyses (TGA) were required to further corroborate that the anthracene molecules were effectively confined into the cages of ZIF-8.^31^ Despite this, in some very specific cases, ^1^H NMR could reveal information on the guest location through indirect measurements. For example, the MOF Al-DBA (DBA = 9,10-dibenzoate anthracene) contains capping acetates on the outer surface which exhibit a characteristic signal at ∼1.94 ppm in the ^1^H NMR spectra.^97^ After treating this MOF with the luminescent dye RhB, the acetate signal disappears, indicating that RhB is replacing the capping agent in the outer surface of the MOF.^97^

### 4.4 Elemental Analysis

Elemental analysis (EA) is usually known as a technique that permits the qualitative identification and quantitative determination of elements such as C, N, H, and X (where X = halogen, sulfur) of solid or fluid materials. EA is therefore a well-recognized technique to unveil the composition and purity of newly synthesized compounds, especially of organic systems. Moreover, in the last decades, EA is not only an outstanding tool to characterize the composition and purity of MOFs,^76,93,98−100^ but in some cases it also serves to identify or quantify different fluorescent guest compounds interacting with a MOF.^101−103^

For example, the presence of a diarylethene derivative chromophore in the ZJU-88 MOF was confirmed by the detection of 0.84% of sulfur element in the EA of the Guest@MOF composite.^101^ Another example analyzed the amount of N in a Ln@HPU-14 system, observing a decrease from 2.28% to 1.81% after the cation exchange process, which suggests that the (CH_3_)_2_NH^+^ cation was replaced by the fluorescent Ln^3+^ ions, reflecting that indirect EA measurements are also suitable for identifying the presence of guests in MOFs.^104^ On the other hand, it is also possible to quantify the amount of guests within MOFs, like in the case of the 4-(dicyanomethylene)-2-methyl-6-(4-dimethylaminostyryl)-4H-pyran (DCM) fluorophore loaded into two different MOFs (stilbene-MOF and IRMOF-8), where it was calculated to be 5.5% and 8.0%, and the corresponding molecular formulas were expressed as Zn_4_O(C_12_H_6_O_4_)_3_(C_19_H_16_N_3_O)_0.18_ and Zn_4_O(C_16_H_10_O_4_)_3_(C_19_H_16_N_3_O)_0.31_, respectively.^102^

Similarly, in another study, the amount of DASP^+^ ((4-p-(dimethylamino)styryl)-1-methylpyridinium) incorporated in the bio-MOF-100 was calculated using the EA obtained from 4 different batches of DASP^+^@bio-MOF-100 composite. In this case, the authors provide a detailed description of the calculation to establish the DASP^+^ concentration. First, they assumed that the number of replaced cations is *n*, and thus, the chemical formulas for the host@guest compound was Zn_8_(C_5_H_4_N_5_)_4_(C_14_H_8_O_4_)_6_O_2_·(4-*n*)Me_2_NH_2_·*n* (C_16_H_19_N_2_), where *n* was determined by EA of C, H, and N atoms. The authors then used the following equation to calculate the concentration of DASP^+^ in bio-MOF-100: *C*_dye@MOFs_ = *nZ*/(*N*_A_*V*), where *Z* is the number of formula units per cell (48), *V* is the cell volume (330, 226 Å^3^) and *N*_A_ is the Avogadro’s number. Applying this relationship, the concentration of DASP^+^ for the 4 different batches was calculated to be 0.03, 0.06, 0.30, and 0.44 mol/L.^103^

**Figure 10:**
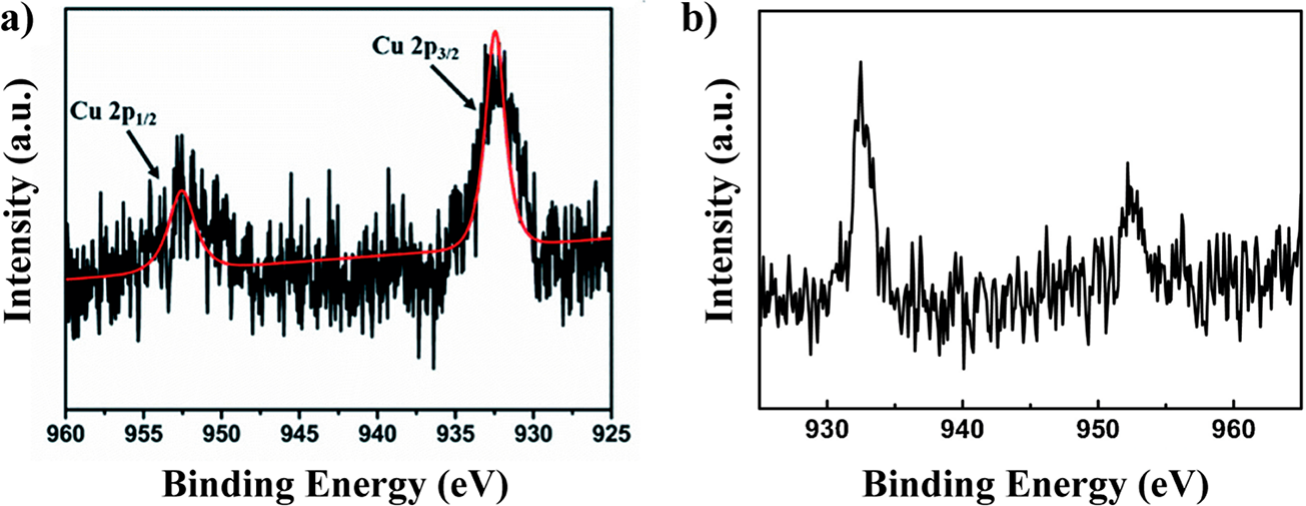
XPS spectrum of (a) Cu NCs@GSH/MOF-5 [GSH = glutathione]. Adapted with permission from ref (64). Copyright 2018 Royal Society of Chemistry. (b) CuNCs-Al^3+^/ZIF-90 composites in the Cu 2p region. Adapted with permission from ref (105). Copyright 2020 Elsevier.

In addition to the conventional elemental analyzer technique, in which the CHNS analysis is accomplished by a combustion of the sample, there exist other techniques capable of detecting and quantifying different elements such as X-ray photoelectron spectroscopy (XPS), X-ray fluorescence (XRF), and inductively coupled plasma optical emission spectroscopy (ICP-OES). All these techniques have also been applied to recognize, and in some cases to quantify the elemental composition of different Guest@MOF materials.^64,105−109^ For instance, the presence of luminescent Cu nanoclusters (NCs) in ZIF-90 or MOF-5 has been detected by means of XPS, where the spectrum of the materials showed the peaks at 932 and 952 eV attributed to Cu_2p3/2_ and Cu_2p1/2_, which are the characteristic peaks of Cu^0^ (Figure 10).^64,105^ Alternatively, another study employed XRF to calculate the amount of Ln^3+^ ions incorporated in two defect-engineered MOFs (UiO-66-AB and MIL-53(Al)-AB), by estimating the ratio between the metal ions of the MOF and the Ln ones, this being 1/1 (Zr/Ln) in the case of UiO-66-AB and 0.5/1 (Al/Ln) for the MIL-53(Al)-AB system.^107^ Other works reported on the combination of ICP-OES and conventional EA techniques for characterizing the chemical composition of different Ln@bio-MOF-1 composites.^108,109^ Indeed, from these experiments they could extract the molar ratios of Zn^2+^:Tb^3+^:Eu^3+^:C:H:N for three Ln@bio-MOF-1 materials containing different amounts of Ln ions, which were found to be 1:0.903:0.098:16.20:19.22:3.63 for Tb_0.9_Eu_0.1_@bio-MOF-1, 1:0.987:0.011:16.14:19.11:3.60 for Tb_0.99_Eu_0.01_@bio-MOF-1, and 1:0.9993:0.0008:16.31:19.15:3.65 for Tb_0.999_Eu_0.001_@bio-MOF-1.^109^

Despite the ability to detect and quantify different elements and therefore to confirm the presence of different fluorescent guests in MOF materials, the EA techniques described herein share the same limitation to the other techniques described above, and it is the inability to pinpoint the precise location of the guests in the MOF hosts.

### 4.5 Vibrational Spectroscopy (IR, Raman)

**Figure 11:**
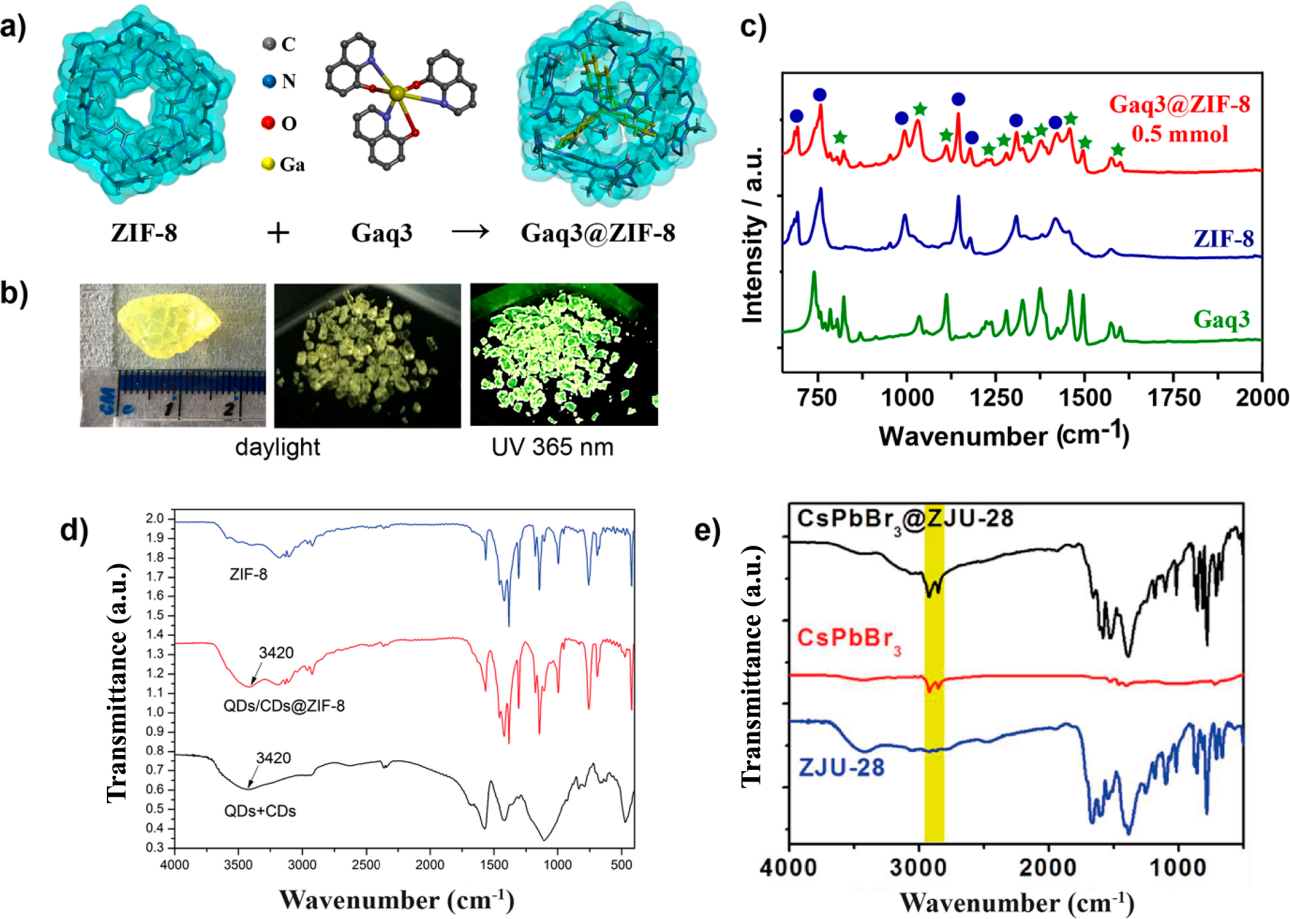
(a) Representation of the chemical structures of ZIF-8 host, Gaq_3_ guest, and the Gaq_3_@ZIF-8 composite material. (b) Large and small fragmented monoliths of Gaq_3_@ZIF8 viewed under daylight and its greenish emission under 365 nm UV lamp. (c) FTIR spectra of Gaq_3_@ZIF-8 showing a combination of the vibrational bands that are characteristic of Gaq_3_ guest (green stars) and ZIF-8 host (blue dots). Adapted from ref (37). Copyright 2020 John Wiley and Sons. (d) ZIF-8, QDs/CDs@ZIF-8, and a mixture of QDs and CDs, adapted with permission from ref (111). Copyright 2017 Royal Society of Chemistry. (e) FTIR spectra of ZJU-28, CsPbBr_3_, and CsPbBr_3_@ZJU-28, adapted with permission from ref (114). Copyright 2020 Elsevier.

Fourier transform infrared (FTIR) spectroscopy is an analytical technique that employs infrared radiation to probe the rotational or vibrational transitions of the molecules, resulting in a spectrum which is a unique fingerprint of the sample (especially in mid-IR regime), making it a versatile and effective technique for the identification of organic molecules or polymers, among others. FTIR spectroscopy is probably, together with the UV–vis/fluorescence spectroscopy, the most exploited technique for the characterization of LG@MOF materials.^67,68,70,110−115^ The simultaneous coexistence of the IR bands characteristics of the fluorophores and the MOF usually serves to identify the presence of the former and to prove the robustness of the latter upon encapsulation, all at once.^67,68,70,110−115^ Numerous and diverse examples have been reported in this sense, such as the presence of different carbon quantum dots (CDs) or metal–organic complexes (like Gaq_3_) confined in the ZIF-8 matrix, see Figure 11a–d.^37,67,68,111^ In those systems, the FTIR spectrum is a combination of the typical IR bands of ZIF-8 and new bands of the luminescent guests like those of Gaq_3_ (Figure 11c) or a broad band at ∼3420 cm^–1^ attributed to the O–H stretching vibration of the CDs (Figure 11d), reflecting the presence of the fluorophores and at the same time the chemical stability of the ZIF-8 after their incorporation.^37,67,68,111^ Similarly, the presence of inorganic perovskite nanocrystals (NCs) in different MOF hosts have been identified by the simultaneous existence of IR bands associated with the specific MOF and to the perovskite NCs, especially the bands at 2924 and 2853 cm^–1^, which are the characteristics signals of the C–H symmetric and asymmetric stretching vibrations of oleylamine and oleic acid capping agents present in perovskite NCs (Figure 11e).^70,114^ The same approach was also followed for the identification of organic dyes like fluorescein in the UiO-66 MOF, where the bands at 1638 and 1643 cm^–1^ were assigned to the C=O vibrations in the benzoquinone structure of the fluorescein molecule.^110^

While FTIR is an outstanding technique for the chemical identification of guest fluorophores in the MOF materials, it is not so suitable to quantify the amount of guest neither to differentiate the position of the guest in the MOF (i.e., encapsulated in the pores, embedded in the crystal, or adsorbed on the surface). An excellent example of this is the protein-embedded cytochrome c (Cyt *c*) in ZIF-8 MOF.^116^ In spite of the detection of the band at 1664 cm^–1^ assigned to the stretching modes of double bonds and carbonyls in Cyt *c*, the authors demonstrated through a calcination process that the Cyt *c* were mostly deposited on the MOF surface rather than being encapsulated in the pores of ZIF-8.^116^ On the other hand, in some very specific cases, the FTIR results can provide information that may suggest the location of the guest. For example, the encapsulation of RhB and fluorescein dyes in the ZIF-8 MOF leads to a suppression of their IR band at 1600 cm^–1^ owing to an increase in their framework rigidity, suggesting that both molecules are well incorporated in the pores of the MOF.^82^ However, some precautions must be taken with these statements, like in the case of the Cu nanocrystals (CuNCs) incorporated into ZIF-90, where the authors observed the lack of the bands corresponding to CuNCs and CuNCs + Al^3+^ at 2080 and 700 cm^–1^ and attributed this phenomenon to the encapsulation of the CuNCs inside the pores, which is very unlikely as the ZIF-90 pore size is ∼3.5 Å,^117^ while the diameter of the CuNCs is of 5.5 nm.^105^ A recent study on the fluorescein@ZIF-8 system has employed a combination of ATR-FTIR measurements and *ab initio* calculations by density functional theory (DFT) to study the phenomenon of guest–host interactions.^83^ The comparison between the simulated IR spectra and experimental observations support the notion that the fluorescein guest can be encapsulated in the cage of the ZIF-8. Notably, a shift in the IR vibrational peaks has been shown to be the result of guest confinement in the cage and the interaction with the host framework.

Although less widely utilized, Raman vibrational spectroscopy could also be a powerful tool for the characterization of LG@MOF systems. For example, vibrational data measured by Raman spectroscopy show similar spectra of the pristine ZIF-8 and the latter containing the fluorescent ZnQ complex, suggesting that ZIF-8 MOF retained its pristine form after the encapsulation of the fluorophore.^32^ A more interesting example is given by the encapsulation of the same ZnQ complex in the Zn-based OX-1 MOF.^35^ In this case, Raman spectroscopy provides further insights into symmetry alterations of the guest by the confinement effect. The analysis shows that the doubly degenerate Raman modes at ∼504 and 514 cm^–1^ (attributed to the skeletal in-plane bending vibrations) of ZnQ turn into a single band at ∼508 cm^–1^ when the fluorophore is encapsulated, reflecting a higher structural symmetry.^35^ Although Raman spectroscopy is a notable complementary technique, it presents similar limitations to those described for FTIR.

### 4.6 Electron Microscopy (SEM, TEM, EDX, FIB)

The scanning electron microscopy (SEM) is a technique that uses a focused beam of electrons to scan the surface of the samples generating an image of the topography usually detecting the secondary electrons emitted by the excited atoms. Although SEM is widely employed for characterizing the morphology of MOF crystals, it does not usually provide key information on the encapsulation of guests. Indeed, most of the examples draw their conclusion pertaining to the morphology of the MOF crystals before and after the encapsulation of guests. For instance, the SEM images of a stilbene-MOF or the bio-MOF-1 after the encapsulation of DCM or RhB dyes show no changes in the morphology, reflecting the stability of both materials after the guest encapsulation step.^94,102^ On the other hand, there exist examples where the encapsulation of guest induces changes in the morphology, but not in the MOF periodic structure. The encapsulation of carbon dots and curcumin into ZIF-8 generates spherical ZIF-8 structures with a rough surface, very different from the regular smooth dodecahedron crystals of pristine ZIF-8.^118^ Although one might be tempted to correlate this morphological change to an effective guest encapsulation, other parameters like changes in the synthetic methodology or the chemical properties of the guests should be considered and therefore additional proofs are required to corroborate the guest encapsulation.

**Figure 12:**
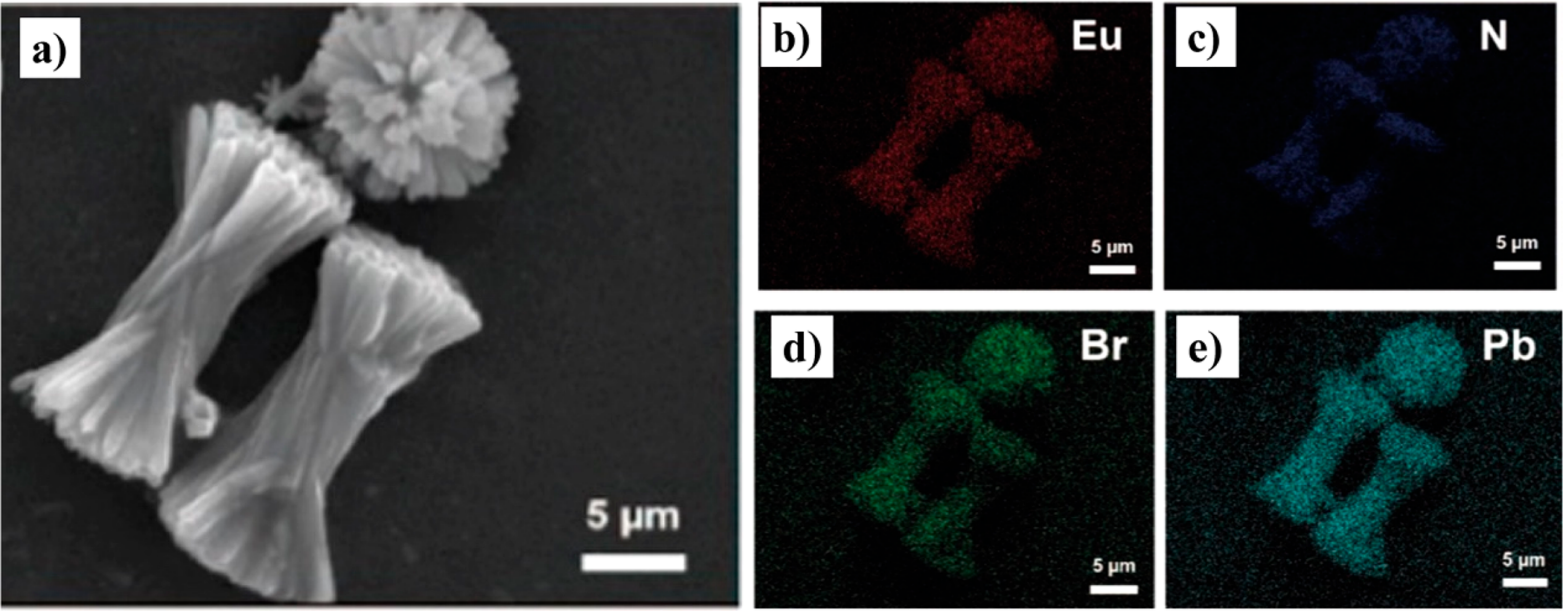
(a) SEM image of EuBTC microcrystals, and the elemental mapping diagrams of (b) Eu, (c) N, (d) Br, and (e) Pb in the CH_3_NH_3_PbBr_3_@EuBTC composite. Adapted with permission from ref (120). Copyright 2018 American Chemical Society.

Notably, the SEM instrument can incorporate multiple detectors to collect the secondary electrons (SE), backscattered electrons (BSE), or the characteristics X-ray. The latter derives into a technique known as energy dispersive X-ray spectroscopy (EDS, EDX or EDXS) which is a type of analytical technique used for the elemental analysis of samples. SEM-EDX has been used for mapping the distribution of guest interacting with MOFs, especially those guest composed of metal-based nanocrystals.^26,64,113,114,119−122^ There are many examples of perovskite nanocrystals interacting with different MOFs, where a mapping distribution of elements like Pb, Br, or Cs (typical components of hybrid and all inorganic perovskites) indicates a homogeneous distribution of the perovskites over the MOF crystals (Figure 12).^26,119−122^ Similarly, the homogeneous distribution of Cu nanocrystals in ZIF-8 and MOF-5 has been analyzed based on the SEM-EDX images of those composite materials.^64,113^ Even though SEM-EDX can shed some light on the guest distribution, there are some limitations such as the difficulty to detect luminescent organic dyes, which are typically composed of light atoms like C, O, and N elements, very similar to the elements that form the organic linkers. Another drawback is the fact that the depth of penetration is not very high, and therefore it is very plausible that the elements detected from the guest are very close or adhered on the surface of MOF crystals, hindering a precise determination of the location of the guests in the MOF host. Focused ion-beam (FIB) milling via Ga^+^ ions has also been employed in conjunction with SEM to remove thin consecutive slices of MOF composite materials, which enabled 3-D reconstructions to study interfaces, volumetric defects, and phase distributions at the micro- and nanoscale.^123−127^ Naturally, this FIB-SEM approach can be extended to enable microstructural examination of LG@MOF systems (e.g., to yield 3-D reconstruction of core–shell materials), provided caution is taken to minimize gallium ion beam damage^128^ to fluorophores and organic moieties.

**Figure 13:**
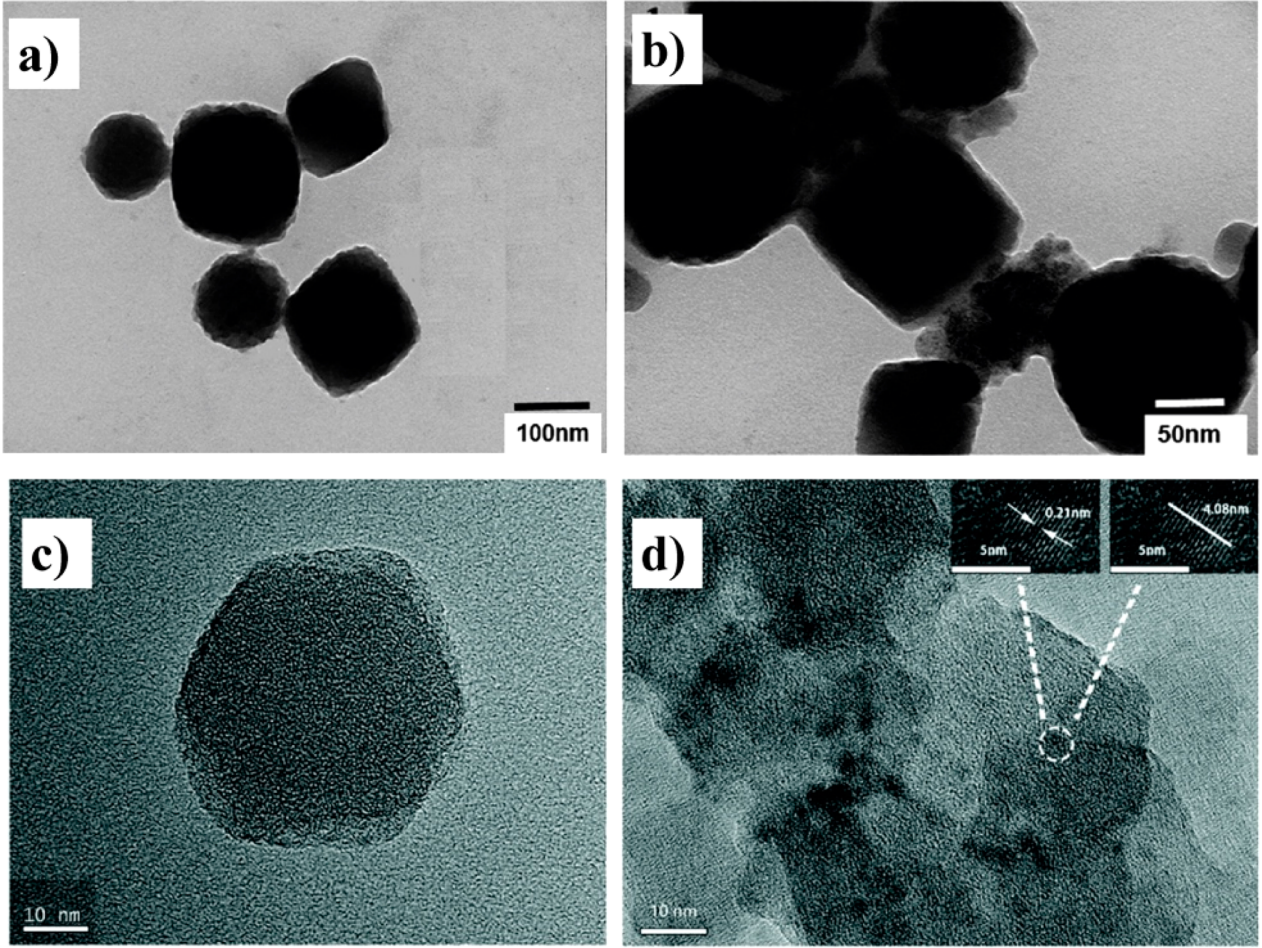
TEM micrographs of (a, b) CDs/Zn-MOFs. Adapted with permission from ref (129). Copyright 2020 Elsevier. (c) ZIF-8. (d) CDs@ZIF-8. Adapted with permission from ref (67). Copyright 2018 Royal Society of Chemistry.

Akin to an SEM, a transmission electron microscope (TEM) also uses a beam of electrons, but in this case, the beam is transmitted through the sample and then magnified and focused by the objective lens onto an imaging device (fluorescent screen) or a charge coupled device (CCD) camera. The maximum resolution of a TEM is considerably greater than an SEM, revealing details at an atomistic scale as low as 1–2 Å. In the field of LG@MOF, TEM has sometimes been used to unveil changes in the morphology of the MOF after its interaction with the guest. For example, the interaction of a perylene derivative with the ZIF-8 MOF produces a change in the morphology (from tetrakaidecahedra to irregular spheres) and the size of the crystals, ascribed to a possible competition of the perylene and the 2-methylimidazolate (mIm) linker for binding the Zn^2+^ ions.^64,113^ However, the most extended use of TEM in this field is to visualize the distribution of quantum dots and nanocrystals in different MOF systems. There are numerous examples where TEM images unveil the presence and in some cases, the position of carbon quantum dots (CDs) in different MOF matrices.^67,112,129,130^ For example, the presence of CDs of about 10 nm in the surface of two different Zn-based and Ln-based MOFs has been demonstrated (Figure 13a-b).^112,129^ In other cases, determination of the location of the CDs is not so trivial, like the formation of CDs after calcination of ZIF-8, where the CDs of a size of around 4 nm give the impression to be entrapped in the ZIF-8 framework (Figure 13c-d).^67^ In other composites, the detection of the CDs is not possible, like in the case of an Eu-MOF system where the authors described that the surface of the rod crystals is smooth, so they have suggested but not confirmed that the CDs may not be attached onto the surface.^130^ Likewise, the TEM technique has been employed to probe the distribution of different quantum dots (e.g., ZnS, CdS, CdTe) within the different MOF frameworks.^34,115,131^

**Figure 14:**
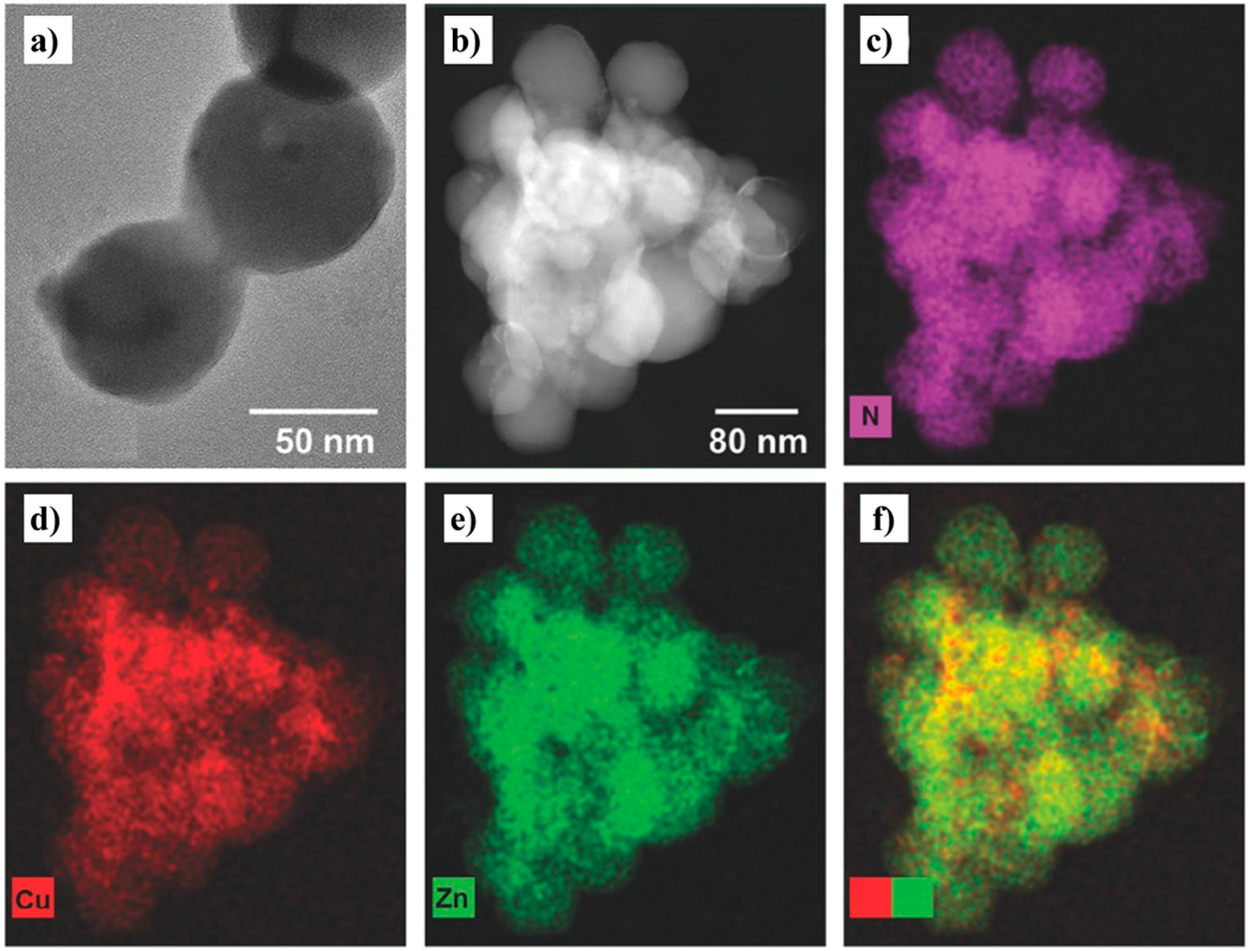
(a) HRTEM and (b) HAADF TEM images of Cu NC/ZIF-8 composites. (c–e) EDS elemental mapping of N, Cu, and Zn (indicated in the figure). (f) Superimposed EDS images of Cu (red) and Zn (green) elements. Adapted with permission from ref (132). Copyright 2017 John Wiley and Sons.

**Figure 15:**
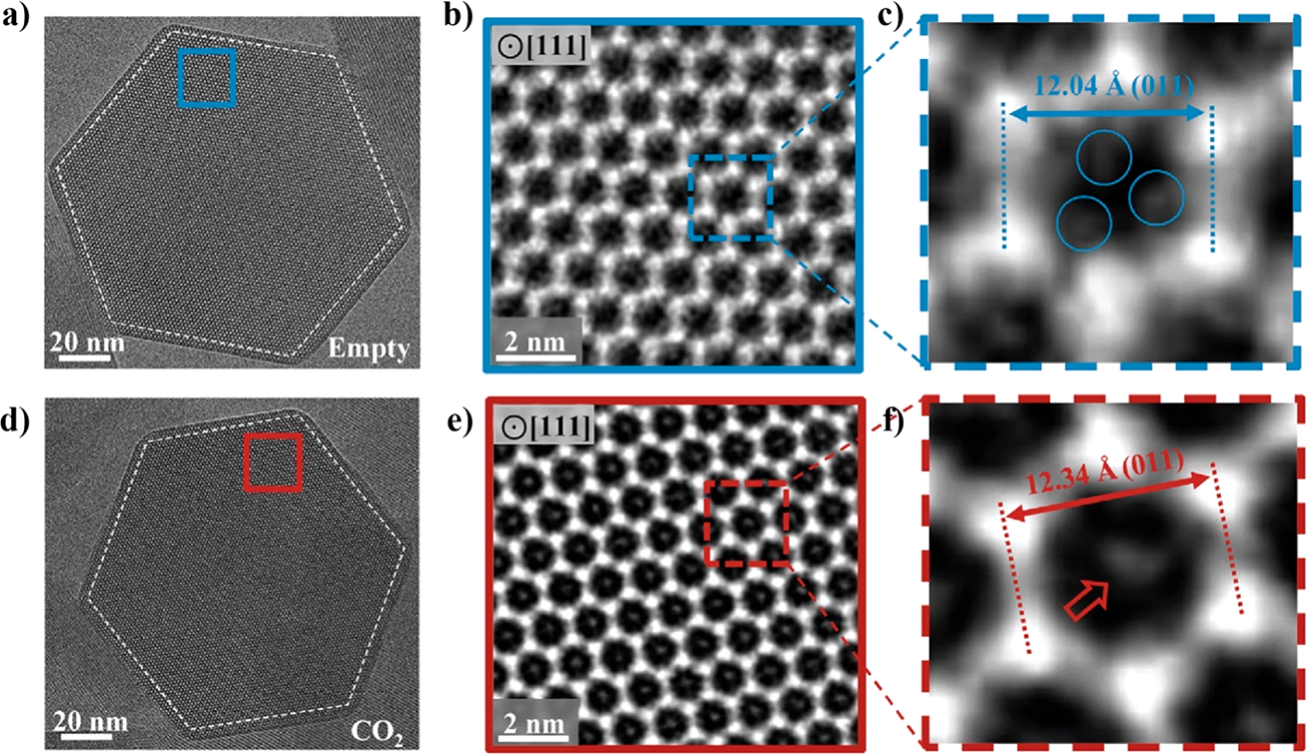
(a, d) Cryo-EM images of the evacuated and CO_2_-filled ZIF-8 crystals, respectively, viewed along the ⟨111⟩ crystallographic axis. (b, e) Contrast transfer function (CTF)-corrected denoised images corresponding to the blue and red boxes on the left panels (a, b), where the bright regions indicate the mass density. (c, f) Magnified images of a unit cell of ZIF-8, where the blue circled regions may correspond to the 2-methylimidazolate (mIm) linkers, while the red arrow possibly indicates position of an adsorbed CO_2_ in the sodalite cage. Adapted with permission from ref (134). Copyright 2019 Elsevier.

Comparable to SEM, TEM images can be combined with EDX analysis for mapping the elemental distribution of a nanomaterial. Even though this is not so frequently employed for LG@MOFs, still some examples can be found. For example, while (high-resolution) HRTEM images of a composite comprising Cu nanoclusters and a ZIF-8 matrix does not provide any evidence of the nanoclusters distribution, an EDX elemental map clearly reveal a homogeneous distribution of Cu atoms all over the ZIF-8 crystals, suggesting a homogeneous dispersion of the nanoclusters (Figure 14).^132^ However, and in addition to the problems related to the location of the guests previously mentioned, and the fact that generally it will not be possible to detect small organic dyes in a MOF structure, TEM also presents major drawbacks like an extensive sample preparation, as it must be thin enough to transmit electrons, making this technique time-consuming. Moreover, there is always a risk of sample irradiation damage by the intense electron beam, reducing the applicability of this technique. Above notwithstanding, the recent advances in HRTEM at cryogenic temperatures (cryo-TEM) and low-dose TEM have opened the door to probe the local structures of MOFs at atomic resolution,^133^ as exemplified by direct imaging of host–guest structures within ZIF-8 (herein guest is adsorbed CO_2_, see Figure 15),^134^ and local structural analysis of the luminescent CsPbX3@MIL-101 composites.^135^ These atomic-resolution imaging techniques have the potential to be applied to pinpoint exact positions of fluorophore molecules confined in the LG@MOF systems.

### 4.7 Thermogravimetric Analysis (TGA)

Thermogravimetric analysis (TGA) is a routine technique for materials characterization in which the mass of the sample is measured over time while the temperature changes. This usually provides information about the phase transition, the adsorption/desorption of molecules (e.g., gas or solvent molecules), or the thermal stability of a material. Indeed, most of the examples reported hitherto in the field of LG@MOFs exploited this technique to ascertain the thermal stability of those composite materials and the presence of solvent molecules.^50,51,77,78,97,115,136,137^ However, there are many other interesting works that leverage the TGA technique to pinpoint the mass percentage of the guest or to appraise whether it is entrapped into the MOF structure.

The loading fraction of RhB dye (ranging from 0.89% to 4.0%) into DUT-52,^138^ the loaded mole ratio of Eosin Y dye (0.16% to 3.66%) in DUT-52,^139^ the content of 4-aminonaphthalimide (0.052%) in a Ln-based MOF^140^ or the weight percentage of anthracene (32%) in ZIF-8^31^ are archetypal examples of the calculation of the amount of dye in MOFs by employing TGA. Other works have used the results obtained from TGA to claim that the dyes are effectively encapsulated in the framework.^94,141,142^ To this end, an enhancement of the thermal stability of the guest is usually employed to justify guest encapsulation.^94^ However, it is worth remarking that similar effect could be observed when the guest is strongly adsorbed on the surface of the material, so the overall thermal enhancement could be a combination of molecules adsorbed and entrapped in the MOF. Because of that, we consider that it is very risky to assert a guest encapsulation taking into account only the results obtained from TGA, while the combination of the latter with other complementary techniques (such as NMR and FTIR) could more accurately unveil the location of the guest in the framework as reported for the anthracene@ZIF-8 and fluorescein@ZIF-8 systems.^31,83^

### 4.8 Other Promising but Less Explored Techniques

#### 4.8.1 Confocal Laser Scanning Microscopy (CLSM)

**Figure 16:**
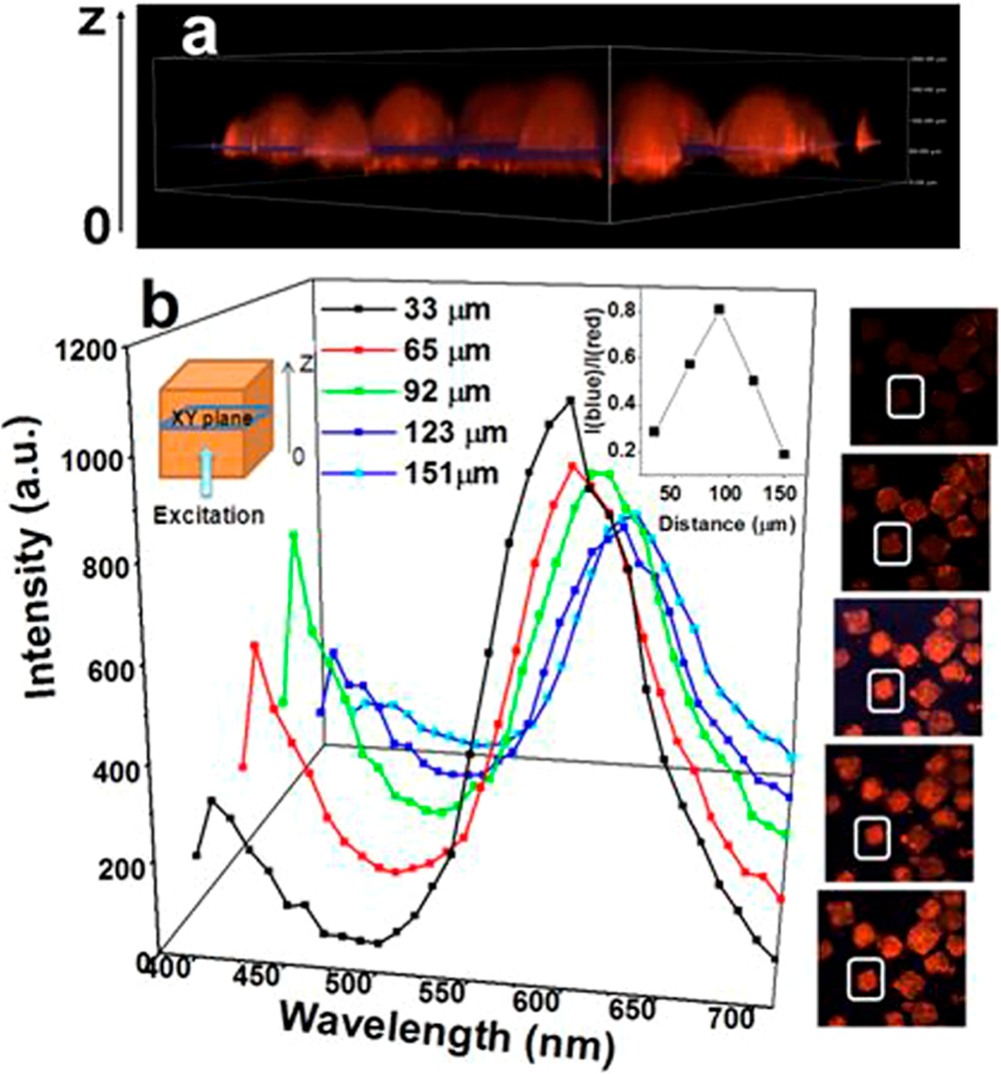
(a) 3-D fluorescence image of DCM@stilbene-MOF. (b) Emission spectra and intensity ratios of blue to red emissions (inset) of a DCM@stilbene-MOF single crystal. The inset photographs are the fluorescence images in focal planes at different distances (33, 65, 92, 123, and 151 μm) from the excitation laser (372 nm). Adapted with permission from ref (102). Copyright 2014 Nature Publishing Group.

A confocal laser scanning microscope (CLSM) is an optical instrument for imaging luminescent samples with an increased optical resolution (compared to fluorescence microscopes) owing to a spatial pinhole that blocks light that is out of focus.^143^ The use of this technique is well spread in the biological and medical research areas, however, a few studies employing CLSM in the field of LG@MOFs are largely focused on the analysis of guest distribution over the MOF crystals. Typical examples have shown a homogeneous distribution of different types of fluorescent dye (e.g., RhB, nile red, porous organic nanosheets, 4-(p-dimethylaminostyryl-1-methylpuridinium) over a number of different MOF crystals (Cd(m-bdc)(bIm), a 2-D Al-MOF, ZIF-8, bio-MOF-1, ZJU-28).^42,75,142,144,145^ Some of these examples attributed the homogeneous distribution of the luminescent guest to an effective encapsulation in the MOF pores. However, it is important to remark that, on one side, the resolution of the confocal microscope is limited (among other things) by the excitation wavelength, providing a typical resolution of 180 nm laterally and 500 nm axially, and therefore, it will not be possible to pinpoint a homogeneous distribution in that nm range; and on the other side, it will be a very difficult task to distinguish whether the emission detected is originated only from the guests adsorbed on the surface, encapsulated in the pores, or a mixture of both. In this sense, a good example is one reported by Yan and co-workers where the encapsulation of DCM dye into a stilbene-MOF is proven by the homogeneous dispersion of the guest shown in different luminescent micrographs recorded in the *z*-axis direction (Figure 16a) and by scanning the *xy*-plane in the *z*-axis direction (Figure 16b).^102^ However, even in this outstanding visual example, it is not possible to completely rule out that part of the emission observed may arise from molecules adsorbed on the surface of the MOF.

**Figure 17:**
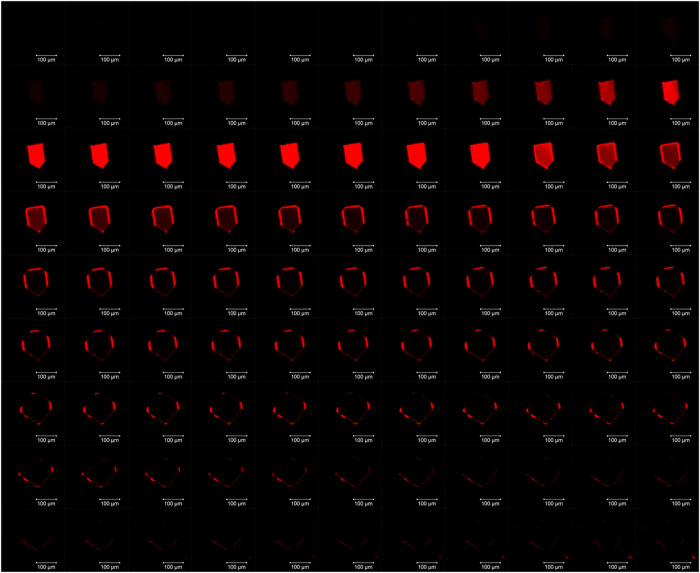
CLSM *z*-stack images of the R6G@In-BTB crystals, using a slicing distance of 1.25 μm. Excitation and emission wavelengths are 488 and 518 nm, respectively. Adapted with permission from ref (147). Copyright 2018 Nature Publishing Group.

On the other hand, in some cases where the size of the MOF crystal is of the tens to hundreds of micrometer scale, it would be possible to determine with better precision the location of the guests within the MOF host.^146,147^ For instance, the confocal imaging in the *z*-axis of rhodamine 6G (R6G) within large micron-sized In-BTB single crystals (where BTB = 1,3,5-benzenetribenzoate) revealed that, the R6G dye molecules were located mostly at the periphery of the In-BTB MOF, and that they cannot fully penetrate into the interior of the crystal, see Figure 17.^147^ From a sliced image of the crystal, the authors estimated a depth penetration of only 5–13 μm for a crystal size of ∼100 μm. In another interesting example, confocal microscopy was proven to be a powerful tool to unveil the presence of BODIPY (boron-dipyrromethene) and resorufin dyes within a cyclodextrin-based MOF (crystal size ∼50 μm), where other techniques such as ^1^H NMR and SCXRD failed to provide the missing evidence.^147^ Moreover, it was possible to pinpoint the location of both dyes on the cyclodextrin-based MOF. While the BODIPY dye was accumulated on the edges of the MOF crystal owing to its bigger size (restrictive confinement effect), the smaller resorufin dye was able to permeate within the crystal fracture planes of the MOF, which presumably are higher polarity regions.^147^

**Figure 18:**
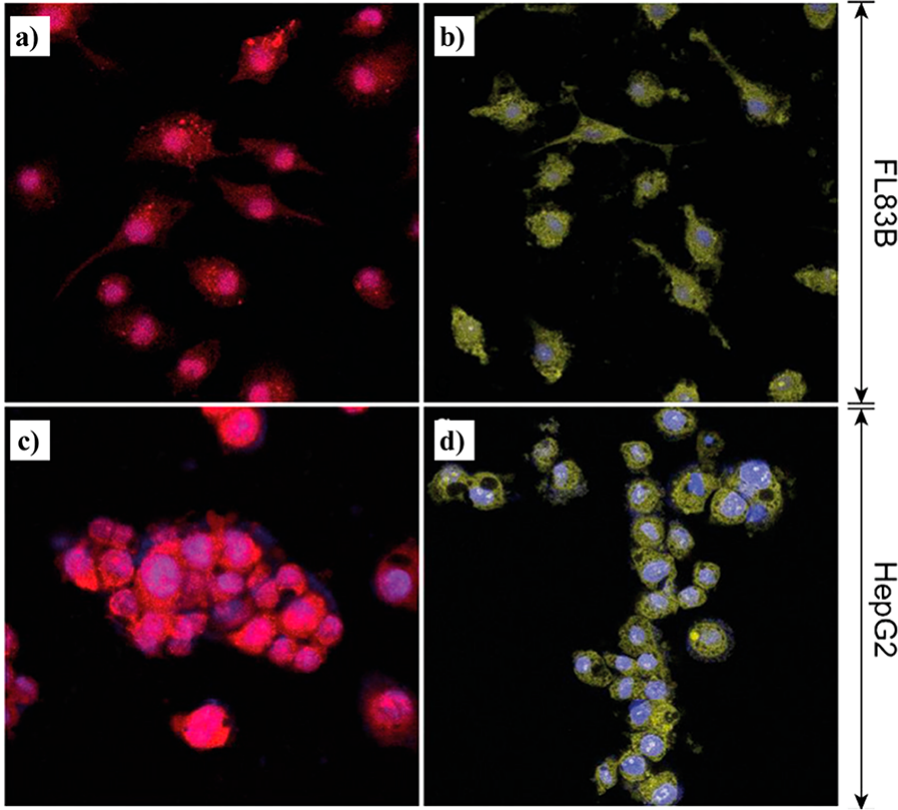
Fluorescence images of (a) FL83B using [Rs@nMOF-801]-GS, (b) FL83B using [R6G@nUiO-67]-GS, (c) HepG2 using [Rs@nMOF-801]-GS and (d) HepG2 using [R6G@nUiO-67]-GS. Adapted with permission from ref (79). Copyright 2017 American Chemical Society.

The potential of CLSM is not only limited to the above experiments, and it has already been shown to be a powerful tool for imaging cells labeled with LG@MOF composites. For example, fluorescence micrographs of FL83B and HepG2 cells after being treated with luminescent Rs@nMOF-801 (Rs = resorufin) and R6G@UiO-67 (R6G = rhodamine 6G) exhibited intense red and yellow emission typically from Rs and R6G dyes trapped in the MOFs, which at the same time are incubated in the cells (Figure 18).^79^

#### 4.8.2 Scattering-type Scanning near-Field Optical Microscopy (s-SNOM) Coupled with Nano-Fourier Transformed Infrared Spectroscopy (NanoFTIR)

**Figure 19:**
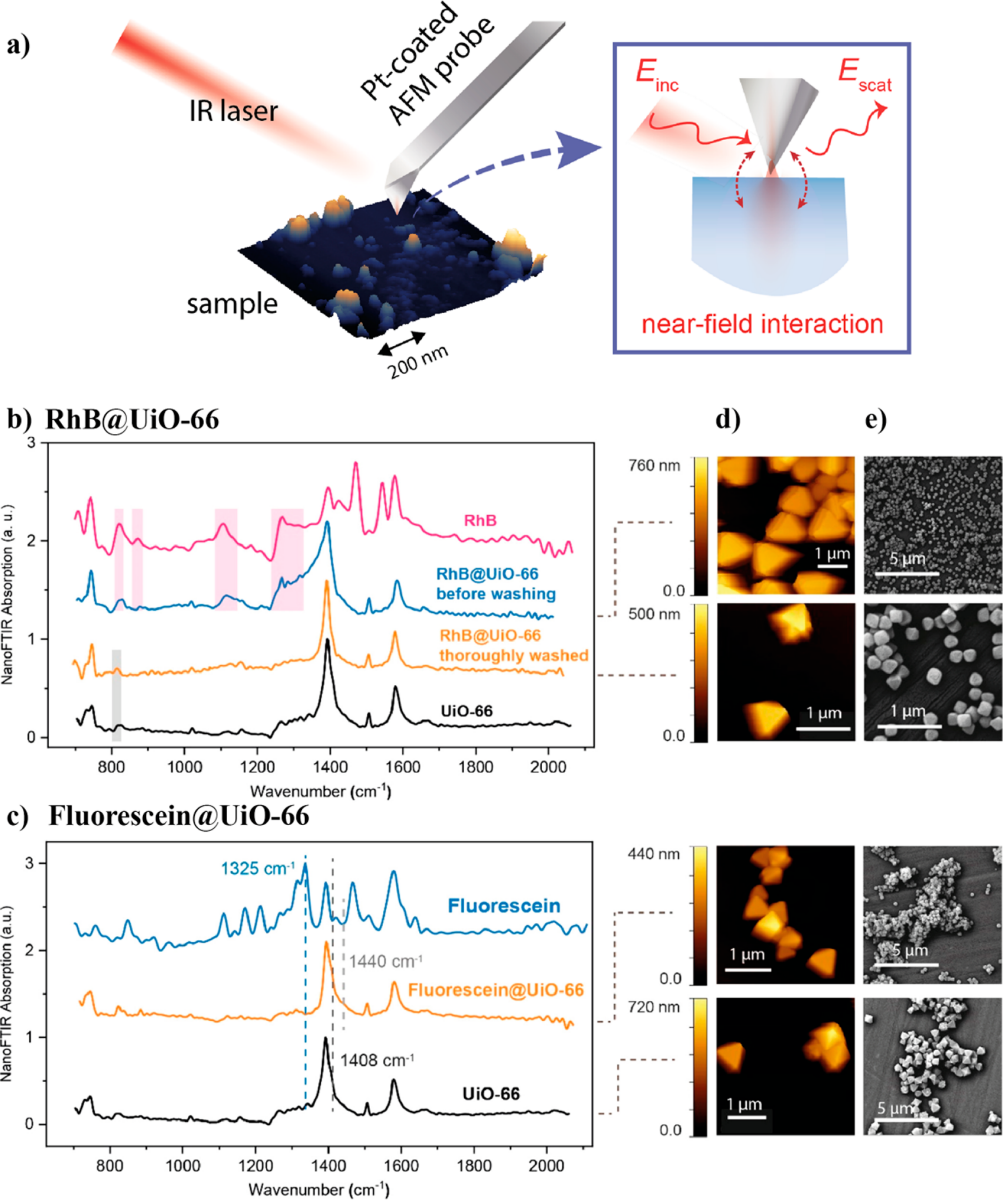
(a) Near-field optical spectroscopy (s-SNOM and nanoFTIR) using a nanofocus with a spot size of ∼20 nm illustrating the near-field interactions between the platinum-coated AFM probe and sample surface polarization upon illumination by a broadband infrared laser. *E*_inc_ and *E*_scat_ denote the incident and scattered light, respectively. (b) Near-field IR absorption spectra of RhB, UiO-66, as-synthesized RhB@UiO-66 composite before and after thorough washing. (c) NanoFTIR spectra of fluorescein, UiO-66 MOF and thoroughly washed fluorescein@UiO-66 composite. (d) AFM and (e) SEM images of UiO-66, RhB@UiO-66, and fluorescein@UiO-66 crystals. Adapted from ref (148). Copyright 2020 American Chemical Society.

A scattering-type scanning near-field optical microscope (s-SNOM) coupled to a nano-Fourier transformed infrared spectrophotometer (nanoFTIR) is a nanoscale local probe technique that simultaneously allows to image (measuring the height topography) and chemically characterize (by IR-active vibrational modes) multiple type of materials at a single crystal level.^148^ This system as illustrated in Figure 19a consists of an atomic force microscope (AFM) where a platinum-coated cantilever tip functions as a topographical and near-field optical probe simultaneously. When the sample is illuminated, the probe induces an evanescent near-field that acts as a nanoscale light confiner, enhancer, and scatterer, paramount to obtain wavelength independent resolution.^149^ This technique, therefore, allows one to collect AFM images while recording the nanoFTIR spectrum of a given material with a resolution down to ∼20 nm. To the best of our knowledge, there is only one report using this technique to characterize LG@MOF materials.^148^ In this work, the authors demonstrate the guest encapsulation by combining s-SNOM/nanoFTIR with CLSM. As the s-SNOM/nano-FTIR is a surface technique, the authors scanned the surface of different LG@MOFs (RhB@ZIF-8, RhB@UiO-66 and fluorescein@UiO-66) crystals before and after a thorough washing step. From those experiments, it can be deduced that before the washing step, it is possible to detect local vibrational modes associated with the guest molecules adsorbed on the MOF surface (Figure 19c, blue spectra). In contrast, after a thorough washing of the sample, there is a lack of vibrational modes associated with the guests, meaning no more guest molecules are adhered on the MOF surface (Figure 19c, orange spectra). Then the authors demonstrated by means of CLSM a homogeneous distribution of fluorescein and RhB guests (with their characteristic green and red emission) over the MOF crystals in the washed samples. Therefore, it can be concluded that the homogeneous distribution of the guests together with the lack of signal derived from the nanoFTIR experiments unambiguously proved that the guests were effectively encapsulated into the MOF crystals. This study shows how complementary techniques and near-field analytics may be employed to verify the location of the guests in a single MOF crystal.

#### 4.8.3 Synchrotron and Neutron-Based Techniques

There are numerous synchrotron- and neutron-based characterization techniques that could be applied to probe a vast range of nanomaterials, soft matter, inorganic and organic compounds, including MOFs and composites. Broadly speaking, these techniques can be divided into categories such as diffraction and crystallography, spectroscopy, imaging, and microscopy. In essence, synchrotron and neutron facilities provide a high-flux source coupled with bespoke sample environments and advanced instrumentations to allow unconventional experiments to be performed under *in situ* and *operando* conditions. Our goal henceforth is not to be exhaustive, but to stimulate readers through several exemplars where the characterization of LG@MOF composite systems has benefited (or might benefit) from the application of state-of-the-art techniques at large science facilities.

X-ray absorption spectroscopy (XAS)^150^ can offer additional structural information in addition to diffraction techniques since the latter is generally limited to the solution of an average structure. Particularly, XAS can reveal the local coordination within the inorganic cluster, and in doing so, shed light on the local structure and its deviation from the symmetry of the overall structure.^151^ In the context of LG@MOF systems, it is important to understand how the encapsulation of the fluorophores may distort the coordination environment surrounding the metal centers of the MOF host, and to locate the position of the guest species. Mustafa et al.^152^ reported a luminescent Eu@COK-16 system, where they employed a combination of XAS and XRD refinement techniques to determine the location of the exchanged Eu^3+^ ions within the COK-16 host. The obtained spatial arrangement helps to elucidate the guest–host interaction facilitating intermolecular energy transfer processes, which underpin the luminescence behavior of this host-sensitized composite. In a related study concerning encapsulation of metal NCs within the Zn-based OX-1 MOF, Titov et al.^153^ demonstrated the implementation of synchrotron XAS techniques (XANES and EXAFS) to study the local structures around the Zn and Pd atoms in the Pd@OX-1 system. Interestingly, modeling of the XAS data revealed that the NCs in the composite are predominantly small clusters of Pd atoms, which are strongly interacting with the bdc linkers of OX-1. Zn K-edge measurements confirmed that the encapsulation of Pd guest, due to its small NCs, does not alter the framework structure around the Zn centers of the OX-1 host.

Synchrotron-radiation infrared (SR-IR) spectroscopy presents many advantageous compared with the conventional benchtop FTIR instrument because it is broadband and has a very high flux, thereby ideal for acquiring high-resolution broadband vibrational spectra of MOF materials, especially to probe low-frequency phonons in the far-IR regime (terahertz frequencies ≲10 THz).^154^ These THz modes are important as they are underscoring the elasticity, structural stability, guest–host dynamics, phase transformation and functions of all framework materials.^155−159^ When combined with inelastic neutron scattering (INS), all vibrational modes from 0–4000 cm^–1^ can be measured since there is no optical selection rules when employing neutron as a probe.^160^ However, detailed assignment of the vibrational modes will often require further insights derived from DFT simulations as exemplified by these studies.^154,159,161^ Particularly, the theoretical modeling of the host–guest interactions of a bulky fluorophore with a MOF host is not trivial, this is a challenging task for DFT as demonstrated in this recent work on the fluorescein@ZIF-8 system.^83^ Finally, it is worth emphasizing that the high spatial and temporal resolution afforded by the synchrotron and neutron sources permit the *in situ*/*operando* experiments to investigate host–guest interactions and to gain insights into basic mechanisms subject to different physical and chemical stimuli, such as UV irradiation,^162^ temperature,^163^ pressure,^164^ and various kinds of guest molecule.^165−170^

In terms of the 3-D imaging of MOF crystals using synchrotron irradiation, recently, techniques such as the full-field transmission X-ray microscopy (TXM) nanotomography has been demonstrated by Mayorga-Gonzalez et al.^171^ In this study, a single crystal of MIL-47(V) with its smallest cross-section of ∼10 μm has been reconstructed to yield a 3-D map revealing the internal microscopic porosity for subsequent quantitative analysis. Surprisingly, the results derived from TXM show that the actual porosity level was only 2–3 vol %, but this value has been greatly overestimated by Hg-intrusion by a factor of about ten times. Related to the above is scanning tunnelling X-ray microscopy (STXM), a spectro-microscopy technique resembling SEM/TEM imaging, but enables one to perform nanoscale chemical imaging using X-ray photons.^172^ Another 3-D imaging technique demonstrated was full-field tomographic XAS,^173^ where a defect-engineered HKUST-1 single crystal (∼50 μm) as studied to visualize its spatio-chemical heterogeneities and identify secondary phases at a resolution of ∼2 μm. It will be exciting to explore the use of synchrotron 3-D imaging tools to interrogate LG@MOF systems; for example, to visualize micro/nanostructural defects and to quantify chemical heterogeneities of NCs@MOF and QDs@MOF composite crystals, as well as to interrogate the sample performance under *in situ*/*operando* conditions.

## 5 Guest–Host Phenomena: Excited State Events

Excited state proton transfer (ESPT), charge transfer (CT) and Förster resonance energy transfer (FRET) phenomena are key events in many natural (e.g., photosynthesis, biological processes) and mimicked artificial systems (e.g., solar cells, OLEDs, photocatalysis), being the keystone of many of the modern advanced photonic and optoelectronic technologies.^174−177^ Thus, a proper control and understanding of those excited state mechanisms is paramount for a better development of advanced functional materials, and consequently, for the fabrication of the next generation of optoelectronic and photonic devices. Over the past two decades, the steady-state and ultrafast photodynamics of the ESPT, CT, and FRET processes have been well documented in different guest–host composites, such as guest encapsulated into different silica-based mesoporous (SBMs) systems.^92,178,179^ However, to ensure a sustainable worldwide growth, there is an urgent need of developing more efficient materials that will bring new practical and cost-effective solutions to address the global challenges, such as renewable energy generation and its efficient use, among others. To this end, the tunable physicochemical properties of MOFs have turned them into one of the most promising materials to explore, improve and exploit these excited-state phenomena. In the following sections, we will describe the basic theory behind those photophysical events, including some of the most representative examples in the field of MOFs.

### 5.1 Förster Resonance Energy Transfer (FRET)

FRET is a nonradiative physical event in which a photoexcited fluorophore (donor) will transfer the energy to an acceptor pair. Several criteria must be satisfied in order to harvest the energy efficiently: (i) First, the distance between the donor and acceptor entities must be sufficiently close (typically 10–100 Å), as the efficiency of the process (η) decreases with the sixth power of the distance:
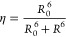
where *R*_0_ stands for the Förster radius (distance at which the energy transfer is 50% efficient), while *R* is the donor–acceptor distance. (ii) Second, there must exist an overlap between the emission spectrum of the donor species and the absorption spectrum of the acceptor. (iii) Third, the orientation of the dipole moment of the donor and acceptor entities must be sufficiently good (typically should be parallel) to allow a dipole–dipole coupling as
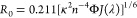

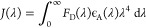
where κ is a factor describing the transition dipoles in space (generally assumed as 2/3), *n* is the refractive index of the medium, Φ is the quantum yield of the donor in the absence of the acceptor, and *J*(λ) is the overlap integral, which at the same time depends on the fourth power of the wavelength (λ^4^), the emission of the donor normalized to an area of 1 (*F*_D_(λ)), and the extinction coefficient of the acceptor at a given wavelength (ε_A_(λ)). Albeit not a critical requirement, it is desirable that both, the donor and acceptor fluorophores exhibit a high fluorescence quantum yield.

On the basis of the foregoing statements, it is clear why MOFs are an excellent platform to trigger the FRET mechanism. On the one hand, their accessible pore cavities can allow the encapsulation of a vast range of fluorophores, which will shorten the donor–acceptor distances. On the other hand, the almost infinite combination of luminescent MOFs (LMOFs) and types of fluorophore guest (Section 3) provides a unique opportunity for enhancing the spectral overlap through a judicious selection of the donor and acceptor entities. Moreover, the crystalline structure of MOFs allows one to predict the position of the donor and acceptor fluorophores, offering a promising pathway for controlling directional FRET, which can occur from the MOF to the guest or *vice versa*.^96,180−182^

**Figure 20:**
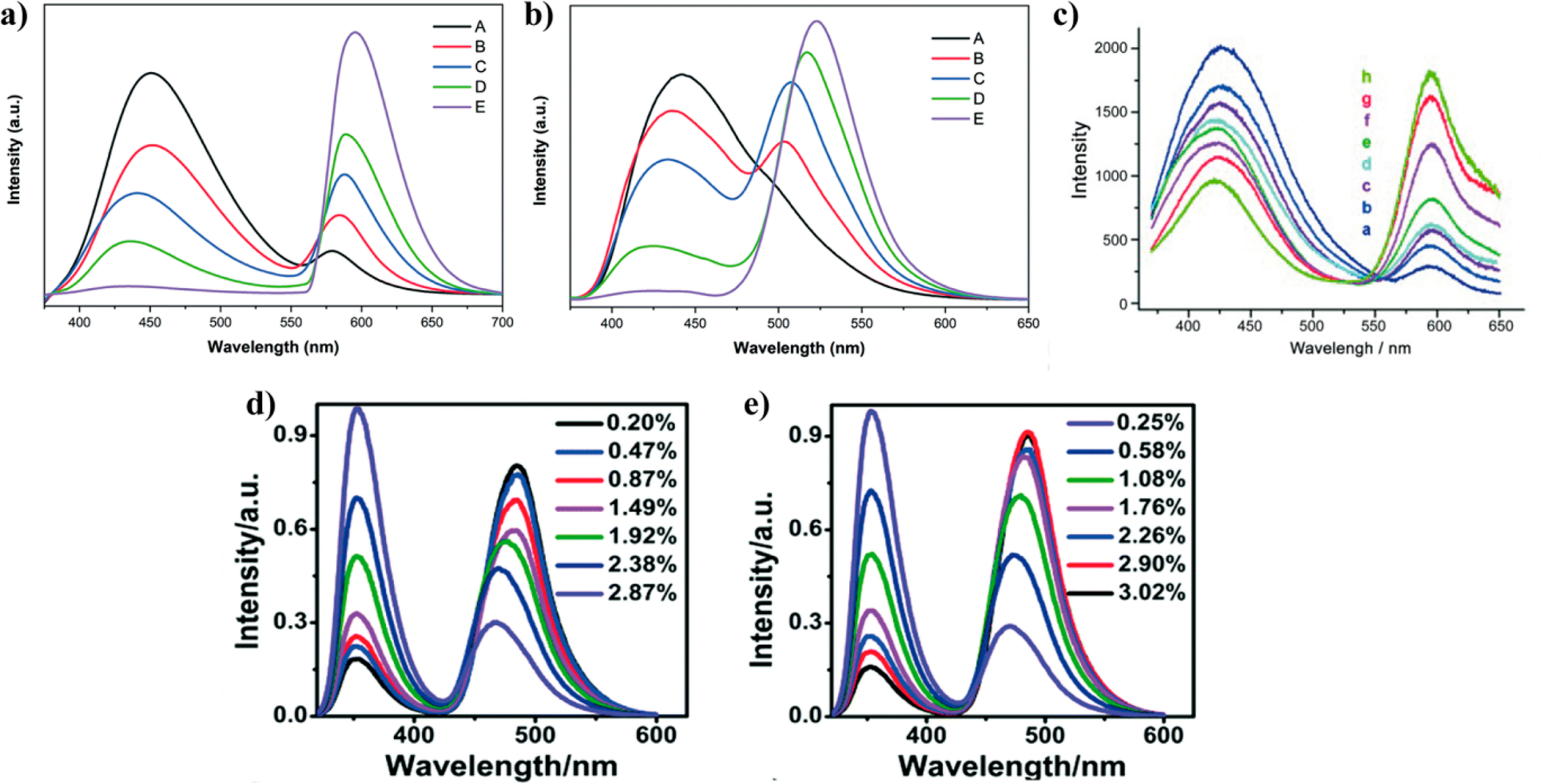
(a, b) Emission spectra of (A) DCM@MOF and (B) C6@MOF with different concentration of dyes: A, 1 × 10^–6^ mmol L^–1^; B, 5 × 10^–6^ mmol L^–1^; C, 1 × 10^–5^ mmol L^–1^; D, 5 × 10^–5^ mmol L^–1^; E, 1 × 10^–4^ mmol L^–1^. MOF = zinc framework with carbazole-based linkers. Adapted with permission from ref (88). Copyright 2018 Royal Society of Chemistry. (c) Emission spectra of RhB@CZJ-3 with different concentration of RhB: a = 0.02 wt %, b = 0.04 wt %, c = 0.06 wt %, d = 0.08 wt %, e = 0.1 wt %, f = 0.12 wt %, g = 0.16 wt %, and h = 0.19 wt %. Adapted with permission from ref (86). Copyright 2014 John Wiley and Sons. (d, e) Emission spectra of coumarin/M-BTB MOFs with different levels of coumarin loading. Adapted with permission from ref (87). Copyright 2017 Royal Society of Chemistry.

Some of the most powerful tools to unravel FRET processes are based on steady-state and time-resolved emission techniques such as fluorometer, time-correlated single photon counting (TCSPC), or femtosecond (fs) up-conversion techniques. Typically, one of the first observation in a FRET process is the quenching of the emission intensity of the donor system. Although this is not an indisputable scientific evidence (as other excited state processes may produce the same phenomenon), it could be an indication of a FRET mechanism. Indeed, a very common experiment is to load the MOF with an increasing amount of guest dyes to incrementally show the quenching of the emission intensity of donor, and the enhancement in the emission intensity of the acceptor. Some of these findings are summarized in Figure 20, where different amounts of coumarin, RhB, or DCM fluorophores were encapsulated into different MOFs, showing incremental quenching of the MOF emission when subject to a greater concentration of guest.^86−88^

A more reliable proof can be obtained by measuring the excitation spectrum, selecting the emission (observation) wavelength in a region where the emission of the acceptor can be detected, but where there is no emission signal from the donor. If there is a FRET process, then the excitation spectrum must show the feature of the spectrum (usually comparable to the absorption one) of the donor counterpart. For example, the excitation spectra of a series of lanthanides in the bio-MOF-1 (Tb@bio-MOF-1, Eu@bio-MOF-1 and Tb/Eu@bio-MOF-1) display a band in the range of 230–400 nm, which matches the absorption of the MOF linker (4,4-biphenyldicarboxylic acid), reflecting the sensitization of the lanthanides by the latter.^108^ A more visual example is reported for the energy transfer process occurring from a Zr-NDC MOF to different organic fluorophores (coumarin 153 (C153), nile red (NR) and DCM). In those materials, the excitation spectra–recorded in spectral regions where the MOF is not emitting but there still some emission of the dyes–clearly show the vibrational bands of the 2,6-naphthalenedicarboxylate (NDC) linkers of the MOF, thereby unequivocally proving the occurrence of a FRET process from the MOF to the guests.^85^ Similarly, the FRET process from a Hf-MOL (metal organic layer) to the coumarin 343 (C343) was confirmed by monitoring the excitation spectrum at 475 nm (emission of C343), which exhibits two peaks corresponding to the direct excitation of C343 (445 nm) and the linker of the MOF (290 nm).^87^

Time-resolved experiments can help to decipher the photodynamics of the FRET mechanisms alongside the lifetime of donor and acceptor entities. Here it is generally important to distinguish between the emission regions of the donor and the acceptor. In the emission region of the donor, the lifetime (τ_D_) should decrease as the emission will be quenched. Additionally, if the time resolution of the system is good enough, one can observe an additional shorter time component with a positive amplitude that may correspond to the time of the FRET phenomenon. On the other hand, the lifetime of the acceptor (recorded at longer wavelengths) may depend on the surrounding environment, as changes in the polarity, acidity/basicity, or even the confinement effect will alter this value. In addition to that, and again, if the time resolution of the instrument is adequate, it is possible to observe a new shorter time component, comparable to that observed in the region of the donor, but now with a negative amplitude. This negative amplitude (rise component) reflects the population of the excited state of the acceptor moiety, and therefore, this will also correspond to the duration of the FRET phenomenon.

**Figure 21:**
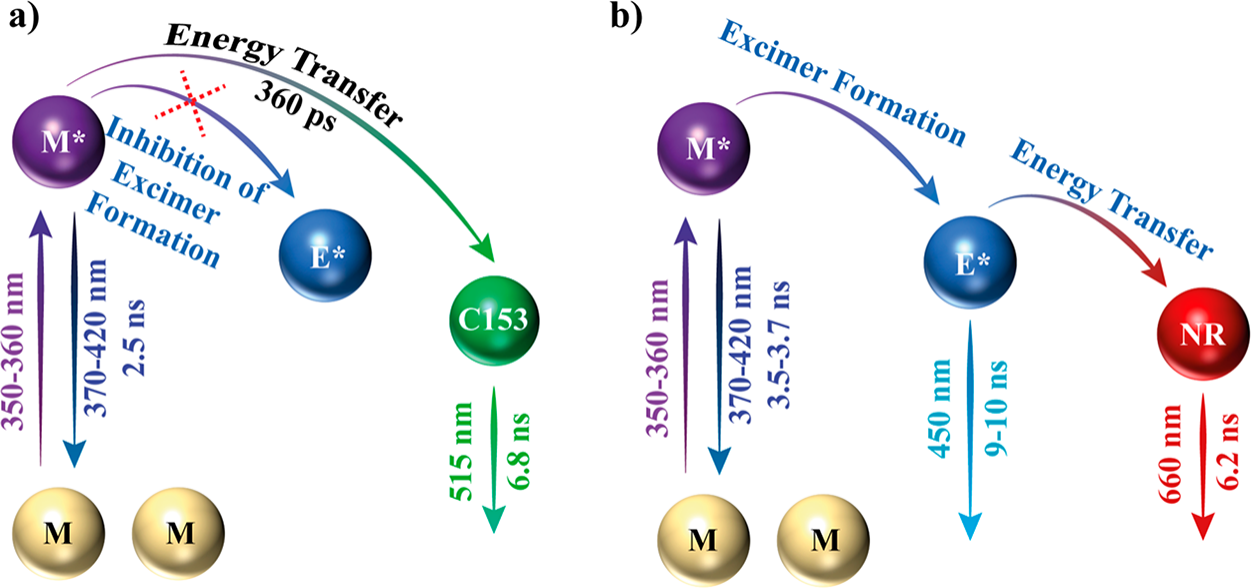
Schematic representation of the underlying photophysical processes after the photoexcitation of (a) C153@Zr-NDC and (b) NR@Zr-NDC MOF composites. In scheme (a), the encapsulation of C153 inhibits the formation of the excimeric species, and the energy transfer occurs from the monomers (denoted as M). However, in the case of NR@Zr-NDC (scheme b), first the excimers are formed and then those transfer their energy to the NR dye. Adapted with permission from ref (85). Copyright 2015 Royal Society of Chemistry.

Although it is primordial to unveil the ultrafast dynamics of FRET processes in order to enhance the efficiency of this mechanism, the number of studies focusing on the photodynamics of LG@MOF undergoing FRET is much lower than for other systems such as Guest@SBMs. Still some interesting examples can be found in the literature.^85,118,183,184^ For example, the encapsulation of rhodamine 6G (Rh6G) into the MOF [Zn_6_L_4_(Me_2_NH_2_^+^)_4_·3H_2_O] yields a material showing a highly efficient energy transfer (98.8%).^183^ In this system, the average lifetime of the MOF (donor) in the absence of the Rh6G (acceptor) is 7.63 ns, while increasing amounts of Rh6G in the pores of the MOF decreased the lifetime to 0.16 ns.^183^ Similarly, the energy transfer from carbon dots (CD) to curcumin (CCM) dye, both entrapped into ZIF-8 MOF, was demonstrated by the quenching of the τ_D_ (CD) from 5.77 ns in the absence of CCM to 3.03 ns in the presence of the latter.^118^ Another analogous example is given by the decrease in the lifetime of a BODIPY (donor) encapsulated in the nPCN-222 MOF (acceptor).^184^ A detailed ultrafast photophysical characterization of a FRET from a Zr-NDC MOF to different guest dyes was reported by Douhal et al.^85^ In this work (mentioned also above), the authors encapsulated 3 different dyes (C153, NR, and DCM) in the pores of Zr-NDC, whose emission may originate from linker monomers or excimers. It was demonstrated that for C153@Zr-NDC, the lifetime of the monomers was quenched, however, for NR@Zr-NDC and DCM@Zr-NDC the lifetime corresponding to the excimeric species was the one which suffers a decline. This result indicates that in the first case, the transfer of energy takes place from the monomers of Zr-NDC to the C153, while in the other systems it occurs from the excimer of the MOF to the guest dyes (Figure 21). Moreover, the authors also detected a shorter component (hundreds of ps) with positive (donor emission region) and negative (acceptor emission region) amplitudes, attributed to the time of the FRET process (Figure 21).^85^ Many of these LG@MOF composites undergoing FRET have a great potential for many photonic applications, such as luminescence sensing or solid-state lighting (see Section 6).

### 5.2 Photoinduced Charge Transfer (PCT)

Photoinduced charge transfer (PCT) is a photophysical process that involves the transfer of charge (or an electron) from a photoexcited electron donor to an electron acceptor system. This process can be either intramolecular, when the electron donor and acceptor belong to the same molecule, or intermolecular in the case when the electron donor and acceptor are different molecules. The PCT is one of the most important photophysical events in nature as it is the driving force of essential biological phenomena, such as the photosynthesis or the human vision.^185^ In addition to that, researches have mimicked these processes to develop some of the most important advanced technologies, such as optoelectronic devices (solar cells, OLEDs), photocatalysts, or chemical sensors.^174−177,186,187^ Hence, the control and manipulation of PCT through the design, fabrication and characterization of novel materials are paramount to enhance the efficiency of such technologies. The synthetic flexibility of MOFs coupled with their porosity, make these materials ideal scaffolds to explore the PCT phenomenon. One of the simplest and most exploited method to induce PCT in MOFs is through the design of redox-active or electron-rich, or electron-deficient organic linkers; this topic has been summarized in a recent review by Deria and co-workers.^188^

**Figure 22:**
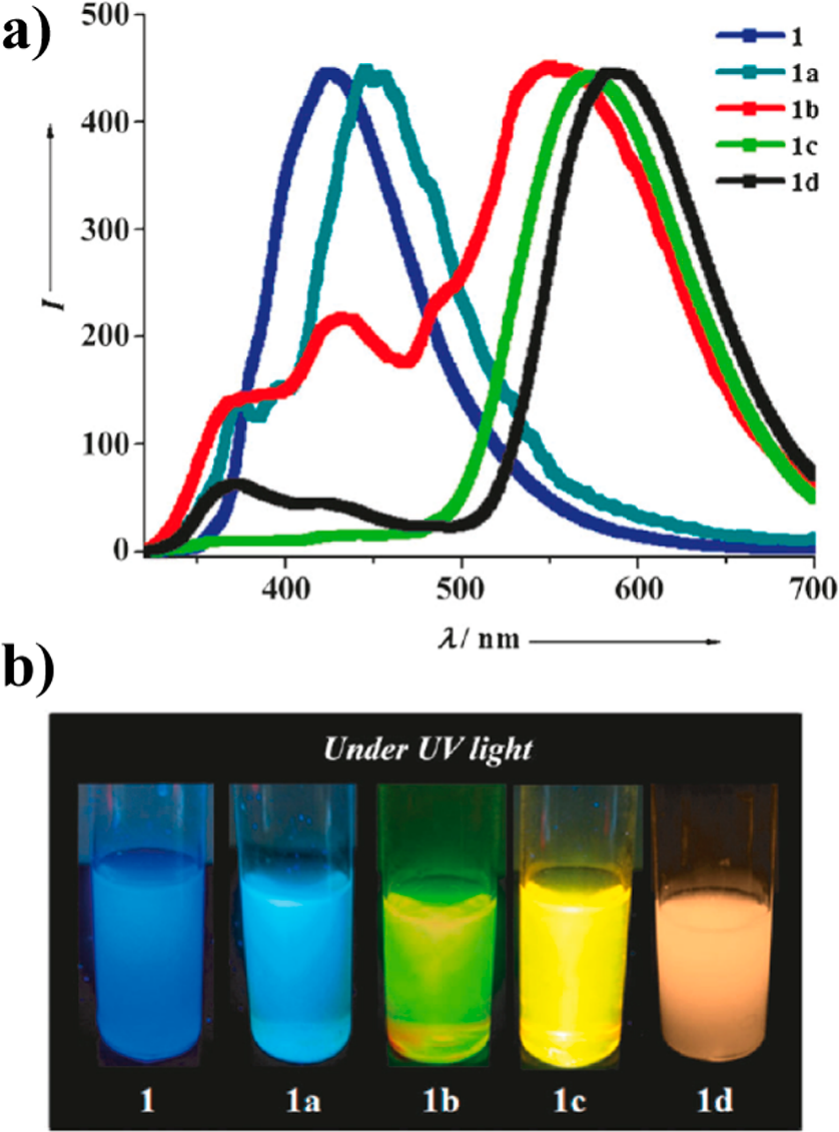
(a) Emission spectra of [Zn(ndc)(o-phen)]·DMF (1) MOF before and after the inclusion of aniline (1a), N-methyl aniline (1b), N,N′-dimethyl aniline (1c), and N,N′-dimethyl-p-toluidine (1d), upon excitation at λ = 330 nm. (b) Photographs of compounds 1 and 1a–1d after being dispersed in methanol and under UV light irradiation. Reproduced with permission from ref (84). Copyright 2014 John Wiley and Sons.

However, and more in line with the current review focusing on LG@MOF systems, another interesting approach is the encapsulation of PCT-active molecules in the pores of the MOF. In one of the first reports in this subject, Allendorf et al., proposed a possible PCT process from the linker (1,3,5-benzenetribenzoate) of the MOF-177 to the encapsulated [6,6]-phenyl-C61-butyric acid methyl ester (PCBM) molecule.^189^ Although it was not possible to prove it experimentally, the authors have reasoned that this PCT exists based on the emission quenching observed for the MOF and on theoretical calculations, which predicts the possibility for a charge transfer to occur.^189^ Thereafter, a variety of PCT-active compounds have been encapsulated in the pores of multiple MOFs.^84,188,190,191^ Indeed, an interesting strategy to detect electron-rich or electron-deficient analytes (e.g., explosives and pollutants) is based on the employment of active linkers to provoke a PCT reaction from or to the encapsulated guests. These host–guest interactions will alter the photophysical properties of the MOFs by quenching, enhancing, or shifting (change in color) of their emissions. For instance, there exist many examples of luminescent MOFs which are able to detect nitroaromatic explosive-like molecules due to their emission quenching produced by a PCT from the MOF to those electron-deficient molecules.^192−195^ Another intriguing example has been reported by Maji and co-workers, where different electron-donating aromatic amines were encapsulated in the pores of a blue light emitting Zn-based MOF ([Zn(ndc)(o-phen)]·DMF).^84^ The strong CT interactions between the amines and the 1,10-phenanthroline (o-phen) linker produce a large shift in the emission, resulting in multicolor fluorescence, which is dependent on the aromatic amine (Figure 22); the results show the outstanding ability of this MOF to detect specific analytes that can be easily observed by the naked eye.^84^

In addition to the aforementioned exemplars, another strategy is to encapsulate fluorophores undergoing photoinduced intramolecular charge transfer (ICT). Time-resolved transient absorption and emission techniques have proven to be a very powerful tool to unveil these PCT processes.^190,191^ For instance, it has been possible to interrogate in real time the ICT phenomenon taking place in the fluorophore NR dye, when it is interacting with a series of 2-D MOFs.^190,191^ Depending on the 2-D MOF, this ICT process in the NR dye occurred in times as short as 1 ps or 425 fs. Similar to the previous example, the authors took advantage of this PCT process to characterize the interactions between the electron-rich aniline and dimethylaniline aromatic molecules and the NR dye. This investigation was carried out by exposing the LG@MOF to vapors of the aromatic amines and by functionalizing the MOF with aniline, showing in both cases a strong CT interaction from the analytes to the NR dye. In fact, the authors reported an ultrafast electron transfer from aniline to NR molecules happening over a short time span of 17 ps.^190,191^ This is therefore another interesting mechanism for the detection of electron-rich or electron-deficient toxic vapors by leveraging LG@MOF systems.

### 5.3 Excited-State Proton Transfer (ESPT)

The excited-state proton transfer (ESPT) is a process in which a photoexcited species transfers a proton (H^+^) from a donor group (typically −OH, −NH, −SH) to an acceptor (e.g., C=O, −N=). This is a photoinduced process, as the irradiation with photons induces a change in the electronic distribution of the molecule, affecting the acidity/basicity of the proton donor and acceptor groups, which at the end controls the efficiency of the process. Similar to the PCT, the ESPT process can be either intramolecular (ESIPT), when the proton donor and acceptor groups are distributed within the same molecule, or intermolecular, when the donor and acceptor groups belong to different molecules.^196^ The ESPT phenomenon produces, therefore, a tautomerism between the enol and keto, or the conversion to ionic species (anion or zwitterion), which exhibit a different optical and photodynamical behavior. In the case of an enol-keto tautomer, the absorption spectrum remains unchanged, as the proton transfer occurs after photoirradiation. However, the emission spectra will be very different as a function of the emitting species. The emission of the enol tautomer is more energetic, while the emission of the keto species appears at larger wavelengths, the Stokes shift being much higher. The possibility of tuning a dual-color emission from the same molecule, alongside with the fact that the proton transfer reaction is one of the most common phenomena in several chemical and biological processes, has made ESPT one of the most well characterized mechanisms. Since the pioneering works of Weller,^197,198^ Weber,^199^ and Förster^200^ in the 1950s, this subject has been boosted through the design, synthesis and detailed characterization of molecules, materials, and composites that undergo ESPT.^92^

**Figure 23:**
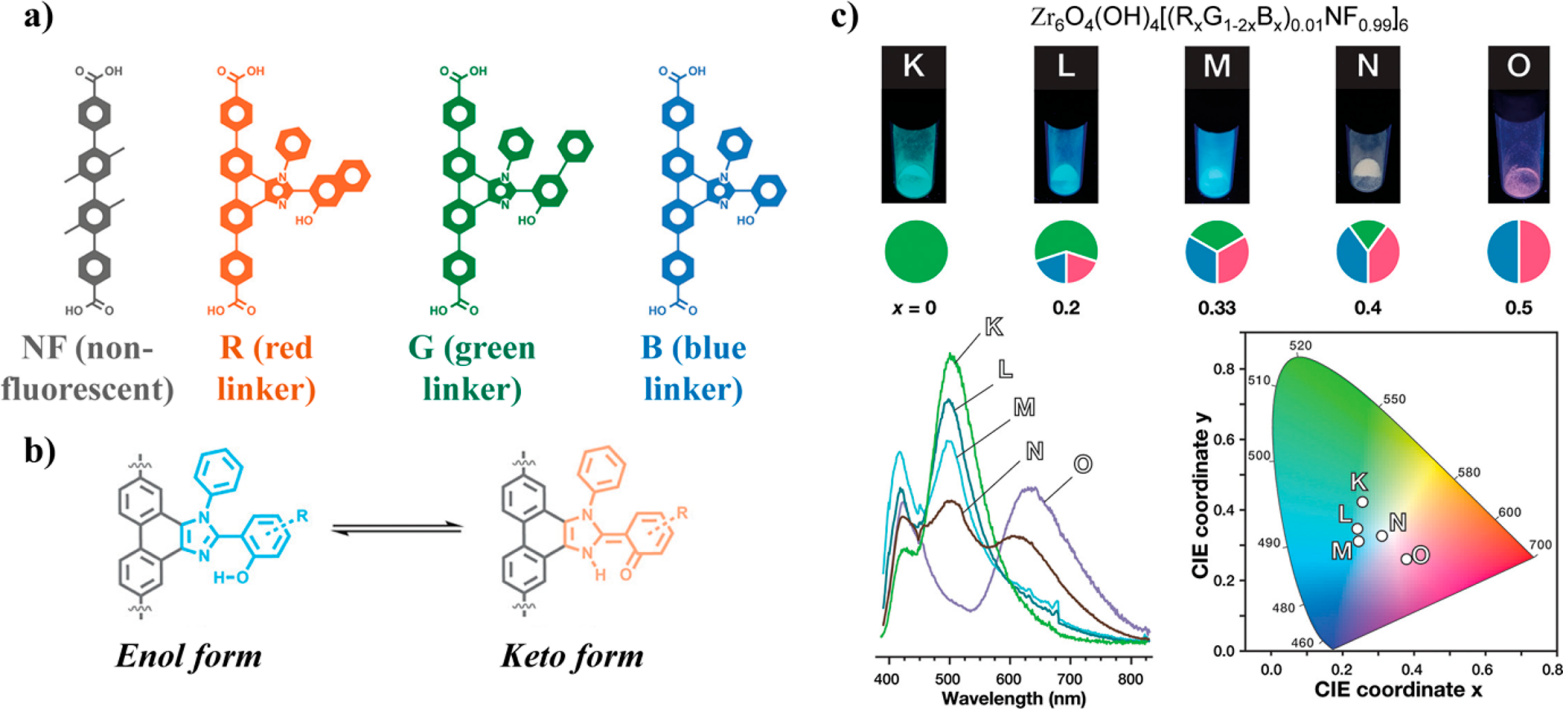
(a) Multicolor emitting organic linkers exhibiting ESIPT. (b) Schematic representation of the enol and keto phototautomerism. (c) Photographs taken under UV light irradiation (λ_ex_ = 365 nm, top), emission spectra (bottom left) and CIE coordinates (bottom right) of MOFs having different ratio of R, G, and B ESIPT linkers. Adapted with permission from ref (208). Copyright 2019 American Chemical Society.

Surprisingly, the ESPT mechanism has not yet been extensively exploited in the MOF systems, and just a handful of examples hitherto have been reported.^201−209^ Most of them, are based on MOFs where the linker can undergo an ESPT, such as 2,5-dihydroxyterephthalic acid (DHT). For instance, a number of different metal-based MOFs (Mg, Cd, Y) have been reported whose photophysical properties are governed by an ESIPT reaction within the DHT linker.^203−205,207,209^ As the efficiency of the ESIPT process, and therefore the enol-keto tautomerism, will be directly influenced by the surrounding environment, these MOF materials exhibit excellent capabilities toward the detection of many chemical compounds such as different organic solvents,^202−205^ water,^207^ cations,^203,207^ anions,^204,207^ and vapors of different amines.^209^ Similarly, another microporous Zn-MOF where the linker, 5-(2-(5-fluoro-2-hydroxyphenyl)-4,5-bis(4-fluorophenyl)-1H-imidazol-1-yl)isophthalic acid, experiences an ESIPT reaction, was demonstrated to be an outstanding candidate for the rapid detection of water owing to the enol-keto phototautomerism.^206^ Another very interesting example of the use of ESIPT linkers in the construction of MOFs is the one reported by Uribe-Romo and co-workers.^208^ The authors have designed ESIPT linkers with the capacity of emitting blue, green and red light, which were then utilized in different percentages to construct a Zr-based MOF (Figure 23). Interestingly, the MOF retained the emission of the linkers, exhibiting multicolor and high-quality white light emission.

Further to the very few reports we exemplified above, and to the best of our knowledge, there are no examples of ESPT molecules encapsulated in MOFs, so this area presents a hugely unexplored field in the context of LG@MOF designs. Some of the big questions that need to be investigated on this regard will be as follows. (1) Would it be possible to encapsulate ESIPT molecules within the MOFs while retaining the intramolecular proton transfer reaction? (2) Would it be possible to manipulate, control, and tune intermolecular ESPT reactions between a proton-transfer guest and a MOF containing functionalized linkers (e.g., with −CN, −SH, or −NH_2_)? (3) Would it be feasible to leverage the novel ESPT@MOF materials to develop luminescent sensors, or to yield other photonic devices?

### 5.4 Confinement Effects, Weak Interactions, and Aggregates Formation

The encapsulation of LGs in the pores of the MOFs may induce alterations of their structure and photophysical properties. On the one side, it is well-known that many organic fluorophores are sensitive to the nature of the surrounding environments, such as polarity or acidity/basicity, usually leading to bathochromic (red) or hypsochromic (blue) shifts depending on the characteristics of the guest materials. A typical example of these type of organic dyes are the coumarins. For instance, while the emission maximum of the coumarin 460 (C460) in methanol (or ethanol) is at ∼450 nm,^210^ it has been demonstrated that it could shift to 464 or 430 nm upon its encapsulation into Eu_0.05_Tb_0.95_BPT or Tb-TATAB MOFs, respectively.^76,77^ Similarly, the emission maximum of coumarin 343 (C343) slightly shifts from 482 nm in methanol,^210^ to 475 nm upon its encapsulation in a Hf-MOL.^87^ Highly distinct is the red-shift of 30 nm (1250 cm^–1^) observed in the emission spectrum of C153 upon its encapsulation in a Zr-NDC MOF.^85^

On the other hand, the restriction imposed on the fluorophores by their entrapment or caging into the MOFs–known as the confinement effect–generally decreases or even suppresses the nonradiative processes (e.g., molecular motions, that deactivate the fluorophore in the form of heat), turning into an augmentation of the emission quantum yield and lifetime. For instance, the encapsulation of a BODIPY dye, PM605, in the restricting pores of ZIF-8 decreases the nonradiative processes undergoing in the photoexcited PM605, resulting in an increase of the average lifetime from 6.8 ns (acetonitrile solution) to 7.5 ns.^211^ Similarly, the confinement of the metal complex ZnQ in the OX-1 MOF structure, enhances the luminescence quantum yield (from Φ = 35% in DMF suspension to 44% in the MOF) and extends the lifetime of the fluorophore.^35^ Another typical example of confinement effect has been described for the metal–organic complex Ru(II)tris(2,2′-bipyridine) (Rubpy) after its encapsulation into different MOFs.^212−214^ In all these examples, the caging of the Rubpy by the MOF host prevents its expansion, increasing the energy barrier to populate the ^3^LF state, suppressing the major nonradiative deactivation pathway, and therefore, resulting in a rise of the emission lifetime.^212−214^ This confinement effect has also been reported for Cu nanoclusters incarcerated into ZIF-8, where it was observed a 20-fold increase of the luminescence quantum yield (from 0.5% to 11%) accompanied by a substantial rise of emission lifetime (from 1.3 to 11 μs).^132^ Surprisingly, the role of the confinement effect has been neglected in many other reports, where the increment of the emission lifetime of the encapsulated guests is solely attributed to an energy transfer process, while it was not considered as a possibility that the guest entrapment or caging phenomenon may contribute even more importantly to the rise of the emission lifetime value.^98,138,139,215^

In addition to the aforementioned effects, the encapsulation of increasing concentrations of LGs into MOFs may give rise to the formation of guest aggregates (H- or J-aggregates), which generally shift the emission to the reddest part of the spectral region (owing to the presence of J-aggregates), and could decrease the emission intensity if the concentration becomes too high. Therefore, the potential applicability of LG@MOF materials in photonic technologies will strongly depend on a proper control and careful manipulation of the amount of encapsulated guest loading within the MOF host. Excessive amount of guest will generally lead to the formation of aggregates, while insufficient concentration of LGs will prevent a high luminescence intensity. Thus, for certain purposes, it is necessary to prepare different LG@MOF composites, with increasing amounts of LGs to estimate the optimal concentration, where the luminescence intensity is the highest while the concentration of aggregates is minimal but still present.

Even though the observation of a red-shift in the emission spectrum with an increasing amount of organic dyes confined in MOFs is a well-known observation in many studies,^74,78,136^ in general, there is a lack of information or the phenomenon is vaguely explained.^74,75,78,136,138,139,142^ In fact, some reports attribute this effect to aggregation, autoabsorption (reabsorption) or intermolecular interactions favored by the incorporation of fluorophore dyes in the MOF host.^75,138,139,142^ Although all these processes may induce a red-shift of the emission spectrum, there are some experiments that could assist in unravelling the formation of the J- and H-aggregates. First, it is well-known that the S_0_ → S_1_ transition of H-aggregates is more energetic than the monomers, while that of the J-aggregates is less energetic. This means that the absorption spectrum will be broadened upon the formation of the aggregated species, and a proper deconvolution of the bands could provide information about the formation of those species.^43^ Additionally, a detailed time-resolved photophysical characterization of the LG@MOF composites will be essential to determine the presence of those aggregates. According to the Kasha’s excitonic theory, the S_1_ → S_0_ is a forbidden transition for the H-aggregates, while in the case of the J-aggregates it will be less energetic than for the monomers.^216,217^ Considering the emission lifetimes and contributions, apart from the emission lifetime of the monomers, it is plausible to get two additional time components. One of them will be a very short emission lifetime (τ_1_) that can be attributed to the H-aggregates, as its relaxation is a forbidden transition. The second additional component (τ_2_) whose amplitude (or contribution) will rise to the reddest part of the emission spectrum will correspond to the lifetime of the J-aggregates, as these will emit in the lower energy region.

**Figure 24:**
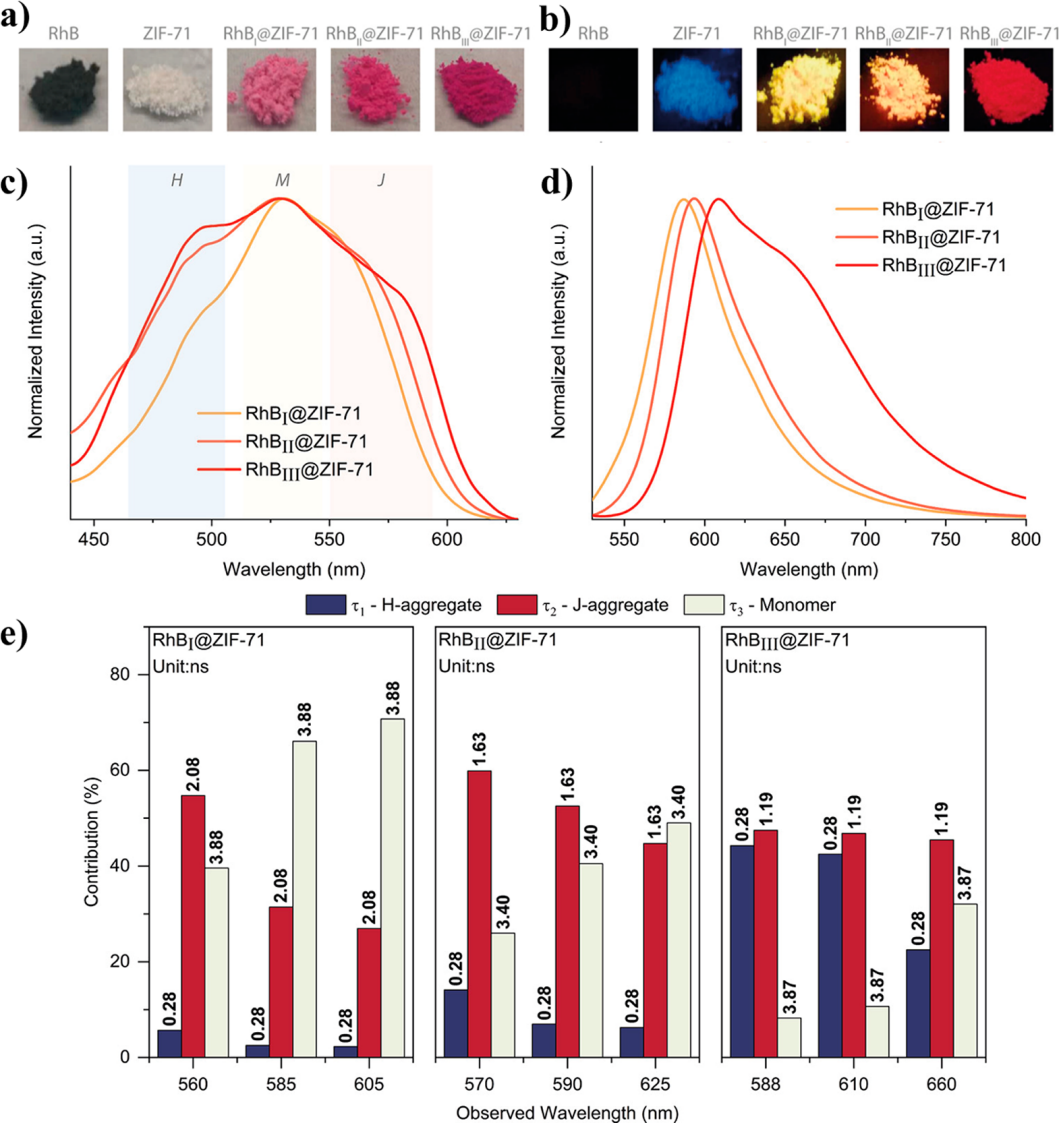
Photographs of the RhB, ZIF-71, and RhB@ZIF-71 composites with different RhB concentrations observed under (a) daylight and (b) UV excitation (365 nm). (c) Normalized excitation spectra (λ_em_ = 650 nm) and (d) normalized emission spectra (λ_ex_ = 515 nm) of the different RhB@ZIF-71 composites. (e) Time-resolved data of RhB@ZIF-71 obtained using three different RhB concentrations, showing the lifetimes and contributions of the monomer, H- and J-aggregate species. Adapted from ref (42). Copyright 2020 American Chemical Society.

The foregoing observations have been well demonstrated for a series of RhB@ZIF-71 composite materials.^43^ In this study, Zhang et al. prepared 3 sample batches with an increasing concentration of the RhB dye. First, the emission spectra were red-shifted with the concentration, while the excitation spectra showed a broadening, reflecting the possible formation of both the H- and J-aggregates (Figure 24). Then the authors carefully investigated the time-resolved photodynamics of those materials. In all the samples, it was reported a multiexponential decaying behavior, giving the three time components of: τ_1_ = 0.28 ns, τ_2_ = 1.2–2.1 ns, and τ_3_ = 3.4–3.9 ns. The shortest time component (τ_1_) was assigned to the H-aggregates, the intermediate component (τ_2_) attributed to the J-aggregates, while the longest component (τ_3_) was the emission lifetime of the monomers.^43^ Clearly, the contribution of the τ_1_ and τ_2_ components (H- and J-aggregates, respectively) increased for the composites with a higher concentration of RhB guests, hence unequivocally proving the formation of a growing population of aggregates upon rising the amount of dyes in the ZIF-71 MOF. Very similar results were also reported after the encapsulation of an increasing amount of NR dye in the Al-ITQ-HB MOF.^144^ In addition to the expected red-shift of the emission spectra, the authors reported a multiexponential photobehavior with three lifetimes of: τ_1_ = 0.71–0.26 ns, τ_2_ = 2.59–1.23 ns, and τ_3_ = 5.1–3.15 ns, assigned to the emission lifetime of the H-aggregates, J-aggregates, and NR monomers, respectively.^144^

A similar multiexponential behavior was also reported for the fluorescein@ZIF-8 system. At lower guest concentrations, the authors reported a biexponential decay owing to the emission of the well isolated monomers and J-aggregates, while at higher guest concentrations, the decays become multiexponential with three lifetimes of τ_1_ = 1.1 ns, τ_2_ = 3–3.7 ns, and τ_3_ = 6.5 ns, attributed to the emission lifetime of the H-aggregates, J-aggregates, and monomers, respectively.^83^ These reports are clear examples of how a detailed photodynamic characterization of LG@MOFs is essential for the control and manipulation of the composite’s luminescent behavior, and therefore, enabling fabrication of bespoke composite systems with precisely engineered properties.

## 6 Potential Applications and Devices Integrating LG@MOFs

**Figure 25:**
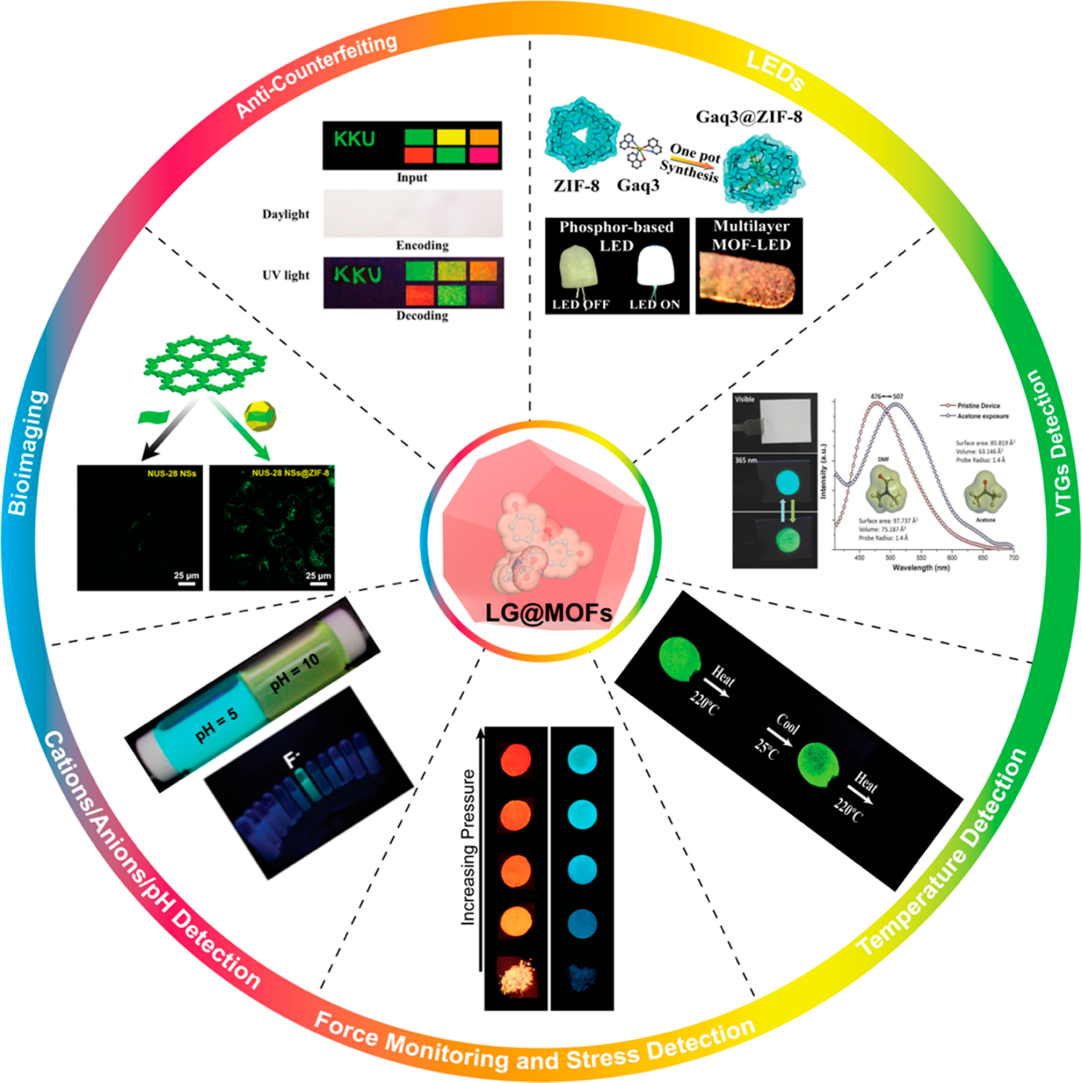
Emergent applications and potential uses of novel LG@MOF materials by harnessing their vast photophysical and photochemical properties to yield smart optical sensors, photo switches, and stimuli-responsive photonics. VTG = volatile organic compounds, toxic chemicals, and gases. Inset figure for LEDs adapted from ref (37). Copyright 2020 John Wiley and Sons. Inset figure for VTG detection adapted from ref (35). Copyright 2017 John Wiley and Sons. Inset figure for temperature detection adapted from ref (225). Copyright 2020 Elsevier. Inset figure for force monitoring and stress detection adapted from ref (43) (Copyright 2020 American Chemical Society) and ref (240). Copyright 2022 Elsevier. Inset figure for anion sensing adapted from ref (243). Copyright 2018 American Chemical Society. Inset figure for bioimaging adapted from ref (147). Copyright 2019 American Chemical Society. Inset figure for anticounterfeiting adapted from ref (112). Copyright 2019 American Chemical Society.

Because of their vastly tailorable luminescent properties and stimuli-responsive behavior, the LG@MOF systems have the potential to be employed in new innovative technological applications, as summarized in Figure 25. In this section, we shall focus the discussion on emerging photonic applications in the fields of chemical, temperature and pressure sensing, energy conversion and solid-state lighting. In the vibrant field of MOF sensors,^10,19,218,219^ access to bespoke LG@MOF systems will offer an innovative platform for accomplishing unconventional host–guest interactions via nanoscale confinement effect. Exceptionally, one should expect to see a growing number of new sensing applications or enhanced device performance emerging from the development of LG@MOF composites.

Currently, the reported sensors incorporating LG@MOF materials can be roughly divided into two categories: the “turn-off” type and the “turn-on” type. When encountering a target condition or external stimulus (e.g., temperature, stress, pH, charge), the turn-off type sensor responds by decreasing its luminescent intensity, whereas the turn-on type sensor responds to the stimuli by increasing its luminescence. It is projected that fluorescent turn-on sensing holds the key to the future sensors development,^220,221^ because various environmental factors, such as water moisture and/or photodegradation, may also diminish the luminescent intensity of LG@MOF, thereby interfering with the signal output from turn-off type sensors. Further to the luminescent intensity changes, some of the turn-off and turn-on sensors constructed from LG@MOF could demonstrate a peak wavelength shift or exhibit a dual emission. The change of peak wavelength gives an intuitive visual output that is easily perceptible to the naked eye, while the dual-emission behavior can be harnessed for engineering a self-calibrating device for mitigating any unwanted effects caused by external interferences.

### 6.1 Volatile Organic Compounds, Toxic Chemicals, and Gases (VTGs) Detection

Industrial activities and the daily life of modern society will inevitably use and emit a lot of volatile organic compounds (VOCs), toxic chemicals and gases (VTGs). With the rapid urbanization around the world, the negative impact of VTGs on the environment is becoming more pronounced,^8^ and concomitantly, the demand for high-performance VTG sensors also increases for hazard detection and risk mitigation.

**Figure 26:**
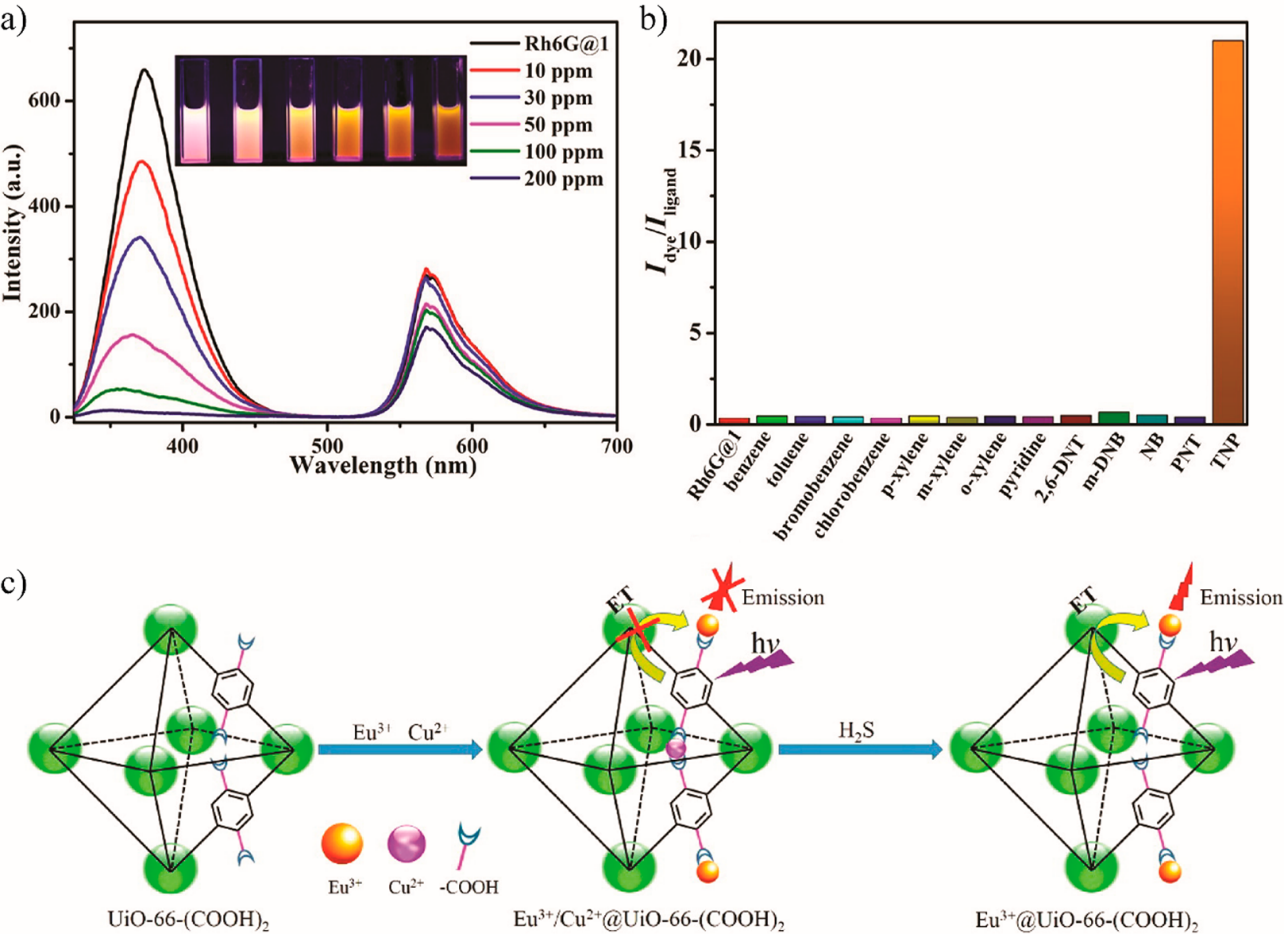
(a) Emission spectra for Rh6G@MOF at different concentrations of TNP [MOF = [(NH_2_Me_2_)[Zn_3_(μ_3_–OH)(tpt)(TZB)_3_](DMF)_12_]_n_]. (b) Peak-height ratio of dye to ligand after addition of 200 ppm of various analytes. Adapted with permission from ref (223). Copyright 2017 American Chemical Society. (c) Schematic illustration of the fluorescence detection mechanism of Eu^3+^/Cu^2+^@UiO-66-(COOH)_2_. Adapted with permission from ref (224). Copyright 2016 American Chemical Society.

Among the many potential VTG-sensing materials being developed, the LG@MOF system appears as one of the most promising in the field. For example, the recently developed RhB@ZIF-71 crystals exhibit fluorochromic VOC detection ability.^43^ It was reported that when RhB@ZIF-71 was exposed to certain VOCs, especially N,N-dimethylformamide (DMF), its luminescence becomes significantly quenched because the encapsulated RhB guest transformed to nonfluorescent lactone in a polar aprotic solvent. While a typical turn-off type sensing behavior has been shown here, this material is still far from making an actual VOC sensor since it will need better selectivity and inertness from environmental factors to afford practical use. Therefore, research based on the dual-emission LG@MOF system is particularly relevant, whose self-calibration property can be exploited to eliminate external interference through a ratiometric sensing approach. For example, Chen et al.^222^ have developed a Rh6G@MOF system (rhodamine 6G@[(NH_2_Me_2_)[Zn_3_(μ_3_–OH)(tpt)(TZB)_3_](DMF)_12_]_n_, tpt = 2,4,6-tri(4-pyridyl)-1,3,5-triazine, H_2_TZB = 4-(1H-tetrazol-5-yl)benzoic acid). This material exhibits two emission peaks originating from the tpt ligand (363 nm) and Rh6G (580 nm), respectively. When exposed to 2,4,6-trinitrophenol (TNP, a toxic pollutant and highly explosive molecule), the luminescence from the ligand becomes significantly quenched, while the response of Rh6G was only minimally affected. By tracking the intensity ratios derived from the two emission peaks, the TNP molecules can be detected effectively (Figure 26a). Furthermore, the authors found that the Rh6G@MOF could selectively detect TNP among many non- and most nitroaromatic compounds (Figure 26b). Prior to this study, all the MOF-based TNP sensors reported were simply the type-off type, this work therefore marks a significant development in the field by demonstrating the promise for building a ratiometric fluorescent sensor by leveraging the LG@MOF concept. In the same way, Yin et al.^223^ reported a Ru@MIL-NH_2_ system capable of ratiometric fluorescence detection of water in organic solvents. This work demonstrates the dual emission characteristics having a relatively fast response time of ∼1 min, further highlighting the potential of the LG@MOF concept.

The turn-on type response of LG@MOF sensors used to detect VTGs is relatively rare; thus far, this requires Ln-based materials. For example, Zhang et al. reported a Eu^3+^/Cu^2+^@UiO-66-(COOH)_2_ system showing turn-on and ratiometric sensing for H_2_S.^224^ When not in contact with the target H_2_S molecules, the system shows dual-emission characteristics of both Eu^3+^ and the ligand, and there was an energy transfer from the ligand (H_4_btec = 1,2,4,5-benzenetetracarboxylic acid) to the Eu^3+^ ions, this phenomenon is called the antenna effect, see the mechanism depicted in Figure 26c. The Cu^2+^ in the system could interact with the Lewis basic carboxylic oxygen sites to form O–Cu–O through the cooperative effect, and this interaction will restrain the antenna effect, causing a weak Eu^3+^ and a strong ligand emission. When the system is exposed to sulfide, Cu^2+^ (a soft acid), would react with the sulfide (a soft base), destroying the O–Cu–O structure. Therefore, the greater antenna effect leads to rise in the Eu^3+^ luminescence but a decline of emission intensity associated with the ligand. This type of Ln@MOF system can retain the intrinsic properties of encapsulated Ln^3+^, and may help to overcome the design and synthesis difficulties due to the higher coordination numbers and the more variable nature of the Ln^3+^ coordination sphere commonly encountered in the more conventional Ln-based MOFs.^53^

### 6.2 Temperature Sensing and Non-Invasive Thermometry

The temperature sensor is one of the most commonly used sensors in our daily life, and it can be roughly divided into the contact and noncontact type. However, these two types cannot meet all the practical engineering requirements. Contact type temperature sensors must be put in contact with the target to acquire a reading. While noncontact types, such as infrared (IR) thermometers, can be easily affected by the environment, and they are neither suitable for measuring fine-scale microscopic objects nor for measuring the temperature of the interior of objects.

To address these shortcomings, some researchers have turned their attention to luminescent thermometers. Simply put, one can place the fluorescent thermochromic material in the vicinity of the target, which will noninvasively measure the temperature of the surrounding by detecting the change in its luminescent behavior.^225^ Because of the various forms and nano size of luminescent materials, this enables one to perform challenging temperature measurements in unconventional settings.^12^

The LG@MOF is a good system for making luminescent temperature sensors, but most of LG@MOF-based sensors are the turn-off type. That is because the increase in temperature will cause the nonradiative decay of the emitter to increase, leading to a decrease in the overall luminescent intensity. For example, Hu et al.^28^ used a MOF host, [(CH_3_)_2_NH_2_]^+^[Zn_4_(μ_4_-O)(NTB)_2_(NO_2_-bdc)_0.5_]·3DMA (NTB = 4,4′,4′′-nitrilotrisbenzoic acid, NO_2_-bdc = 2-nitro-4-benzenedicarboxylic acid), to encapsulate RhB guest and obtain a RhB@MOF system. This system exhibits the characteristics of traditional RhB-based temperature sensing, where its luminescent intensity decreases as a function of temperature rise.

**Figure 27:**
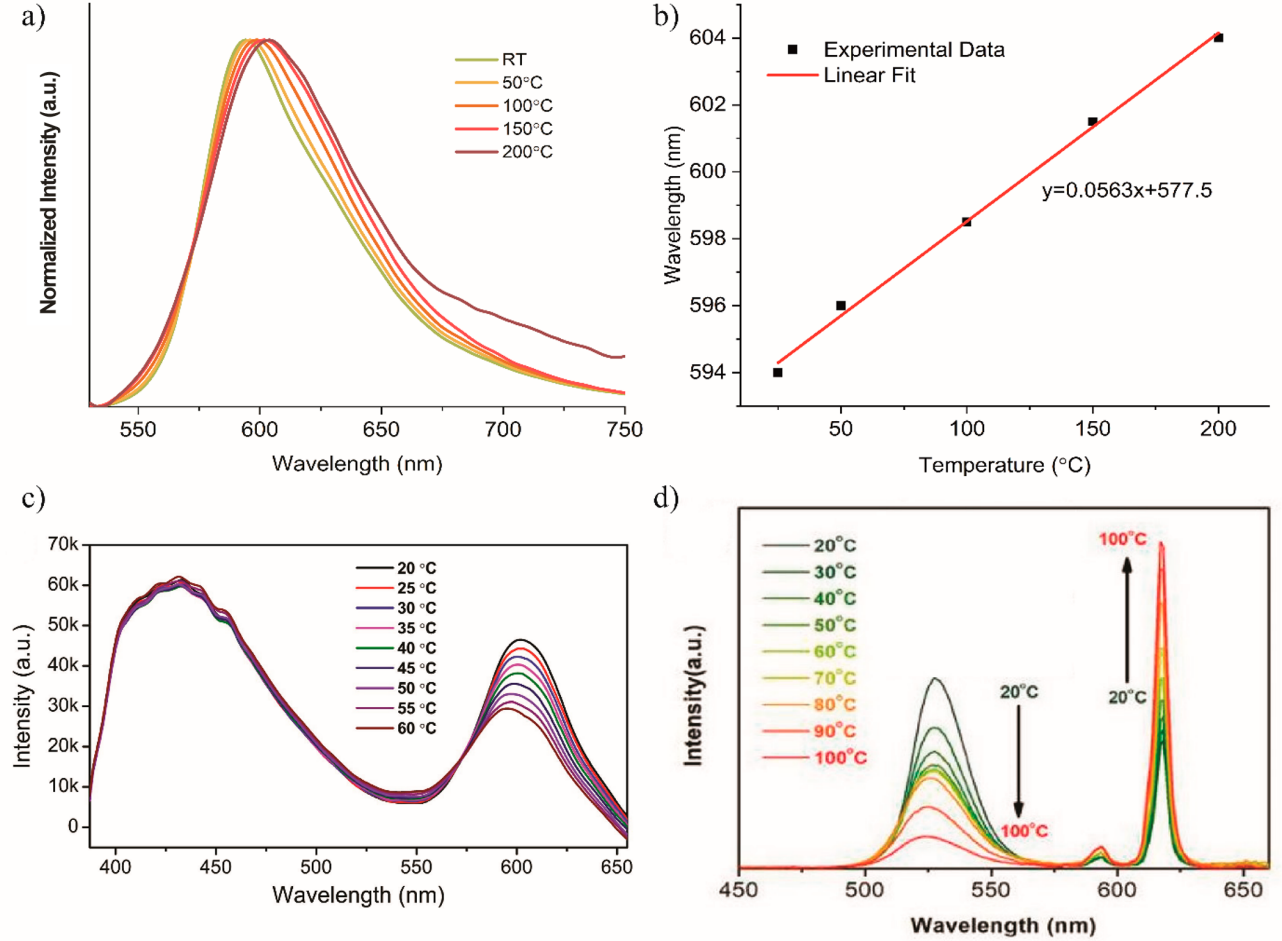
(a) Normalized emission spectra of RhB@ZIF-71 when temperature rises from room temperature to 200 °C. (b) Linear relationship of the emission peak wavelength of RhB@ZIF-71 as a function of temperature. Adapted from ref (43). Copyright 2020 American Chemical Society. (c) Temperature-dependent photoluminescence emission spectra of Rh101@UiO-67. Adapted with permission from ref (230). Copyright 2018 Elsevier. (d) Temperature-dependent photoluminescent spectra of CsPbBr3@Eu-BTC in the temperature range of 20–100 °C. Adapted with permission from ref (121). Copyright 2020 American Chemical Society.

Further to retaining the temperature sensitivity of the luminescent guest itself, more importantly, the LG@MOF may impart the ability of peak wavelength shift on the fluorophore because of the pore confinement effect (Section 5.4). For example, the RhB@ZIF-71^43^ also exhibits a turn-off thermochromic response. Compared with pure RhB, RhB@ZIF-71 exhibits a red-shift of its emission spectrum with an intensity that decreases linearly as a function of temperature as shown in Figure 27a and b. That is because the MOF pore imposes different caging effects on the RhB monomers, J-aggregates, and H-aggregates. The J-aggregates are relatively larger and receive a stronger protection due to pore confinement. Therefore, when temperature increases, the relative luminescent intensity of the J-aggregates decreases less rapidly, and this effect produces a red-shift of the overall emission. In contrast, all other RhB-based temperature sensors reported to date could only show the common decline in their emission intensity,^226,227^ without demonstrating a peak shift observed in the RhB@ZIF-71 system.^43^ The above finding shows that the guest–host interaction of LG@MOF system can be harnessed to broaden the potential applications of conventional fluorophores to accomplish unconventional temperature sensing capabilities.

The LG@MOF system can bestow unique sensing ability onto some luminescent guests or fluorophores which, in the pristine form, do not exhibit any temperature sensing properties. For example, the rhodamine 101 (Rh101) dye is a thermally insensitive material, due to its structural rigidity.^228,229^ Zhou et al.^230^ reported a Rh101@UiO-67 system, which has both Rh101 and UiO-67 luminescence, but the emission from the former reduces with increasing temperature as shown in Figure 27c. The authors proposed that this phenomenon was related to the back energy transfer from the dye molecules to the linkers and the increase in temperature would increase this effect, causing the intensity to decline in Rh101. Remarkably, because the emission from the UiO-67 host stays unchanged with temperature, the system becomes as a self-calibrating sensor.

There are some lanthanide-based LG@MOFs that can perform turn-on temperature sensing. Unlike pure Ln-MOFs, it is possible to introduce a turn-off type LG into them, which will yield a self-calibrating system and improve the temperature resolution. For example, Liu et al.^121^ encapsulated a perovskite CsPbBr_3_ into an Ln-MOF (Eu-BTC) host and the obtained CsPbBr_3_C@Eu-BTC exhibits both the characteristic emission peaks of Eu^3+^ and CsPbBr_3_. With the increase in temperature, as shown in Figure 27d the emission of Eu^3+^ rises and that of CsPbBr_3_ falls, resulting in a turn-on type response jointly with self-calibrating feature. The authors claimed that although Eu^3+^ in other composites had a very low-temperature response due to the weak coupling effect, the high luminescent efficiency of CsPbBr_3_ and the confinement effect of Eu-BTC host significantly improved the temperature sensing performance of Eu^3+^.^121^ This improvement once again illustrates the potential benefits for deploying bespoke LG@MOF composite systems.

### 6.3 Mechanochromic Sensors for Force Monitoring and Stress Detection

Fluorescent mechanochromic sensors have attracted much attention because of their potential implementation in mechanical stress/strain-based adaptive devices and for the visual detection of material failure. At the core of the mechanofluorochromic sensor is a fluorescent sensing material that changes its luminescent response as a function of the imposed force (stress), deformation (strain), or pressure. Particularly, there are plentiful reports available on the mechanofluorochromic behavior of polymeric systems, organic compounds, and metal complexes.^231,232^ Conversely, studies on mechanochromic MOFs are relatively limited,^233−237^ and fewer still if based on the concept of mechanochromic Guest@MOF systems.^38,43^

The typical mechanisms that control the emission of a mechanochromic material is the mechanically induced deformation of the structure to yield excimer formation,^238^ phase transformation under stress, or via aggregation induced emission (AIE) under pressure.^239^ It has been proposed that high-efficiency mechanochromic materials are more likely to be obtained when the mechanism is driven by structural changes from physical means rather than by chemical means.^231^ Thus, the physical caging of fluorophores by long-range nanoconfinement in LG@MOF system makes it an ideal candidate for achieving mechanochromism. For example, the perylene@ZIF-8 system reported by Chaudhari et al.^38^ shows a reversible mechanochromic response with an emission wavelength shift. When this material was not stressed, under UV, it emits in blue (442 nm) but after compression it switches to a green emission (502 nm). Subsequently, the initial blue emission can be regained when the compressed pellet was ground into a powder form to release residual stress.

**Figure 28:**
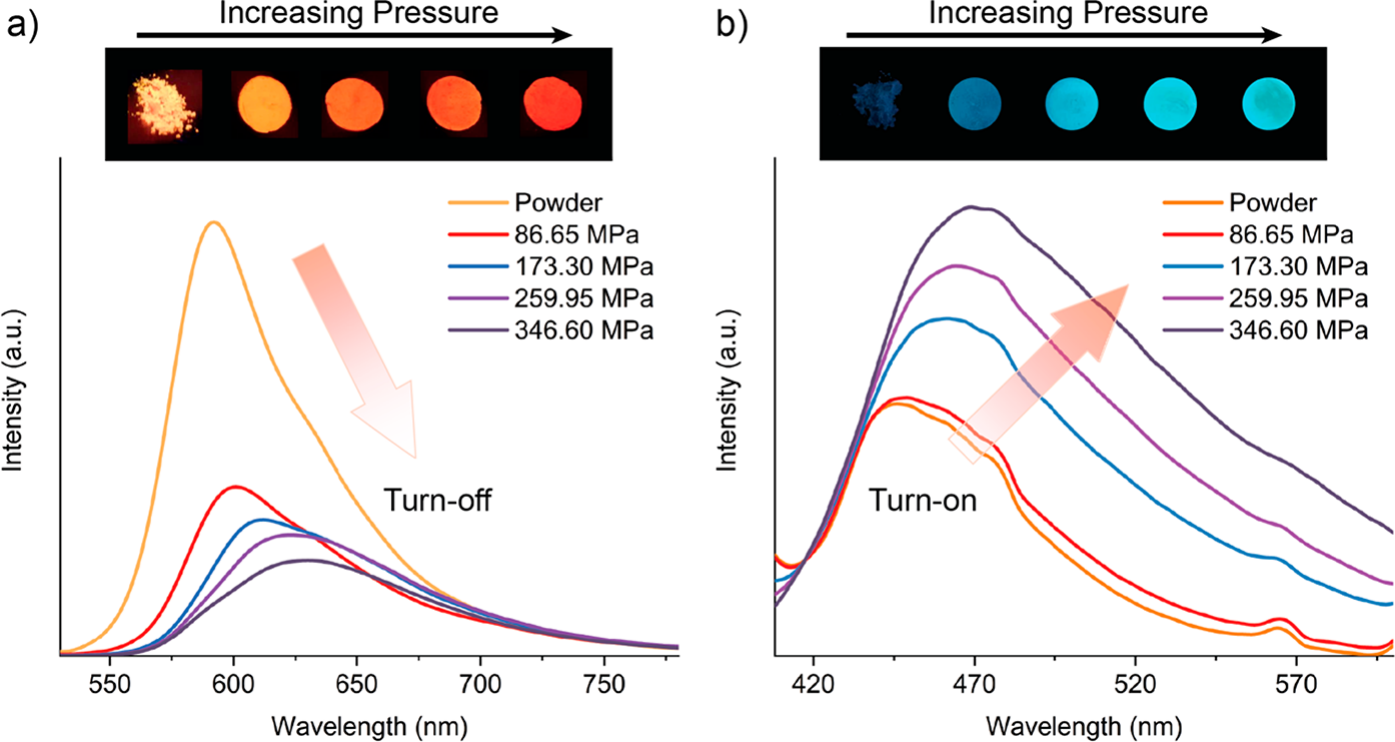
Emission spectra showing the turn-off and turn-on type mechanofluorochromic response of (a) RhB@ZIF-71. Adapted from ref (43). Copyright 2020 American Chemical Society. and (b) TPE@ZIF-71, respectively, when samples were subjected the effect of pelleting pressure. The diameter of the pellet is ∼10 mm. Adapted from ref (240). Copyright 2022 Elsevier.

Another example concerns the RhB@ZIF-71 system that exhibits a turn-off mechanochromic effect, see Figure 28a.^43^ This mechanism involves not only the enhanced π–π interaction, but it has been hypothesized that an increasing pressure generates a tighter formation of J-aggregates coupled with the switching of H-to-J aggregates within the pores, resulting in a red-shifted emission.

Leveraging the nanoconfinement effect conferred by MOF structures, recently it has been shown that the turn-on type mechanochromism of LG@MOF can also be accomplished. The study concerns the TPE@ZIF-71 system (TPE = tetraphenylethylene; an AIE molecule), where the vibrations of the TPE guest molecules were found to be restricted when confined within ZIF-71 pores and under pressure, this mechanism generates a turn-on mechanochromic response (Figure 28b).^240^ It must be emphasized that pristine (unconfined) RhB, perylene, and TPE molecules are not mechanochromic by themselves under the same test conditions. The foregoing exemplars support the notion that the ordered framework structure of the MOF host could help to direct the mechanical response of the LG@MOF system. Significantly, the physical structural changes undergone by LG@MOFs subject to mechanical deformation or pressure can be translated into an efficient mechanofluorochromic response, potentially yielding both kinds of turn-on/-off mechanosensors by design.

### 6.4 Cations and Anions Sensing, Optical pH Detection

**Figure 29:**
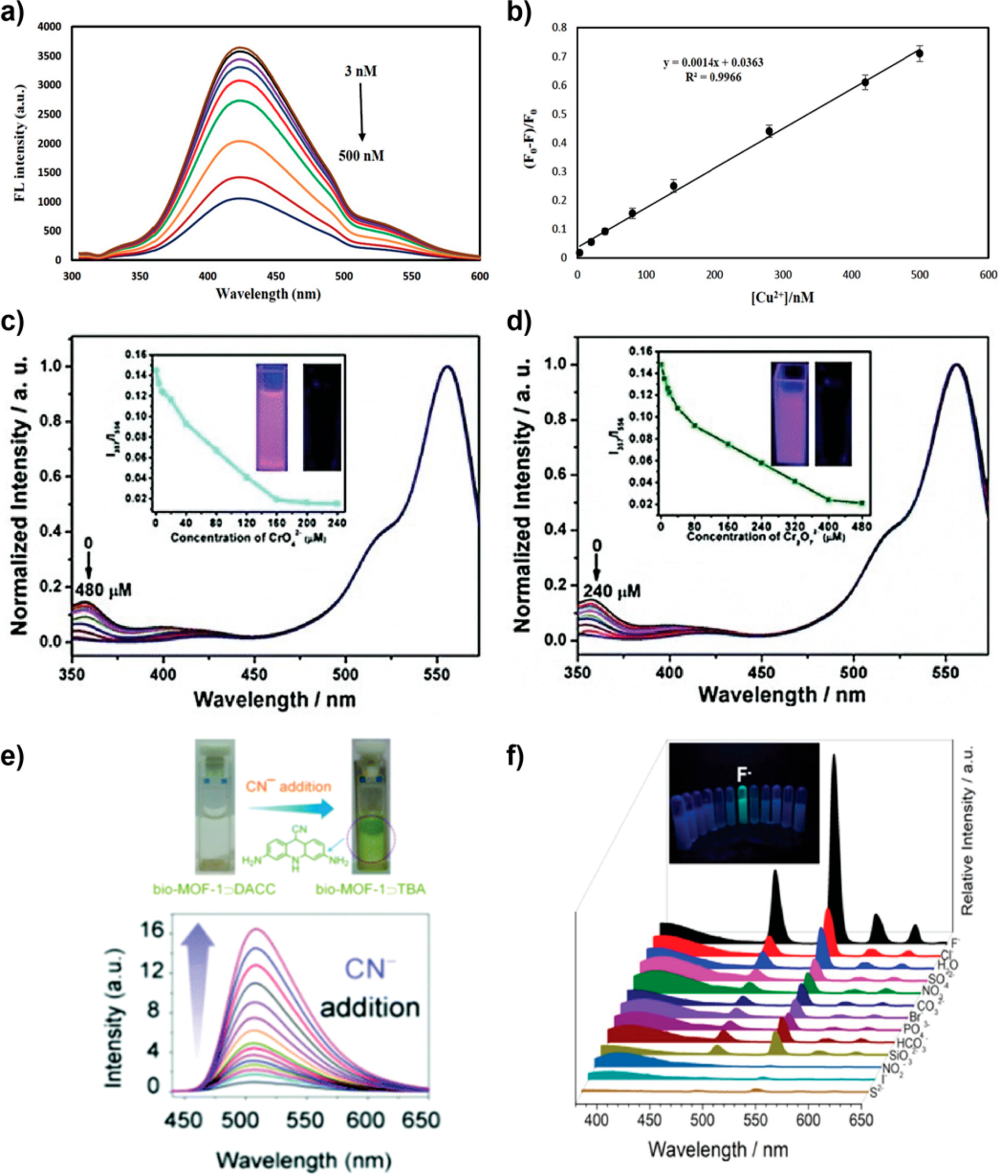
(a) Fluorescence spectra of PEG-ZnS QD@ZIF-67 in the presence of a series of different concentrations of Cu^2+^ ions from 3 to 500 nM. (b) Linear relationship between the fluorescent intensity and Cu^2+^ concentrations from 3 to 500 nM. Adapted with permission from ref (34). Copyright 2019 Elsevier. (c, d) Fluorescence excitation spectral changes of RhB@ZIF-90 with the addition of (c) CrO_4_^2–^ and (d) Cr_2_O_7_^2–^. Adapted with permission from ref (241). Copyright 2018 Royal Society of Chemistry. (e) Visual change in the supernatant solution upon addition of cyanide ions to DAAC@bio-MOF-1 and changes in fluorescence intensity via a turn-on response upon addition of CN^–^ ions to DAAC@bio-MOF-1. Adapted with permission from ref (242). Copyright 2017 Royal Society of Chemistry. (f) Emission spectra of Tb^3+^@Zr-MOF dispersed in different anion solutions. Adapted with permission from ref (243). Copyright 2018 American Chemical Society.

The research on cation/anion sensing is one of the most popular directions in the luminescent MOF field, but most studies are related to the turn-off type. Generally, one can directly harness the properties of the guest itself in the LG@MOF system to attain sensing behavior. For example, Asadi et al.^34^ developed a core–shell LG@MOF system, comprising polyethylene glycol (PEG)-capped ZnS QDs@ZIF-67. This system showed sensitivity to Cu^2+^ detection. The mechanism involves the oxygen atoms on the hydroxyl groups of PEG-ZnS itself, which form a complex with Cu^2+^ thus quenching the luminescence of PEG-ZnS. Although this mechanism is not altered by MOF confinement, compared with the pure PEG-ZnS (unencapsulated), it was found that the accumulation effect in the ZIF-67 pores further boost the sensitivity and selectivity toward the Cu^2+^ analyte (Figure 29a,b). Notably, research to date has shown that even if the MOF host is not directly involved in the sensing process, it still can play an important role by enhancing the ion sensing ability of the encapsulated guest, thereby raising the performance of the LG@MOF system as a whole.

Many studies have indeed made full use of the dual-emission properties of LG@MOF to fabricate sensors with self-calibrating capability for the detection of cations and anions. For example, Jin et al.^241^ encapsulated RhB into the nanosized crystals of ZIF-90 and the obtained RhB@ZIF-90 system exhibited ratiometric fluorescent sensing to Cr(VI) anions (Figure 29c,d). This is because the UV–vis absorption of Cr(VI) anions such as CrO_4_^2–^and Cr_2_O_7_^2–^ covered most of the excitation spectrum of ZIF-90, and that inhibited the excitation of ZIF-90, while the excitation of RhB was not influenced by the Cr(VI) anions. In this way, when exposed to Cr(VI) anions, the excitation peak of ZIF-90 decreased, but that of RhB remained unchanged, thus yielding the self-calibration property.

Compared with the VTGs, temperature, and mechanochromic sensors discussed above, there are relatively more LG@MOF turn-on sensors for cations or anions. We can roughly divide this part of turn-on type sensors into 2 categories according to whether Ln^3+^ is used in the system or not. In principle, LG@MOF systems comprising ordinary (non-Ln^3+^) luminescent guests usually use MOF to suppress the luminescence of the guest, such that only when the target ion comes into contact, the guest will reactivate its own emission to achieve turn-on sensing. For example, the DAAC (3,6-diaminoacridinium cation)@bio-MOF-1 reported by Karmakar et al. has demonstrated the turn-on sensing of cyanide ions (CN^–^), see Figure 29e.^242^ In terms of its mechanism, the emission of DAAC was initially being inhibited by bio-MOF-1 in the guest–host system, and when it came into contact with CN^–^, the CN^–^ would attack the C9 carbon of DAAC molecules to form a covalent bond. The formed DAAC–CN was neutral and had good luminescent properties, so it would escape from the host framework and induce turn-on sensing. The principle of deploying a MOF host to suppress the luminescence of the confined guest is innovative, further research in this direction is warranted.

The second category of turn-on type cation/anion sensors being studied is based on the Ln^3+^ ions. In that case, the energy transfer/antenna effect is usually key for controlling the sensing performance of Ln@MOF. For instance, Zheng et al.^243^ introduced Tb^3+^ into a Zr-MOF to obtain the Tb^3+^@Zr-MOF system, whose luminescent properties are greatly enhanced when exposed to fluoride ions in an aqueous solution (Figure 29f). This is because an obvious antenna effect is established between the ligand of Zr-MOF and Tb^3+^, but the antenna effect becomes deactivated when the system is in water. When F^–^ is present, it generates a Lewis acid–base interaction with the uncoordinated Zr^4+^ in the Zr-MOF structure, restoring the energy transfer from the ligand to Tb^3+^ and reactivating the turn-on sensing response. Similarly, Eu^3+^@UiO-66(Zr)-(COOH)_2_ developed by Han and Yan^244^ has the turn-on type sensing behavior to Cd^2+^ for the same mechanism elucidated above.

**Figure 30:**
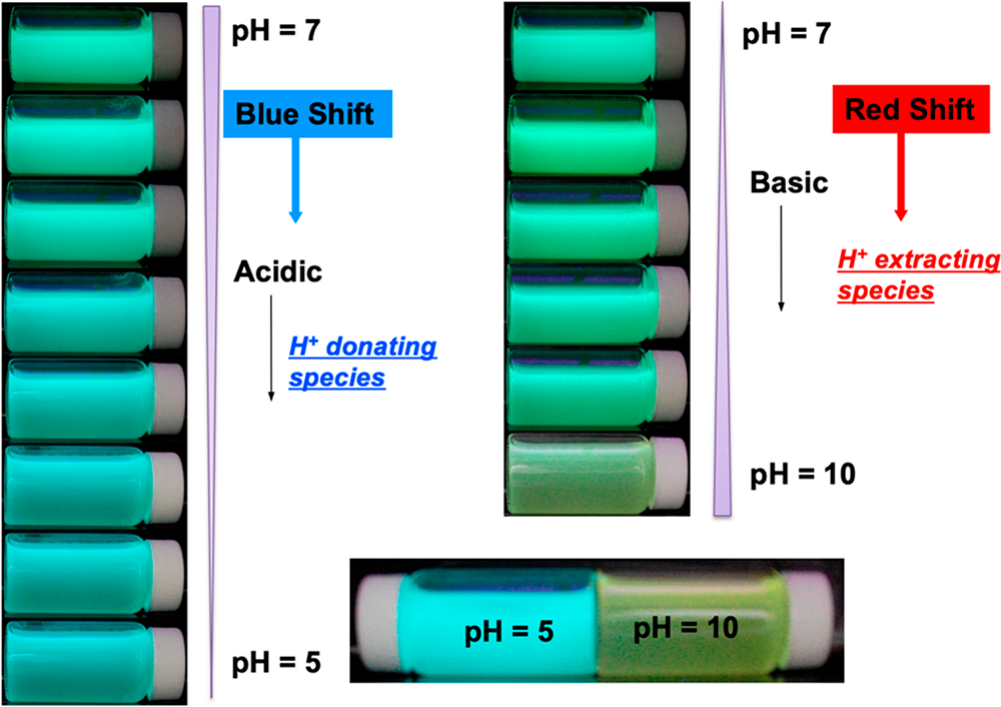
Optical pH sensing by utilizing the luminescent ZnQ@OX-1 nanosheets^35^ suspended in a set of analyte solutions whose pH varies systematically from 5 to 10.

LG@MOF materials can be developed for use as photoluminescent pH sensors. Figure 30 shows an exemplar where the dispersed ZnQ@OX-1 nanosheets^35^ are highly sensitive to the changing pH of the analyte solutions, within the tested range of pH = 1 to 10. When subject to an acidic environment, the emission of ZnQ@OX-1 nanosheets exhibits a blue shift in the presence of H^+^ donating species. Conversely, when exposed to a basic environment, the emission of ZnQ@OX-1 becomes gradually red-shifted in response to the presence of the H^+^ extracting species. The concept of optical pH response evidenced here can be ascribed to the donor/acceptor host–guest interactions conferred by nanoscale confinement. In fact, the pristine ZnQ metal complex, while it is luminescent, is not pH sensitive when not confined in the OX-1 MOF host. Nevertheless, caution is needed as the exposure to stronger acids (e.g., pH < 5 for ZnQ@OX-1) and bases may cause decomposition of the MOF structure leading to the loss of its pH sensing ability. In principle, adopting MOF hosts with improved acid-basic stability may be able to extend the operational range of such optical pH sensors for deployment in even harsher environments.

### 6.5 Bioimaging

**Figure 31:**
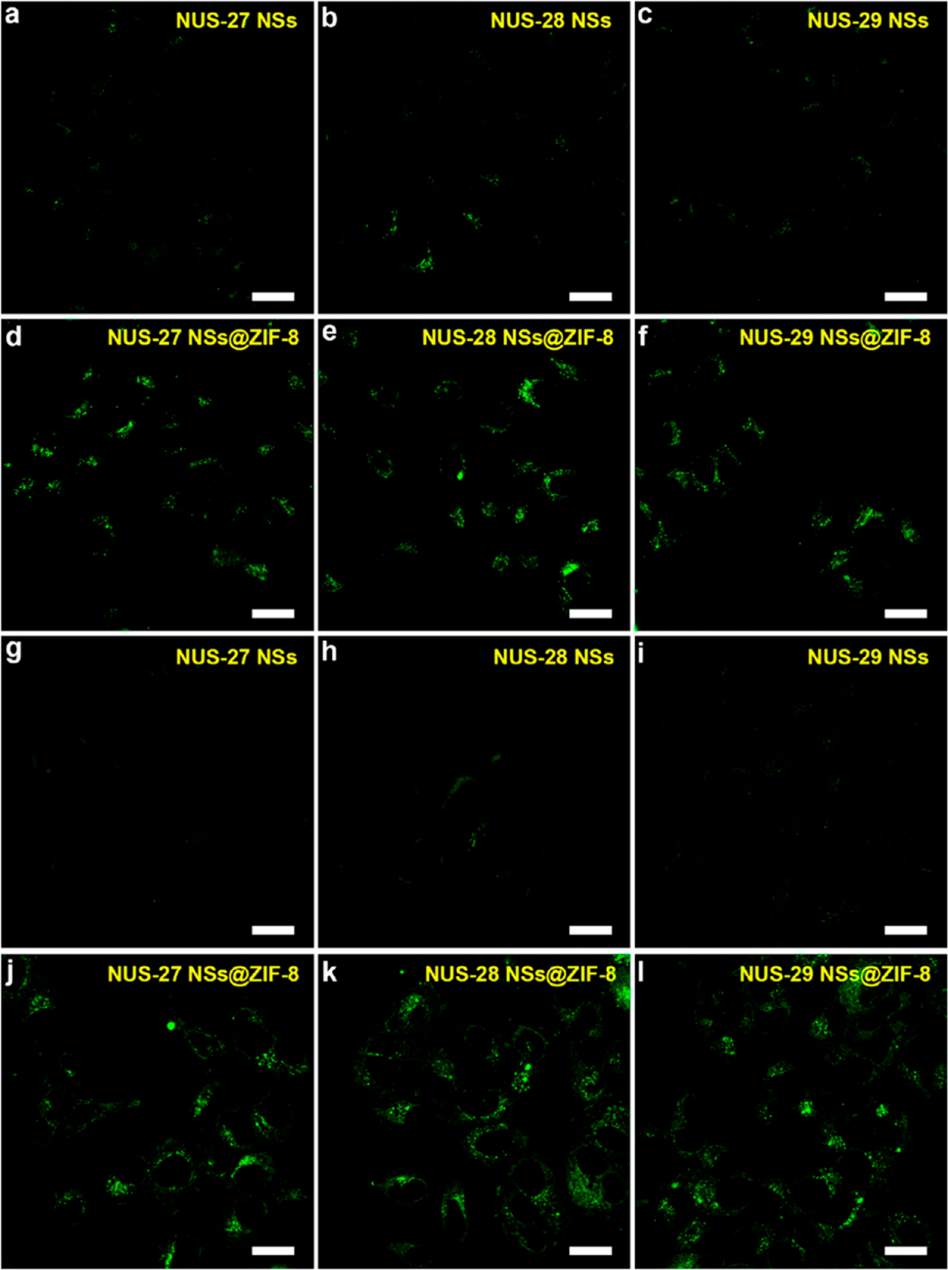
Confocal images of (a–f) HeLa and (g–l) MCF-7 cells using (a, g) NUS-27 nanosheets (NSs), (b, h) NUS-28 NSs, (c, i) NUS-29 NSs, (d, j) NUS-27 NSs@ZIF-8, (e, k) NUS-28 NSs@ZIF-8, and (f, l) NUS-29 NSs@ZIF-8, respectively. Adapted with permission from ref (145). Copyright 2019 American Chemical Society.

Bioimaging is a noninvasive method that allows the visualization of biological processes at real time, and thus, it has become an indispensable tool for monitoring biological phenomena and medical diagnosis.^245^ Therefore, there exists a great interest in the scientific community on developing new biomarker materials with tunable and improved abilities. Among all the proposed materials, LG@MOFs have emerged as a potential candidate for cell imaging, especially those in which the MOF has a nanometric size (nMOFs).^79,246,247^ Additionally, the numerous possible combinations of fluorescent guests and MOFs open an exciting avenue of research in this subject. Some outstanding MOF-based fluorescent probes have been reported for cell-imaging, they composed of a wide variety of luminescent guests, such as organic fluorophores,^79,248^ porous organic nanosheets,^145^ metal nanoclusters,^246,249^ and carbon dots.^247^ For example, the encapsulation of resorufin and rhodamine 6G in the nMOF-801 and nUiO-67 (Rs@nMOF-801 and R6G@nUiO-67), leads to the fabrication of LG@MOF materials that have been used for imaging the FL83B and HepG2 cells.^79^ Similarly, another LG@MOF material synthesized by the encapsulation of RhB into a hierarchical-pore Al-MOF has been successfully employed for imaging live MGC-803 cancer cells.^248^ In addition, the authors demonstrated the possible biodistribution of the RhB@Al-MOF in different organs, and found a major accumulation in the liver, stomach, lung and kidney, indicating that the material can be transported to all tissues by blood flow.^248^ Metal nanoclusters (NCs), like Ag or Au NCs, have also been incorporated into different MOFs proving their capacity for imaging cells like MCF-7 or RAW264.7.^246,249^ Another interesting example demonstrates how the encapsulation of porous organic nanosheets (PONs) into the ZIF-8 MOF has enhanced the ability for labeling the HeLa and MCF-7 cells.^145^ Meanwhile the pristine nanosheets barely showed any luminescence, upon their encapsulation into ZIF-8 the living cancer cells were easily imaged (Figure 31).^145^

### 6.6 Anti-Counterfeiting

**Figure 32:**
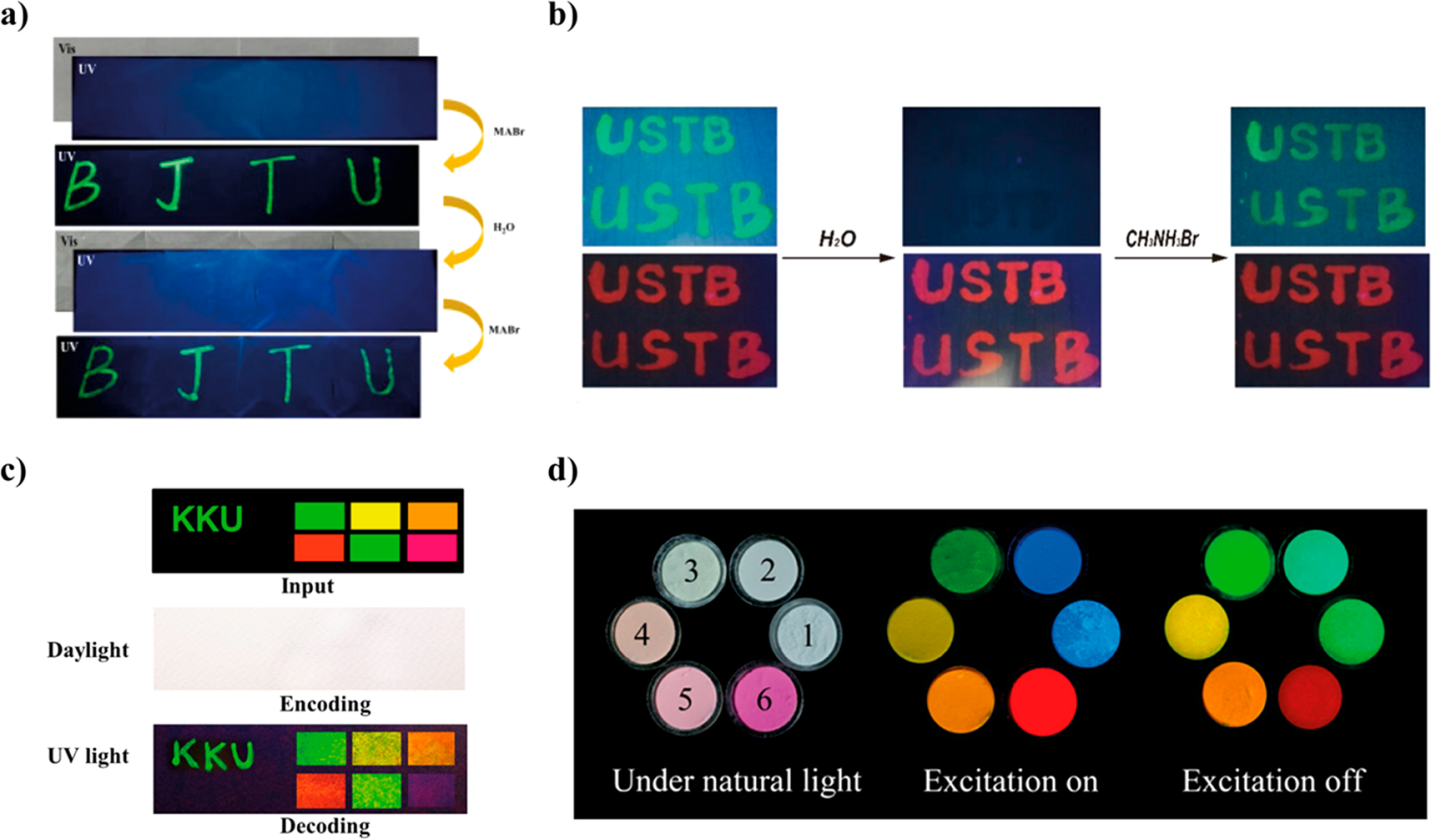
(a, b) Reversible switching of the luminescence of perovskite encapsulated within (a) UiO-66 (Adapted with permission from ref (250). Copyright 2020 Elsevier) and (b) Eu-BTC MOFs (Adapted with permission from ref (120). Copyright 2018 American Chemical Society) upon exposure to water and methylammonium bromide. (c) Image of code information (top image) and drawn output under daylight (transparent) and UV irradiation (decoding the information). Adapted with permission from ref (112). Copyright 2019 American Chemical Society. (d) Photographs under ambient light (left), UV excitation on (middle), and UV excitation off (right) of: 1= Cd(m-bdc)(bIm) MOF; 2 = 4-methylumbelliferone@Cd-MOF; 3 = Fluorescent Green B@Cd-MOF; 4 = Rhodamine 123@Cd-MOF; 5 = Rhodamine 6G@Cd-MOF; and 6 = Rhodamine B@Cd-MOF. Adapted with permission from ref (142). Copyright 2018 American Chemical Society.

LG@MOFs have been proposed as potential candidates for anticounterfeiting applications as they are capable of encrypting and decrypting the information by changing their luminescent properties. Below we shall consider examples that demonstrate how the use of inks made from luminescent perovskites encapsulated within the UiO-66 and Eu-BTC MOFs are promising materials for anticounterfeiting protection. The fact that perovskites are very sensitive to external factors (including water), confers them the ability to encrypt the information (turning-off the emission) simply through water exposure.^120,250^ This information can be recovered (turning-on the emission) when the material is impregned with a solution of methylammonium bromide (MABr) as shown in Figure 32a and b.^120,250^

Another interesting example consisting of carbon dots (CDs) within bimetallic lanthanoids MOFs has displayed the ability for encoding and decoding information by UV light exposure.^112^ Here, the written information was invisible under daylight, but it turns visible under UV light, keeping the color and information accurately to the expected input Figure 32c. Additionally, the authors demonstrate that it is possible to write information employing a styrene ink, which quenched the emission due to a photoinduced charge transfer mechanism. Interestingly, the information was erased after 1 h by a simple evaporation of the styrene, and it can be rewritten again, reducing in this way the waste of paper.^112^

A completely different approach for using LG@MOF against anticounterfeiting was based on materials exhibiting a long-lived luminescence emission. For instance, the encapsulation of different guests (4-methylumbelliferone, fluorescent green B, rhodamine 123, rhodamine 6G and rhodamine B) within the long-lived emissive Cd(m-bdc)(bIm) MOF, resulting in multiple composites which exhibit a luminescence that can last for a few seconds (Figure 32d).^142^ Furthermore, an ink containing RhB encapsulated within the Cd(m-bdc)(bIm) MOF was used to create a stamp that displays a red fluorescent emission under UV irradiation, but it transforms to a dark orange long-lived emission upon the removal of UV excitation, showing the potential of deploying such a material for anticounterfeiting applications.^142^

### 6.7 Light Emitting Devices (LEDs) and Optical Convertors

Light emitting diodes (LEDs) have emerged as one of the most efficient devices for light illumination and screens fabrication, reducing the global energy consumption, and therefore contributing to global sustainable growth. LEDs have many advantages over their traditional counterparts, such as the incandescent and halogen bulbs. For instance, the emission of light from LEDs is more directed, which reduce the use of diffusers and reflectors. Additionally, LEDs release 90% less heat than the incandescent bulbs while their life expectancy is 3 to 25 times longer, and most importantly, LEDs can save energy up to 80% more efficiently than the incandescent and halogens bulbs.^251^ Up until now, most of the commercial LEDs are fabricated with rare-earth elements, which are toxic and costly,^252,253^ and thus, there is an urgent need to replace these materials with greener alternatives.

LG@MOF has become a very promising alternative that attracted much attention during the past few years. Most of the examples reported hitherto are down-converter type LEDs, where typically a UV or blue LED is coated with the LG@MOF material to yield multicolor or white light emitting LEDs. Many examples can be found in the literature, where the luminescent guests can be of different types including organic fluorophores, perovskites, metal–organic complexes, and carbon dots.^42,54,70,75,77,78,112,122,136,254−256^ Even though this method has some advantages, as its simplicity and rapid production, there exists several drawbacks, like the plausible photodegradation of the materials owing to the high energy irradiation from the blue UV LEDs, which reduces the functional lifetime of the device. Having this in mind, an elegant alternative for the fabrication of MOF-LEDs has recently been demonstrated, which consists of the fabrication of a multilayered device in which the emissive layer is an electroluminescent LED.^37,225,257−260^ This field is still in its infancy, and there exists only a few examples of electroluminescent LG@MOF materials. In the first reported example, two organic fluorophores were entrapped in the pores of a Zr-based MOF (Zr-NDC).^257^ In this study, the authors fabricated three electroluminescent devices, in which the electroactive layers were the pristine Zr-NDC MOF, C153@Zr-NDC, and DCM@Zr-NDC, respectively. It was demonstrated that the pristine MOF was electroluminescent by itself, and that the incorporation of the organic fluorophores improves the emission efficiency and enables tuning of the emission color of the LED devices.^257^

**Figure 33:**
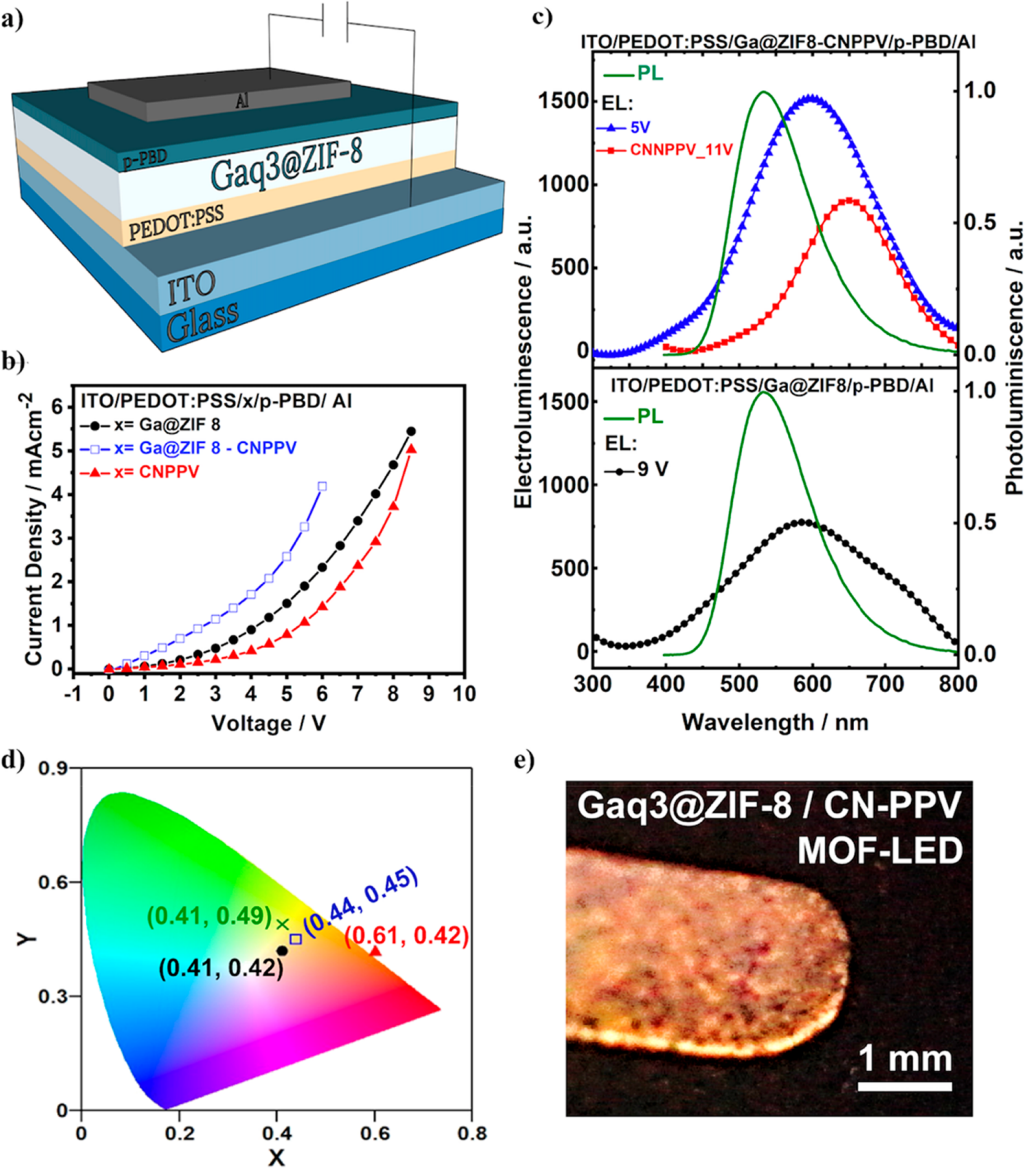
(a) Schematic representation of the multilayer MOF-LED architecture. (b) Current vs voltage curves of the different MOF LEDs and (c) their corresponding electroluminescent spectra. (d) CIE coordinates corresponding to the photoluminescence of Gaq_3_@ZIF-8 (green cross) and the electroluminescence of the different MOF-LEDs (Gaq_3_@ZIF-8/CNPPV, blue square; Gaq_3_@ZIF-8, black dot) and CNPPV-LED (red triangle). (e) Photograph of the MOF-LED device operating at 6 V, in which Gaq_3_@ZIF-8 serves as the electroluminescent layer with the characteristic orange-yellowish emission. Adapted from ref (37). Copyright 2020 John Wiley and Sons.

Another possible approach is to incorporate a “non-innocent” guest into the pores of an inert (nonelectroluminescent) MOF. Indeed, this is exactly the case of the confinement of the metal complex Gaq_3_, which is a well-known electroluminescent semiconductor, in the pores of the ZIF-8 host (Figure 33a).^37^ It was demonstrated that the Gaq_3_@ZIF-8 composite functions as the electroactive layer of the MOF-LED device. This material was sandwiched between different polymer layers (electron and hole transport layers), an ITO anode and an Al cathode (Figure 33b). The current vs voltage curves showed that the electroluminescence property was improved when the material was dispersed in CN-PPV [poly(5-(3,7-dimethyloctyloxy-2-methoxy-cyanoterephthalydiene], and that the turn-on voltage of the device was relatively low with a value of ∼3 V (Figure 33c). These recent exemplars are just proof-of-concept MOF-LED devices, where the efficiency of the devices is still low. Therefore, there is a huge margin for technical improvement, where combined knowledge from chemists, physicist, and engineers could help to boost this interdisciplinary subject.

**Figure 34:**
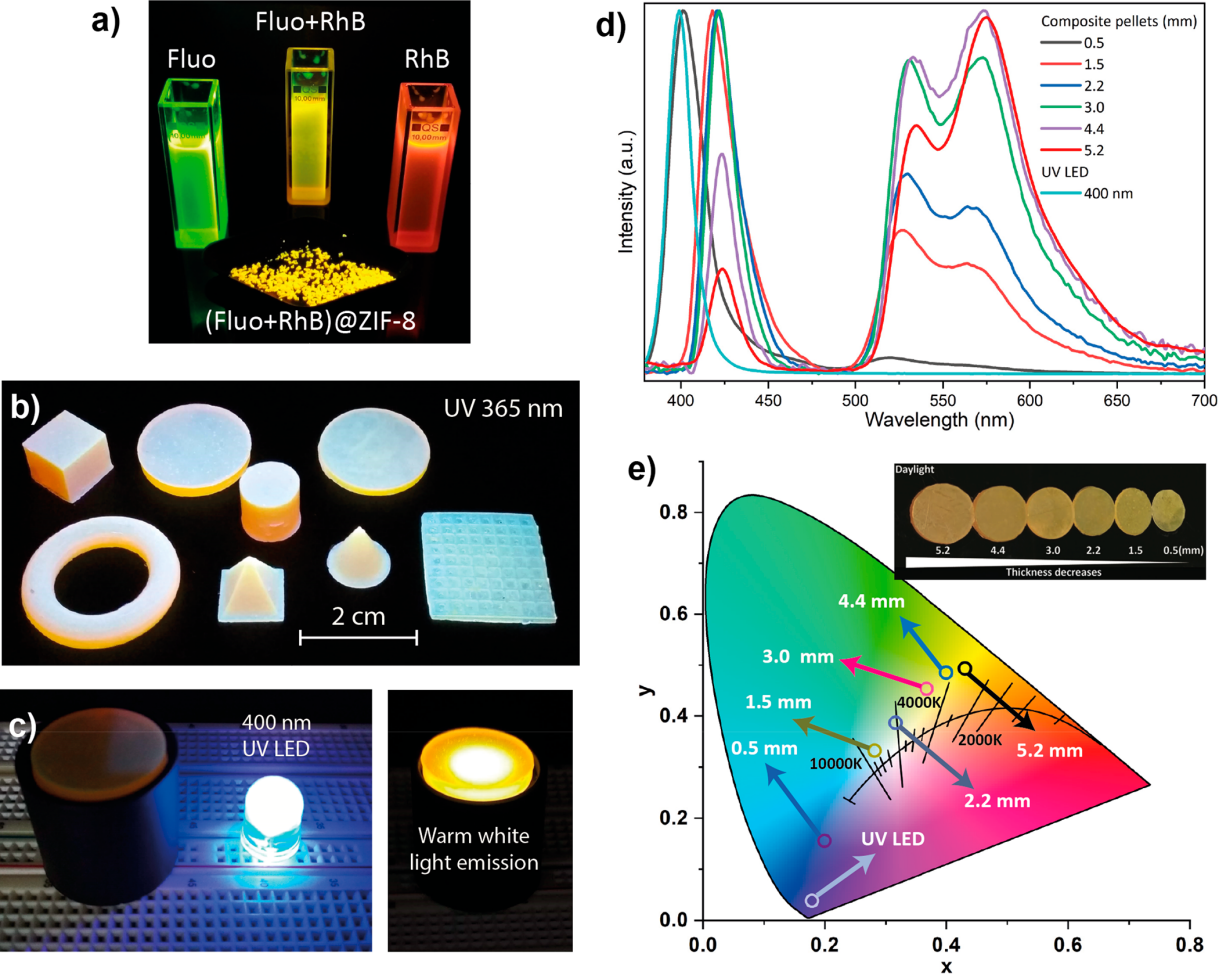
(a) Dual-guest@ZIF-8 material encapsulating a combination of fluorescein (Fluo) and rhodamine B (RhB) dyes to yield a bright yellow emission under 365 nm UV. (b) Various 3D-printed geometries viewed under UV. (c) Optical converter disc with a thickness of 3 mm producing a warm white light when excited by 400 nm UV source in transmission mode. (d, e) Emission spectra and CIE color chromaticity generated by a series of printed discs with a thickness of 0.5 to 5.2 mm (inset), tested using the optical conversion setup shown in panel (c). Adapted from ref (71). Copyright 2020 John Wiley and Sons.

Finally, we consider a case study on the manufacturing of a LG@MOF optical converter by employing a commercially available 3-D printing methodology. The composite material concerned is the dual-guest encapsulated system, termed (Fluo+RhB)@ZIF-8, which is a bright yellow emitter under UV irradiation (Figure 34a).^71^ The dual-guest nanomaterial was synthesized using the HCR method via a rapid one-pot reaction (Section 2.2), yielding crystals with a 2-D nanodisc morphology with a mean thickness of under 20 nm. The nanocrystals were then combined with a commercial-grade acrylic-based photopolymer (a blue emitter under UV), uniformly dispersed to yield a 3-D printable luminescent composite resin for constructing a range of geometries depicted in Figure 34b. For example, a 3 mm thick printed disc (Φ = 44%) can be used to build a warm white LED (down-conversion device) excited by a 400 nm UV source. Furthermore, it was demonstrated that by systematically adjusting the thickness of the 3-D printed discs from ∼0.5 to 5 mm, simple optical convertors can be engineered for tuning the emission color chromaticity starting from a cool white light all the way to a warm color emission (CCT range of 8300 K to 3700 K), see Figure 34e. This study suggests that the combination of stereolithography and other related high-resolution 3-D printing methodologies^261,262^ with LG@MOF-based nanomaterials could pave the way to the engineering and manufacturing of both precision and bulkier components for real-world applications.

## 7 Perspectives and Outlook

Encouragingly, the research on LG@MOF materials and their composite systems is gathering momentum. The literature is expanding swiftly, thus far generating a vast range of enticing photophysical and photochemical properties with interesting possibilities for innovative applications. The nanoconfinement or encapsulation of luminescent guests within a MOF host is a radical shift from the traditional route to yielding LMOF materials with intrinsic luminescence. In this review, we show that the LG@MOF concept is indeed highly versatile. In principle, it confers multiple pathways to design, fabricate, tune, and engineer the guest–host interactions for obtaining luminescent properties targeting bespoke applications, examples of which include intelligent optical sensors and photo switches, energy-efficient lighting, and stimuli-responsive photonics. As the number of reported LG@MOF systems and their potential applications continue to grow, it is timely to identify challenges surrounding this nascent field so as to motivate further systematic investigations from both the basic science and practical application standpoints.

### 7.1 Confinement Pathways

While general synthetic guidelines do exist to assist with identifying the possible pathways to encapsulate luminescence guests within a MOF host (e.g., *in situ* vs post synthetic confinement), the universality of the primary approaches is yet to be proven. The success of a LG@MOF synthesis is often reliant on multiple factors, beyond the straightforward choice of which guest species and compatible host to use. This is probably unsurprising from the perspective of MOF synthesis given the vast range of guest/host combinations which one can theoretically use, however, in practice, certain synthetic constraints do exist for successful fabrication of a desired LG@MOF system. On this matter, sample reproducibility is important and all details about the synthesis method and its scalability (or lack of) should be carefully reported, including caveats that might lead to a failed synthesis for a known system. For instance, thorough washing of the encapsulated sample is often overlooked, which may lead to luminescent guests adhering on the external surfaces and not truly confined inside the MOF structure. Of course, the washing step presents another problem for channel-type MOFs where the leaching of encapsulated guests is likely an issue. In practical terms, another challenge concerns the solvent wastes generated by solution-based synthesis and subsequent washing steps needed for HCR process and liquid impregnation routes. Gas-phase loading is solvent-free (guest sublimation and pore insertion) but may require a longer exposure time and specialist equipment (pressure and temperature control); nevertheless, the initial stage where the MOF host is synthesized typically requires the use of solvents. On this basis, the development of a greener synthetic pathway will be attractive, for instance mechanochemistry,^263^ although its general efficacy to confine luminescent guests is still relatively unknown.^264^ And, even if it delivers, this solvent-free approach will likely to be applicable to a specific combination of guest/host only but this is worth exploring.

### 7.2 Fluorescent Guest Loading

Quantitative determination of the amount of guest present in MOF host is often challenging; therefore, this information is not well described or missing from published works. NMR, TGA, EA, and UV–vis methods may be appropriate for estimating the amount of guest loading for a specific LG@MOF system. Connected to the first point above, to ensure reproducibility the reported values should make clear on how the calculations were made to arrive at the guest concentration claimed. However, the above techniques disregard whether the guest molecules are confined in the host structure/pores, or merely adhered on the external surfaces of the MOF parent crystals. The latter case is highly problematic for poorly washed samples, since the surface-bound guest species will inevitably cause an overestimation in quantification of the encapsulated-guest loading. To validate the success of the guest confinement/encapsulation strategy, this will require the use of complementary characterization techniques described in Section 4, especially the application of local-scale methodologies such as nanoFTIR, HRTEM, CLSM, and high-resolution tools available at the synchrotron and neutron facilities (spectroscopy, diffraction, and imaging).

### 7.3 Photodynamics and Mechanisms

Time-resolved photophysical measurements of LG@MOF materials is generally lacking in the literature. While excitation–emission spectra are commonplace, detailed photodynamics characterization of emission lifetime of the bulk samples (e.g., TCSPC) and at local scale (e.g., CLSM) are relatively uncommon for all reported LG@MOF systems. By untangling the different components of the multiexponential emission lifetimes this could reveal, for example, the formation of the H- and J-aggregates in addition to identifying contribution of the isolated monomers. Detailed photodynamic characterization could also shed light on unexpected energy transfer phenomenon associated with guest–host coupling, an improved understanding of which may allow one to control the luminescence behavior of LG@MOF and manipulate its response. More broadly, systematic investigations into the guest–host interactions and energy/charge transfer phenomenon are warranted, to help untangle the underpinning mechanisms governing the observed photobehavior. The mechanisms proposed for many of the reported studies concerned LG@MOFs are either vaguely explained or only speculative. The application of theoretical methods such as *ab initio* density functional theory (DFT) should be encouraged to study LG@MOF systems, where the main challenges lie in the modeling of spatial confinement of a large system with dispersion correction and their electronic structures, and simulation of excited-state events using the time-dependent DFT (TDDFT). To this end, the recent development and implementation of computationally efficient yet accurate DFT methods are promising to simulate the structure–property relationships of large Guest@MOF systems.^83,265^

### 7.4 Long-Term Materials Stability

Improved understanding should be sought about the photostability of LG@MOF systems subject to UV irradiation and other environmental factors. A limited number of accelerated photodegradation studies have suggested a likely enhancement in photostability of fluorophores, where a slower degradation rate was recorded upon guest confinement in MOF structure hence increasing their longevity.^83^ More systematic studies are warranted to investigate how the host framework may shield and protect the light-sensitive guest molecules to reduce or impede negative effects of photobleaching.^40,266^ Information on the long-term durability of LG@MOFs under ambient conditions is particularly lacking in literature, to study how the exposure to humidity and ambient temperature might alter material performance over time. For real-world applications, data will be needed to establish the influence of external stimuli (e.g., elevated temperature and pressure, stress, VOCs, pH) on sensor calibration and precision if a multiple-use device is intended.

### 7.5 Disruptive Sensors for Real-Time Detection

The development of LG@MOF composite systems has led to new materials potentially useful for turn-on luminescent sensors, setting them apart from the conventional class of turn-off type LMOFs. Encouragingly, several candidates for self-calibrating ratiometric sensors have also been demonstrated, where the dual-emissive properties may be harnessed to auto correct for external interferences in real time. Moreover, it was recently demonstrated that aggregation of AIE luminogens in MOF cages could yield a rare turn-on response subject to a mechanical stress, which is encouraging for mechanofluorochromic sensing application.^240^ These are highly promising research avenues where LG@MOFs have the edge over conventional systems that rely on intrinsic luminescence, but have not been explored with much depth. Yet another area that warrants further exploration is VOC sensing in vapor phase, which remains challenging for conventional materials but has been shown to be tractable adopting the concept of LG@MOF.^35^ Good selectivity (including water stability and resistance against ambient humidity), rapid response time, reversibility, and a turn-on response are among the challenges to be overcome to yield practical VOC vapor sensors.^267^

### 7.6 Electroluminescent Devices

In light of the promising proof-of-concept devices demonstrated so far,^37,225,257−260^ LG@MOF composites have the prospect of becoming an emergent class of photoactive materials to engineer the rare-earth-free and tunable electroluminescent MOF-LEDs. It will be essential to initiate concerted efforts in this central topic, aimed at improving the quantum yield and photostability of the electroactive layers, enhancing the device energy efficiency and increase its overall durability subject to real-world service conditions. Further to addressing the foregoing barriers, innovative advancements in downstream fabrication of LG@MOF-based applications and fine-scale device packaging will compel either creative adaptations of existing manufacturing methodologies or new developments of micro/nanofabrication inspired by latest advancements witnessed in high-resolution 3-D printing, precision inkjet printing, electrospinning, and lithography technologies that must be compatible with fluorescent guests, and organic and inorganic building units.

### 7.7 Mechanical Properties

Ultimately, there is the vital question of understanding the science to design and control the mechanical behavior of bespoke systems constituting LG@MOF crystals, polycrystalline films, bulk monoliths and hybrid composites. Central to “MOF mechanics” are a variety of problems encompassing: elasticity and flexibility, time-dependent viscous effects (creep and recovery), adhesion of dissimilar structures and debonding of interfaces, dynamics upon impact and mechanical dissipation, crack propagation and fracture. For example, establishing accurate structure-mechanical property relationships of LG@MOFs will be key to the engineering of mechano-sensors whose operation will rely on mechanofluorochromic effects. Recent developments seen in the adaptation of fine-scale mechanical characterization tools for probing MOF films and monoliths going beyond the elastic limit,^268−270^ bode well for the field. Systematic investigations are merited in the broader context of guest-encapsulated framework assemblies, while tackling the farseeing goal for enhancing mechanical resilience of sensors and devices destined for practical use.

In conclusion, the review shows that the fascinating concept of LG@MOF holds a tremendous promise for engineering unconventional fluorescent materials by nanoscale confinement, whose photophysics and photochemistry could yield revolutionary sensors, smart devices and next-generation lighting. The transformative aspects of LG@MOFs are creating a fertile ground perfect for vibrant interdisciplinary research, for which we envisage its growth will continue to accelerate for at least the next 10 years. We are optimistic that the numerous opportunities and challenges identified in this review will further entice and stimulate the multifaceted materials community, including (but not limited to) chemists, engineers, physicists, and computational scientists, to innovate and join forces to majorly advance this exciting research.

## Abbreviations

AFM: atomic force microscopy
AIE: aggregation induced emission
ANT: anthracene
ATR: attenuated total reflectance
Au: gold
bdc: 1,4-benzenedicarboxylate
BI: benzylidene imidazolinone
bIm: benzimidazole
BODIPY: boron-dipyrromethene
BPEI-CDs: branched poly(ethylenimine)-capped carbon quantum dots
BPT: biphenyl-3,4′,5-tricarboxylate
BSE: backscattered electron
C153: coumarin 153
C343: coumarin 343
C460: coumarin 460
C460: coumarin 460
CCD: charge coupled device
CCM: curcumin
CDs: carbon quantum dots
CLSM: confocal laser scanning microscopy
CN^–^: cyanide ions
CN-PPV: poly(5-(3,7-dimethyloctyloxy-2-methoxy-cyanoterephthalydiene
COK: Centrum voor Oppervlaktechemie en Katalyse
CT: charge transfer
Cyt *c*: cytochrome c
DAAC: 3,6-diaminoacridinium cation
DASP^+^: (4-p-(dimethylamino)styryl)-1-methylpyridinium
DBA: 9,10-dibenzoate anthracene
DCM: 4-(dicyanomethylene)-2-methyl-6-(4-dimethylaminostyryl)-4H-pyran
DFT: density functional theory
DHT: 2,5-dihydroxyterephthalic acid
DMA: dimethylacetamide
DMF: N,N-dimethylformamide
DXP: N,N-bis(2,6-dimethylphenyl)-3,4:9,10-perylene tetracarboxylic diimide
EA: elemental analysis
EDS/EDX/EDXS: energy dispersive X-ray spectroscopy
ESPT: excited state proton transfer
ESIPT: excited state intramolecular proton transfer
EXAFS: extended X-ray absorption fine structure
FIB: focused ion beam
FIR-53: Zn_2_(tipa)_2_(OH^–^)](NO_3_^–^)(SG7)_2/3_·5H_2_O
Fluo: fluorescein
FRET: Förster resonance energy transfer
FTIR: Fourier transform infrared
Gaq_3_: gallium(III) tris(8-hydroxyquinolinato)
GSH: glutathione
H_2_TZB: 4-(1H-tetrazol-5-yl)benzoic acid
HAADF: high-angle annular dark-field
HCR: high-concentration reaction
HPTS: 8-hydroxy-1,3,6-pyrenetrisulfonicacid trisodium
HRTEM: high-resolution transmission electron microscopy
HTATAB: 4,4′,4″-s-triazine-1,3,5-triyltri-*p*-aminobenzoic acid
ICP-OES: inductively coupled plasma optical emission spectroscopy
ICT: intramolecular charge transfer
Ir: iridium
K-M: Kubelka–Munk
LEDs: light emitting diodes
LG: luminescent guest
LMOF: luminescent MOF
Ln: lanthanide
MABr: methylammonium bromide
mIm: 2-methylimidazolate
MOF: metal–organic framework
MOL: metal–organic layer
nanoFTIR: nanoscale Fourier transform infrared spectroscopy
NCs: nanocrystals
NDC: 2,6-naphthalenedicarboxylate
NKU: Nankai University
nMOFs: nanometric MOFs
NMR: nuclear magnetic resonance
NO_2_-bdc: 2-nitro-4-benzenedicarboxylic acid
NR: Nile red
NTB: 4,4′,4″-nitrilotrisbenzoic acid
o-phen: 1,10-phenanthroline
O.D.: optical density
OLEDs: organic light-emitting diodes
OX: University of Oxford material
PCT: photoinduced charge transfer
PEG-ZnS: polyethylene glycol-capped ZnS
PONs: porous organic nanosheets
PTA: p-phthalic acid
PXRD: powder X-ray diffraction
q: 8-hydroxyquinoline
R6G: rhodamine 6G
Rh101: rhodamine 101
Rh6G: rhodamine 6G
RhB: rhodamine B
Rs: resorufin
Rubpy: Ru(II) tris(2,2′-bipyridine)
s-SNOM: scattering-type scanning near-field optical microscopy
SBE: backscattered electrons
SBMs: silica-based mesoporous systems
SC: single crystal
SCXRD: single crystal X-ray diffraction
SE: secondary electrons
SEM: scanning electron microscopy
SG7^3–^: solvent green 7
SR-IR: synchrotron-radiation infrared
STXM: scanning tunnelling X-ray microscopy
TCPP: 5,10,15,20-tetra(carboxyphenyl)porphyrin
TCSPC: time-correlated single photon counting
TDDFT: time-dependent density functional theory
TEA: triethylamine
TEM: transmission electron microscopy
TGA: thermogravimetric analysis
TIPA: tri(4-imidazolylphenyl)amine
TNP: 2,4,6-trinitrophenol
TPE: 1,1,2,2-tetraphenylethylene
tpt: 2,4,6-tri(4-pyridyl)-1,3,5-triazine
UiO: Universitetet i Oslo
UMCM: University of Michigan Crystalline Material
UV: ultraviolet
VOCs: volatile organic compounds
VTGs: VOCs, toxic chemicals and gases
XANES: X-ray absorption near edge structure
XAS: X-ray absorption spectroscopy
XPS: X-ray photoelectron spectroscopy
XRF: X-ray fluorescence
ZIF: zeolitic imidazolate framework
ZnQ: zinc(II) bis(8-hydroxyquinoline)
Φ: quantum yield
